# 5-Methoxybenzothiophene-2-Carboxamides as Inhibitors of Clk1/4: Optimization of Selectivity and Cellular Potency

**DOI:** 10.3390/molecules26041001

**Published:** 2021-02-13

**Authors:** Ahmed K. ElHady, Dalia S. El-Gamil, Po-Jen Chen, Tsong-Long Hwang, Ashraf H. Abadi, Mohammad Abdel-Halim, Matthias Engel

**Affiliations:** 1 Department of Pharmaceutical Chemistry, Faculty of Pharmacy and Biotechnology, German University in Cairo, Cairo 11835, Egypt; ahmed.elhady@guc.edu.eg (A.K.E.); daliashgamil@gmail.com (D.S.E.-G.); ashraf.abadi@guc.edu.eg (A.H.A.); mohammad.abdel-halim@guc.edu.eg (M.A.-H.); 2 School of Life and Medical Sciences, University of Hertfordshire Hosted by Global Academic Foundation, New Administrative Capital, Cairo 11865, Egypt; 3 Graduate Institute of Natural Products, College of Medicine, Chang Gung University, Taoyuan 333, Taiwan; pjchen@pu.edu.tw (P.-J.C.); htl@mail.cgu.edu.tw (T.-L.H.); 4 Department of Cosmetic Science, Providence University, Taichung 433, Taiwan; 5 Research Center for Chinese Herbal Medicine, Graduate Institute of Health Industry Technology, College of Human Ecology, Chang Gung University of Science and Technology, Taoyuan 333, Taiwan; 6 Department of Anesthesiology, Chang Gung Memorial Hospital, Taoyuan 333, Taiwan; 7 Department of Chemical Engineering, Ming Chi University of Technology, New Taipei City 243, Taiwan; 8 Pharmaceutical and Medicinal Chemistry, Saarland University, Campus C2.3, D-66123 Saarbrücken, Germany

**Keywords:** Clk1 inhibitor, pre-mRNA splicing, anticancer

## Abstract

Clks have been shown by recent studies to be promising targets for cancer therapy, as they are considered key regulators in the process of pre-mRNA splicing, which in turn affects every aspect of tumor biology. In particular, Clk1 and -4 are overexpressed in several human tumors. Most of the potent Clk1 inhibitors reported in the literature are non-selective, mainly showing off-target activity towards Clk2, Dyrk1A and Dyrk1B. Herein, we present new 5-methoxybenzothiophene-2-carboxamide derivatives with unprecedented selectivity. In particular, the introduction of a 3,5-difluoro benzyl extension to the methylated amide led to the discovery of compound **10b** (cell-free IC_50_ = 12.7 nM), which was four times more selective for Clk1 over Clk2 than the previously published flagship compound **1b**. Moreover, **10b** showed an improved growth inhibitory activity with T24 cells (GI_50_ = 0.43 µM). Furthermore, a new binding model in the ATP pocket of Clk1 was developed based on the structure-activity relationships derived from new rigidified analogues.

## 1. Introduction

Pre-mRNA splicing represents a critically important step for various processes such as development and differentiation. Recently, there has been increased interest in mutations of pre-mRNA splicing factors and their roles in oncogenesis of hematological as well as solid cancers [1]. High-frequency mutations of SRSF2 (serine/arginine-rich splicing factor 2) have been described in patients with myelodysplastic syndromes (MDS), chronic myelomonocytic leukemia and acute myeloid leukemia (AML), in addition to various mutations that have been found in splicing-related genes in lung, breast and pancreatic cancers. Therefore, pharmacological modulation of pre-mRNA splicing as a novel strategy in combating cancer has gained attention in the last decade [1].

Clks (cdc-like kinases) belong to the most essential regulators in the process of pre-mRNA splicing, which is achieved through their ability to phosphorylate and activate serine/arginine-rich (SR) proteins. Upon phosphorylation, SR proteins are released from nuclear speckles into the nucleoplasm, where they modulate the selection of splice sites during pre-mRNA processing [2]. Targeting Clks has great potential to serve as a novel treatment for cancer, as alternative splicing (pre-mRNA splicing modulation) has been found to affect nearly every aspect of tumor biology, including metabolism, apoptosis, cell cycle control, metastasis, invasion and angiogenesis (reviewed in [3]).

The Clk enzyme family consists of four members (Clk1, 2, 3 and 4) and belongs to the CMGC kinase branch of the human kinome. Both Clk1 and -4 have been shown to be frequently overexpressed kinases in a variety of human tumors [4], though Clk4 is much less investigated than Clk1. In a previous study, we identified compound **1b** (compound **21b** in [2]) as a potent and selective Clk1/4 inhibitor, with a slightly diminished potency against Clk2 [2]. Compound **1b** exhibited an unprecedented selectivity over Dyrk1A, a highly homologous kinase with functions in brain development but also in alternative splicing [5]. Unwanted co-inhibition of Dyrk1A should be avoided, since it has been reported to induce cardiac hypertrophy as a result of its role in antagonizing calcineurin signaling [6]; in addition, depending on the cellular context, Dyrk1A may have tumor-suppressive properties due to its role as a negative regulator of cell cycle progression at least in some cell types [7]. In our previous study, **1b** demonstrated great ability in inhibiting the growth of various types of tumor cells through selective depletion of cancer-relevant proteins, an effect that is mediated through the modulation of pre-mRNA splicing, which in turn leads to the nonsense-mediated decay of the mRNAs of genes that are essential for cell growth and survival [2]. Besides Clk1 and -4, Clk2 has also been identified as an anticancer target, especially in breast cancer [8]. Thus, depending on the tumor type, selective Clk1/4 or Clk2 inhibitors might be beneficial, whereas non-selective pan-Clk inhibitors might cause side effects due to the possibly strong impact on alternative splicing in all tissues.

Fused heteroaryl tricyclic scaffolds are among the most frequently reported Clk1 inhibitors, for example, those reported by Besson and co-workers [9], as well as those reported by the Moreau group and others [10,11,12]. Some of these tricyclic inhibitors display a significant growth inhibitory activity against various tumor cell lines; however, co-inhibition of related kinases besides Clk1 cannot be excluded, as selectivity data for some common off-targets are incomplete, e.g., regarding Dyrk1B, Dyrk2, haspin, CK2, STK17 and MLCK2 [13].

**Figure 1:**
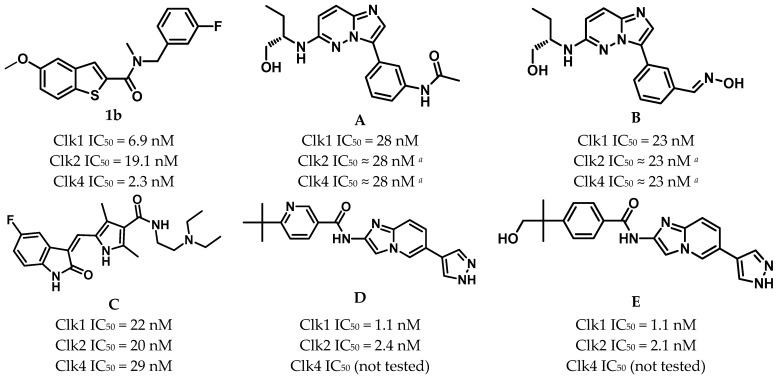
Potent Clk1 inhibitors described previously in the literature; 5-methoxybenzothiophene-2-carboxamide (**1b**) imidazo[1,2-b]pyridazines (**A**,**B**); Sunitinib (**C**); imidazo [1,2-*a*]pyridines (**D**,**E**). *^a^* No precise IC_50_s were provided; the authors stated that Clk2 and Clk4 were inhibited with potencies equal to that of Clk1.

The best chemical inhibitors described thus far inhibit Clk1, 2 and 4 with about the same potency; Nassima et al. succeeded in developing two highly potent imidazo[1,2-b]pyridazines (**A**, **B** in Figure 1) with IC_50_ values of 28 and 23 nM, respectively; both displayed equal potency against Clk2 and Clk4 kinases [14]. Moreover, Sunitinib (**C**, Figure 1), which is clinically approved for the treatment of imatinib-resistant gastrointestinal stromal tumor (GIST), renal carcinoma, and pancreatic neuroendocrine tumors [15], was reported by Murar et al. to be a highly potent Clk1 inhibitor with an IC_50_ of 22 nM, also showing high activity against Clk2 and Clk4, with IC_50_ values of 20 and 29 nM, respectively [16]. In addition, two imidazole[1,2-*a*]pyridine derivatives (Figure 1D,E) were identified by Araki et al. through high-throughput screening to be highly potent dual Clk1/Clk2 inhibitors, with an IC_50_ of 1.1 nM for both compounds against Clk1, and 2.4 and 2.1 nM, respectively, against Clk2; however, the inhibition of Clk4 was not tested for these derivatives [17].

The lack of isotype selectivity can be explained on the basis of the high homology between the isoenzymes: Both Clk1 and -4 share a 78.4% sequence identity [18], with both kinases harboring fully identical amino acid residues in and around their ATP binding pockets [2]. Moreover, Prak et al. also described a high degree of similarity between the catalytic domain of Clk1 and Clk2; indeed, the inhibitors reported in this study [19] and those cited above were equipotent against both kinases. Hence, it appears a challenging task to develop inhibitors specific for a certain isoform among the Clk enzyme family.

Herein we report the optimization of our previously reported benzo[b]thiophen-2-carboxamides [2] through systematic synthetic modifications of the amide linker and the benzyl extensions to the amide function, followed by a biological evaluation.

## 2. Results

**Figure 2:**
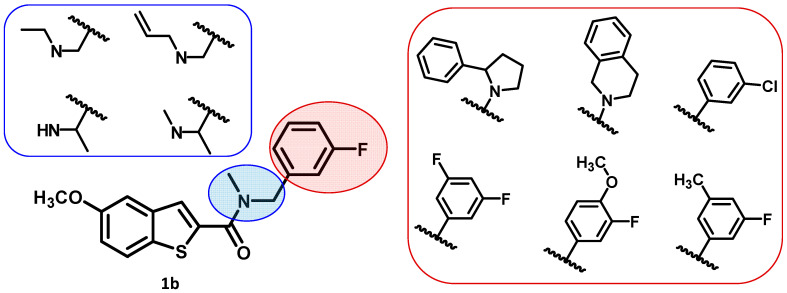
The planned structural modifications for the new series of compounds.

**Scheme 1:**
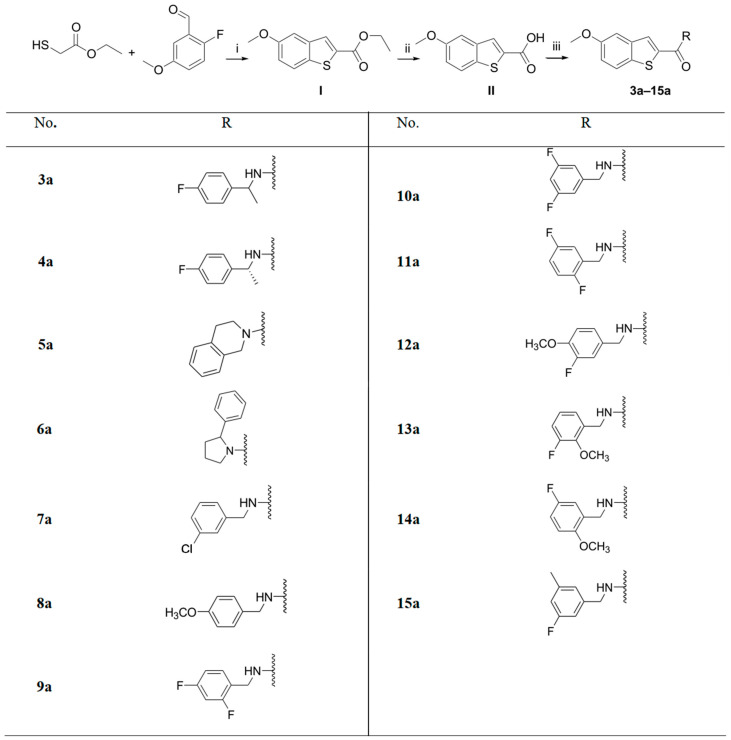
Synthesis of compounds **3a**–**15a**. Reagents and conditions: (i) K_2_CO_3_ in DMF at 0 °C, 1.2 eq. ethyl thioglycolate, 20 min, 1 eq. 2-fluoro-5-methoxybenzaldehyde in DMF, 70 °C, 4 h, % yield = 47%. (ii) 4 eq. KOH in ethanol/water, 80 °C, 3 h, % yield = 90%. (iii) 2 eq. HBTU, 4 eq. triethylamine, 4 eq. of the appropriate amine in DCM, room temperature, overnight, % yield = 18–97%.

To improve the potency of the previous 5-methoxybenzothiophene-2-carboxamide (**1b**, Figure 2) and its selectivity over the most common off-targets for Clk1 inhibitors [13], various structural modifications were applied, which included modifications at the amide linker as well as introduction of various structural extensions at the amide function (summarized in Figure 2). These aimed at the stabilization of the biologically active conformation of the benzyl moiety for Clk1 inhibition. In addition, the conformationally constrained analogues were also planned in order to verify and possibly refine the previously proposed binding mode. In parallel, many new mono- and di-substituted benzyl extensions were included, taking advantage of the favorable effect of the fluorine substituent in **1b**, while introducing additional groups for the interaction with the receptor but also for establishing intramolecular H-bonds (e.g., in **13a** and **14a** (Scheme 1), between the amide and the 2-methoxy substituent).

### 2.1. Chemistry

**Scheme 2:**
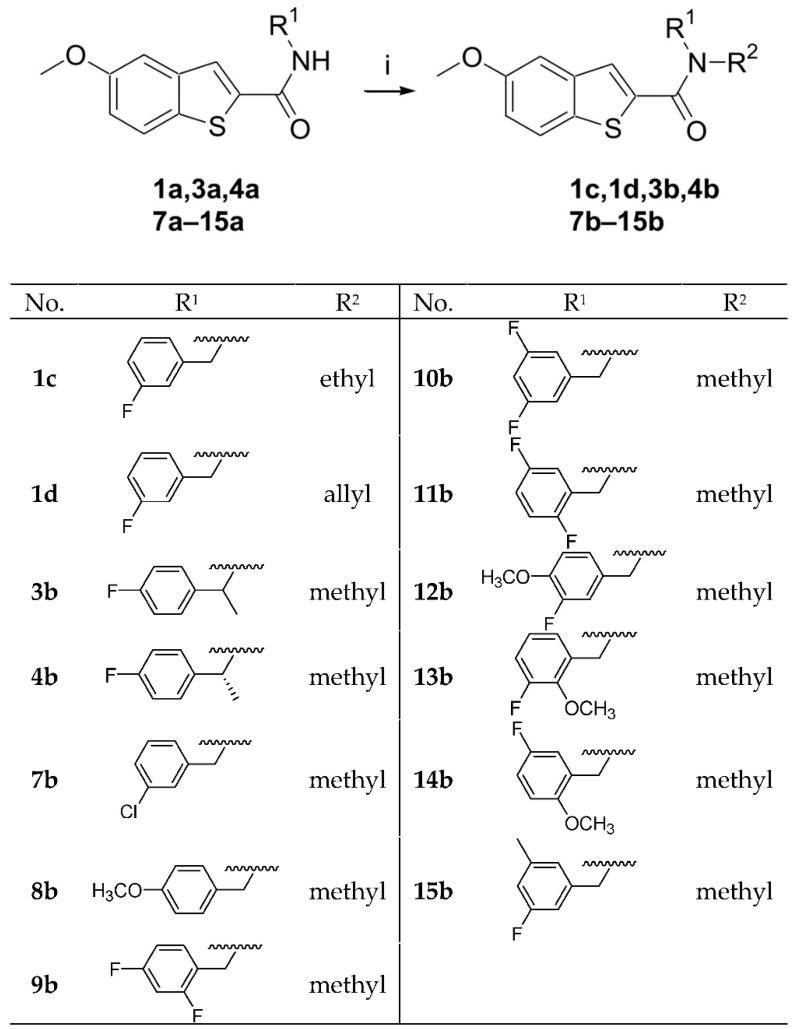
Synthesis of compounds **1c**, **1d**, **3b**, **4b**, **7b**–**15b**. Reagents and conditions: (i) 3 eq. KHMDS, 0 °C, 1 h, 1.5 eq. CH_3_I, or CH_3_CH_2_I or allyl bromide, room temperature, 24 h, %yield = 14–79%.

A three-step synthesis was employed to access the 5-methoxybenzothiophene-2-carboxamides (Scheme 1). Ethyl thioglycolate was reacted with 2-fluoro-5-methoxybenzaldehyde in the presence of potassium carbonate to produce the 5-methoxybenzothiophene-2-carboxylic acid ethyl ester (**I**) in a good yield. (**I**) was subjected to alkaline ester hydrolysis to produce the 5-methoxybenzothiophene carboxylic acid (**II**), which was then coupled with different amines in the presence of HBTU and trimethylamine to produce the final 5-methoxybenzothiophene-2-carboxamides (**3a–15a**). Alkylated amide derivatives (**1c**, **1d**, **3b**, **4b**, **7b–15b**) were synthesized through the deprotonation of the secondary amide using potassium bis(trimethylsilyl)amide (KHMDS) at 0 °C; this was followed by the addition of methyl iodide, ethyl iodide or allyl bromide to the trapped anion (Scheme 2).

### 2.2. Biological Evaluation and Development of a Binding Model

#### 2.2.1. In Vitro Clk1/Clk2 Inhibitory Activity

All the newly synthesized derivatives (compounds **1c**, **1d**, **3a–16a**, **3b**, **4b**, and **7b–15b**) were tested for their ability to inhibit Clk1 and Clk2 in vitro. With Clk1, the compounds were initially screened at a concentration of 100 nM in duplicates; with Clk2 the initial screening dose increased to 250 nM. IC_50_s were determined for compounds that displayed a percentage of inhibition higher than 50% in the initial screening, through testing a range of five concentrations with at least two replicates per concentration (Table 1 and Table 2). The Clk1 and Clk2 inhibitory activities of compounds **1a**, **2a**, **1b**, **2b** from our previous study are included in Table 1 for comparison.

#### 2.2.2. Development of a Binding Model Using Rigidified Analogues

**Figure 3:**
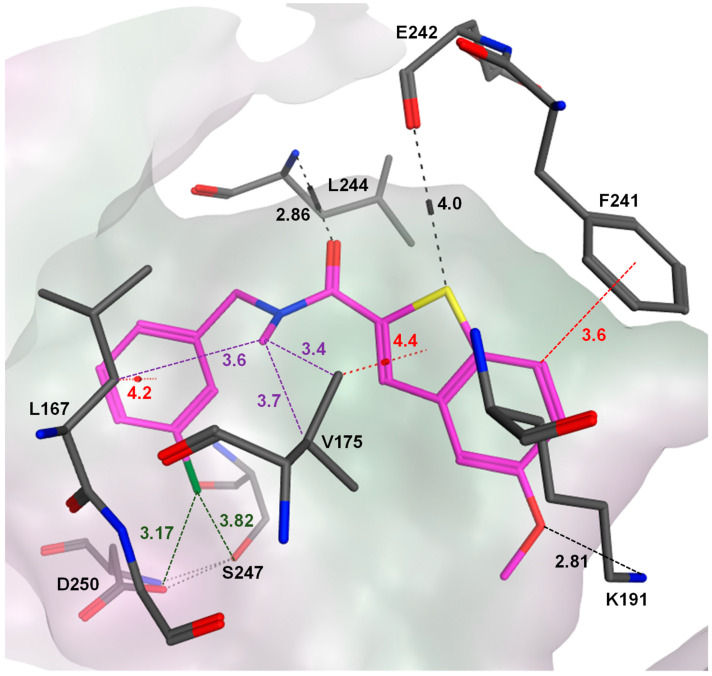
The predicted binding mode of compound **7b** (magenta) docked in the ATP binding pocket of Clk1 (PDB code of the coordinates: 1Z57) using MOE. **7b** was anchored inside the pocket through H-bonds with Leu244 and Lys191 (indicated in black), CH-π interactions with Leu167 and Val175 (red), an edge-to-face CH-π interaction with Phe241, hydrophobic interactions between the *N*-methyl, Leu167 and Val175 residues (violet), and halogen bonds (green) between the chlorine and the carbonyl groups of Ser247 as well as Asp250. Interactions are indicated by dashed lines, and distances between the heavy atoms are given in Å.

**Figure 4:**
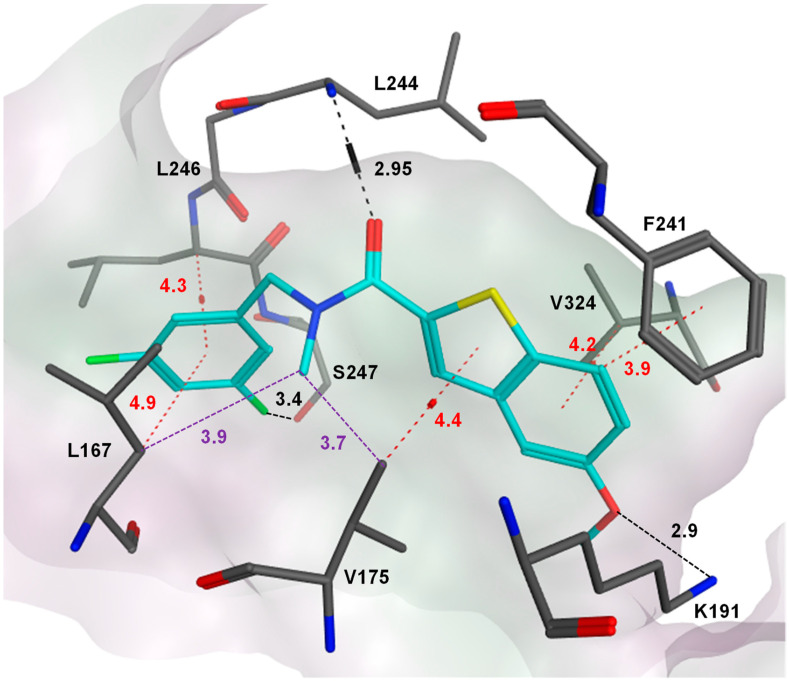
The predicted binding mode of compound **10b** (cyan). **10b** was docked in the ATP binding pocket of Clk1 (PDB code of the coordinates: 1Z57) using MOE. **10b** was anchored inside the pocket through H-bonds (indicated in black) with Leu244, Lys191 as well as a weaker H-bond interaction between the fluorine and Ser247. In addition, CH-π interactions (red) were predicted between the benzyl moiety and Leu167/Leu264, as well as between the benzothiophene core and Val175/Val324. An edge-to-face CH-π interaction with Phe241 and hydrophobic interactions (violet) between the *N*-methyl, Leu167 and Val175 were also predicted. All interactions are indicated by dashed lines, and distances between the heavy atoms are given in Å.

The strongly rigidified analogues **5a** and **6a** were synthesized to probe the biologically active conformation of our inhibitor class on the basis of our previously presented binding model of **1b** [2]. The dihedral angle of the benzyl moiety was fixed at two different degrees, which would direct the benzene ring either to a small hydrophobic cavity in the lid of the ATP pocket (in **6a**) or more planar toward the pocket exit (in **5a**), where favorable CH-π interactions might additionally boost the binding affinity. However, to our surprise, **5a** and **6a** were both inactive or only weakly active, respectively. This result suggested that the binding orientation predicted for **1b**, where the carbonyl interacted with Lys191, might not be preferred by all 5-methoxybenzothiophene-2-carboxamide analogues, because in such case either **5a** or **6a** should have exhibited a clear inhibitory potency. Already in previous docking runs, an inverted binding mode had occasionally been observed, where the ligand was flipped by 180° and the methoxy group interacted with Lys191. Hence, we decided to dock the most active and some inactive compounds of the present series and evaluated the consistency with the SAR in both potential binding modes. Indeed, we found that the SAR observed with the new compounds were all consistent with the inverted binding mode, where the amide carbonyl interacts with the Leu244-NH in the hinge region. Assuming this to be the generally preferred orientation of our ligands, the lack of activity observed with **5a** could eventually be explained by a predicted clash of the dihydro-isoquinoline moiety with the Leu167 side chain (Figure S1, Supplementary Material). With respect to the racemic probe compound **6a**, only the (*S*)-enantiomer could be placed in the ATP binding pocket at all; however, this was at the cost of several inter- and intramolecular steric clashes, explaining the very low activity (Figure S2B, Supplementary Material). Apparently, the original binding mode, with H-bonding between the carbonyl and Lys191, has other energetic disadvantages, resulting in a low binding affinity and precluding inhibition. Furthermore, the originally published binding mode would not be in accordance with the loss of potency found with **1d**, **3a** (*S*), **9b** and **13b**, while this could be explained unambiguously by the flipped binding orientation. Altogether, the SAR obtained with the entire set of the new, challenging modifications made it possible to derive a new binding model for the compounds presented here (cf. Figure 3 and Figure 4), which will be employed in the following as a basis for the discussion.

#### 2.2.3. Variation of the *N*-alkylation and Methylation of the Benzyl Position

In our previous study, it was reported that *N*-methylation of the amide greatly enhanced the inhibitory activity against Clk1 [2] (cf. **1a** vs. **1b**). This overall tendency was confirmed with all unmethylated/ *N*-methylated compound pairs of the present series, too. However, when the methyl was enlarged to ethyl and allyl (compounds **1c** and **1d**, respectively, Table 1), no further enhancement, but rather a marked drop of activity was noted, suggesting that the available space around the methyl group is rather limited. This observation is in accordance with the binding models for the potent compounds **7b** and **10b**, which were derived later (cf. Figure 3 and Figure 4).

The *p*-fluoro analogues having the methyl group added to the benzylic carbon spacer seemed to be more potent than the unmethylated and *N*-methylated congeners (compare **2a** and **2b** with **4a**, Table 1). However, this was only true for the (*R*) enantiomer (**4a**), since the lower activity of the corresponding racemate (**3a**) indicated that the (*S*) enantiomer was inactive, probably due to a steric collision of the methyl group with Leu244 (cf. binding model in Figure S2A). In contrast, there is more space for the methyl in the (*R*) enantiomer (**4a**), as it will point away from Leu244.

On the other hand, combining a methyl group at the benzylic carbon spacer with an *N*-methylation completely abolished the inhibitory activity, irrespective of the stereochemistry (compounds **3b** and **4b**, Table 1). It can be assumed that the additional methyl group at the benzylic carbon spacer is forced to adopt an anti or gauche conformation relative to the *N*-methyl, thus strongly increasing the steric demands which would prevent binding due to similar reasons as with **3a** (*S*) (cf. Figure 3).

#### 2.2.4. Variation at the Benzyl Amide Function

In the next optimization step, various new moieties were tested as an extension to the amide function, including mono- and di-substituted benzyl derivatives. Compound **7b**, having the bulkier and more lipophilic *m*-chloro substituent on the phenyl ring, was equipotent to compound **1b**; however, **7b** was more selective for Clk1 over Clk2 (selectivity factor = 16) than **1b** (selectivity factor = 2.7). The high potency of **7b** could be explained based on our new binding model, predicting that CH-π interactions were established with Leu167 and Leu246 via the *m*-chloro phenyl moiety, and that halogen bonds were formed involving the chlorine, Ser247 and Asp250 (Figure 3).

In our previous study, it was reported that using an electron withdrawing fluoro substituent at the phenyl ring was more favorable for the potency than an electron donating methyl or methoxy substituent at the *meta*-position; however, a closer investigation in the present study revealed that the effects were dependent on the substitution positions: fluorine was only favorable in the *meta*-position of the phenyl ring, while in the *para*-position, a methoxy substituent was superior to fluorine (compare **2b** with **8b**). The latter finding was in accordance with our binding model, where the predicted CH-π interactions involving Leu167 and Leu246 basically preferred an electron-rich phenyl ring; it is, therefore, likely that fluorine only becomes favorable if it can undergo additional interactions, such as an H-bond acceptor to Ser247 (cf. Figure 4). Based on these considerations, we decided to test the effect of disubstituted benzyl extensions, by combining fluorine with an electron donating group but also by introducing a second fluorine at different positions. With respect to the di-fluoro substitutions, the presence of one fluorine at the *meta*-position was essential for Clk1 inhibitory activity (compare **9b** with **10b** and **11b**, Table 2). Installing both fluorine atoms at the *meta*-position was optimum among the di-fluorinated rings regarding Clk1 inhibitory activity (**10b**). The importance of the *m*-fluoro substituent could be explained by comparing the binding models of **10b** (Figure 4) with that of **9b** (Supplementary Materials, Figure S3) in the Clk1 ATP binding pocket; despite forming a CH-π interaction with Leu167, **9b** failed to establish an H-bond interaction with Leu244 in the hinge region, and it was evident that the H-bond with Ser247 can only form with fluorine in the *meta*-position, like in **10b** (Figure 4). Of note, despite being slightly less potent (IC_50_ = 12.7 nM) than **1b**, **10b** was more selective for Clk1 over Clk2 (selectivity factor = 10). Regarding the combinations of fluorine with an electron withdrawing group on the phenyl ring, the 3-fluoro-4-methoxy (**12b**) and the 3-fluoro-5-methyl (**15b**) disubstituted benzyl rings were most beneficial for the Clk1 inhibitory activity. **15b** also exhibited a high selectivity for Clk1 over Clk2 (15-fold), although it was slightly less potent than **7b** against purified Clk1 (IC_50_s = 10.9 nM and 6.2 nM, respectively).

#### 2.2.5. Inhibition of Tumor Cell Growth In Vitro

In our previous study, the bladder carcinoma cell line T24 was identified as a sensitive system for testing the cellular activity of Clk1/4 inhibitors, where **1b** showed a GI_50_ value of 0.63 µM [2]. This was in accordance with the Oncomine data from several studies (Oncomine.org) which had revealed Clk1 mRNA overexpression in bladder cancer. Therefore, we decided to evaluate the cellular activity of the most potent and selective Clk1 inhibitors among the new series (**7b**, **10b**, **12b** and **15b**) against the T24 cell line (Table 3).

In this assay, **10b** emerged as the most potent inhibitor of T24 cell growth, exhibiting higher potency than **1b**, despite being somewhat less potent in the cell-free assay. There was no strict correlation between the cell-free vs. cellular potencies, suggesting that pharmacokinetic properties, such as cellular uptake, played a major role for the efficacy in cells. Obviously, the mono- and difluoro-substitution in **1b** and **10b** strongly improved the physicochemical properties of the 5-methoxybenzothiophene-2-carboxamide scaffold so that the compounds became more available to the cellular Clk1. Since the selectivity of **10b** had increased compared with the reference compound **1b** (cf. Table 4), it was less likely that the inhibition of additional kinases was responsible for the higher cellular activity, though it cannot be totally ruled out.

#### 2.2.6. Kinase Selectivity Profiling

Having identified **10b** as the most potent inhibitor in T24 cells, it was important to further analyze the selectivity of this compound against a larger panel of kinases that were frequently reported as off-targets for Clk1 inhibitors [13,20,21,22,23,24], due to the highly similar ATP binding pockets. The results are shown in Table 4.

The co-inhibition of Clk4 by **10b** was expected, since Clk1 and Clk4 share the highest sequence identity among the Clk family members. In view of their fully identical ATP binding pocket [2], selective inhibition of Clk1 vs. Clk4 and vice versa might not be achievable by ATP competitive compounds. Among the tested non-Clk kinases, only Dyrk1A and Dyrk1B were appreciably inhibited; however, the IC_50_ determination for Dyrk1A revealed that **10b** was 28 times more potent for Clk1; this selectivity factor was higher than that reported for many highly potent Clk1 inhibitors in the literature: e.g., the quinazoline derivatives **34** and **35** described in [25], with selectivity factors of 2.8 and 2.7 respectively; the aminopyrimidine derivatives **15** and **17** reported in Ref. [26], with selectivity factors of 3.25 and 2, respectively; the azaindole derivative **10c** reported in Ref. [27], with a selectivity factor of 3.2; TG003 reported in [28], which showed higher potency against Dyrk1A than against Clk1 (IC_50_s of 12 and 20 nM, respectively); and KH-CB19 reported in [29], with a selectivity factor of 2.8.

## 3. Materials and Methods

### 3.1. Chemistry

Solvents and reagents were obtained from commercial suppliers and used as received. Melting points were determined on a Stuart SMP3 melting point apparatus. A Bruker DRX 500 spectrometer was used to obtain the ^1^H-NMR and ^13^C-NMR spectra. The chemical shifts are referenced to the residual protonated solvent signals. All final compounds had a percentage purity of at least 95%, and this could be verified using HPLC coupled with mass spectrometry. Mass spectra (HPLC−ESIMS) were obtained using a TSQ quantum (Thermo Electron Corp., Waltham, MA, USA) instrument prepared with a triple quadrupole mass detector (Thermo Finnigan, Waltham, MA, USA) and an ESI source. All samples were injected using an autosampler (Surveyor, Thermo Finnigan) by an injection volume of 10 μL. The MS detection was determined using a source CID of 10 V and carried out at a spray voltage of 4.2 kV, a nitrogen sheath gas pressure of 4.0 × 105 Pa, a capillary temperature of 400 °C, a capillary voltage of 35 V, and an auxiliary gas pressure of 1.0 × 105 Pa. The stationary phase used was an RP C18 NUCLEODUR 100-3 (125 mm × 3 mm) column (Macherey & Nagel, Düren, Germany). The solvent system consisted of water containing 0.1% TFA (A) and 0.1% TFA in acetonitrile (B). The HPLC method used a flow rate of 400 μL/min. The percentage of B started at 5%, increased up to 100% during 7 min, was kept at 100% for 2 min, and was flushed back to 5% in 2 min and was kept at 5% for 2 min. Melting points were determined using a BUCHI B-540 melting point apparatus and are uncorrected. The high-resolution mass analyses were performed using a Orbitrap Q exactive mass spectrometer, equipped with a heated ESI source and an quadrupole-orbitrap coupled mass detector and an Ultimate3000 HPLC (Thermo Finnigan, San Jose, CA, USA). The MS detection was carried out at a spray voltage of 3.5 kV, a nitrogen sheath gas pressure of 4.0 × 10^5^ Pa, an auxiliary gas pressure of 1.0 × 10^5^ Pa and a capillary temperature of 300 °C. All samples were injected by autosampler with an injection volume of 15 μL. A RP Nucleoshell Phenyl-hexyl^®^ (100-3, 2,7 µm) column (Macherey-Nagel GmbH, Dühren, Germany) was used as stationary phase. The solvent system consisted of 0.1% formic acid (A) and 0.1% formic acid in acetonitrile (B). HPLC-Method: Flow rate 550 μL/min. The percentage of B started at an initial of 5%, increased up to 40% during 10 min, then to 99% during the next 4 min, kept at 99% for 2 min and flushed back to the initial 5%. Xcalibur software was used for data acquisition and plotting.

#### 3.1.1. General Synthetic Procedures

##### Procedure A, Synthesis of 5-Methoxybenzo[b]thiophene-2-carboxylic Acid Ethyl Ester (**I**)

To an ice-cooled suspension of (7.2 g, 2 eq.) of K_2_CO_3_ in DMF (20 mL) was added ethyl thioglycolate (31.2 mmol, 1.2 eq.). The reaction mixture was stirred for 20 min at 0 °C under nitrogen, this was followed by the gradual addition of 2-fluoro-5-methoxybenzaldehyde (26 mmol, 1 eq.) in DMF (10 mL). The mixture was heated to reflux at 70 °C for 4 h. Afterwards, the suspension was cooled to room temperature and poured over 100 mL of 2 M HCl, the aqueous layer was extracted by DCM (5 × 10 mL), and the combined organic layers were thoroughly washed with water and brine, dried over anhydrous MgSO_4_, evaporated under reduced pressure and the resulting residue was purified by column chromatography (CC) using a solvent system of (petroleum ether/ DCM 3:2), giving the pure carboxylic acid ethyl ester as a yellow solid in a yield of 2.9 g (47%); ^1^H-NMR (500 MHz, DMSO-*d_6_*) δ 8.09 (s, 1H), 7.93 (d, *J* = 8.9 Hz, 1H), 7.53 (d, *J* = 2.5 Hz, 1H), 7.17 (dd, *J* = 8.9, 2.6 Hz, 1H), 4.34 (q, *J* = 7.1 Hz, 2H), 3.82 (s, 3H), 1.32 (t, *J* = 7.1 Hz, 3H); ^13^C-NMR (126 MHz, DMSO-*d_6_*) δ 161.98, 157.48, 139.64, 133.92, 133.80, 130.35, 123.75, 118.04, 107.07, 61.36, 55.33, 14.11.

##### Procedure B, Synthesis of 5-Methoxybenzo[b]thiophene-2-carboxylic Acid (**II**)

The ethyl ester (**I**) was suspended in a mixture of 50 mL of ethanol and 25 mL of water; this was followed by the addition of 4 eq. KOH. The mixture was heated to reflux at 80 °C for 3 h. Afterwards, the solvent was removed under reduced pressure, and the resulting aqueous layer was cooled to 0 °C, followed by the gradual addition of conc. HCl until the pH was adjusted to 1. The aqueous layer was then extracted using DCM (5 × 50 mL), the combined organic extracts were filtered over anhydrous MgSO_4_, and evaporated under reduced pressure giving the carboxylic acid derivative as a white solid in a yield of 2.3 g (90%). ^1^H-NMR (500 MHz, DMSO-*d_6_*) δ 8.01 (d, *J* = 0.6 Hz, 1H), 7.91 (d, *J* = 8.9 Hz, 1H), 7.52 (d, *J* = 2.5 Hz, 1H), 7.15 (dd, *J* = 8.9, 2.6 Hz, 1H), 3.82 (s, 3H); ^13^C-NMR (126 MHz, DMSO-*d_6_*) δ 163.51, 157.38, 139.84, 135.64, 133.82, 129.90, 123.73, 117.74, 106.93, 55.33.

##### Procedure C, General Procedure for the Synthesis of 5-Methoxybenzo[b]thiophene-2-carboxylic Acid Amide Derivatives

5-Methoxybenzo[b]thiophene-2-carboxylic acid (**II**) (0.1 g, 0.5 mmol) was dissolved in DCM (30 mL), then 0.3 g of HBTU and 2 mL of TEA were added. The reaction mixture was stirred for 30 min, following this, the appropriate amine (4 eq.) was added dropwise, and the reaction mixture was stirred at room temperature overnight. The solvent was evaporated under reduced pressure. The residue was partitioned between 50 mL of ethyl acetate and 20 mL of water then the aqueous layer was extracted with three 20 mL portions of ethyl acetate. The combined organic extracts were filtered over anhydrous MgSO_4_, the solvent was removed under reduced pressure, and the product was purified by CC to give the 5-methoxybenzo[b]thiophene-2-carboxylic acid amide derivatives in different yields.

##### Procedure D, General Procedure for the Synthesis of 5-Methoxybenzo[b]thiophene-2-carboxylic Acid Alkyl Amide Derivatives

The respective amide derivative was dissolved in dry THF and stirred at 0 °C, this was followed by the gradual addition of 3 eq. of potassium bis(trimethylsilyl)amide (0.5 M solution in THF), the reaction mixture was left to stir for 1 h, after which methyliodide or ethyliodide or allylbromide (1.5 eq.) were added. The reaction mixture was left to stir for 24 h at room temperature, afterwards, the solvent was removed under reduced pressure, then a small amount of water was added, and extraction was carried out using DCM (5 × 30 mL). The organic layers were combined, dried over anhydrous MgSO_4_, the solvent was removed under reduced pressure and the product was purified by CC or by salting out to give the 5-methoxybenzo[b]thiophene-2-carboxylic acid alkyl amide derivatives in different yields.

#### 3.1.2. Experimental Details and Synthesis of the Compounds

*N-(3-fluorobenzyl)-5-methoxybenzothiophene-2-carboxamide* (**1a**). The experimental details and synthesis of the compound have been previously reported in Ref. [2], annotated as compound **21a**.

*N-Methyl-N-(3-fluorobenzyl)-5-methoxybenzothiophene-2-carboxamide* (**1b**). The experimental details and synthesis of the compound have been previously reported in Ref. [2], annotated as compound **21b**.

*N-Ethyl-*N*-(3-fluorobenzyl)-5-methoxybenzothiophene-2-carboxamide* (**1c**). The compound was synthesized according to procedure D using compound **1a** and ethyliodide to give a yellow oil: yield (21%). The product was purified by CC (DCM); ^1^H-NMR (500 MHz, CDCl_3_) δ 7.69 (d, *J* = 8.8 Hz, 1H), 7.43 (s, 1H), 7.35 (dd, *J* = 12.9, 6.4 Hz, 1H), 7.20 (s, 1H), 7.10 (d, *J* = 7.5 Hz, 1H), 7.07–6.98 (m, 3H), 4.79 (s, 2H), 3.85 (s, 3H), 3.55 (q, *J* = 7.1 Hz, 2H), 1.25 (t, *J* = 7.1 Hz, 3H); ^13^C-NMR (126 MHz, CDCl_3_) δ 165.5, 163.6 (d, ^1^*J*_C−F_ = 246.9 Hz), 158.2, 140.1, 138.6, 133.2, 130.8, 125.1, 123.71 (d, ^2^*J*_C−F_ = 20.4 Hz), 123.4, 122.5 (d, ^3^*J*_C−F_ = 4.0 Hz), 117.0, 114.9 (d, ^2^*J*_C−F_ = 21.0 Hz), 114.7, 106.5, 55.9, 53.8, 30.1, 1.4; (ESI-MS) *m/z* = 344.16 [M + H]^+^.

*N-Allyl-*N*-(3-fluorobenzyl)-5-methoxybenzothiophene-2-carboxamide* (**1d**). The compound was synthesized according to procedure D using compound **1a** and allylbromide to give a yellow oil: yield (78.9%). The product was purified by CC (DCM/Petroleum ether 2:1); ^1^H-NMR (500 MHz, CDCl_3_) δ 7.67 (d, *J* = 8.8 Hz, 1H), 7.47 (d, *J* = 8.6 Hz, 1H), 7.32 (d, *J* = 6.4 Hz, 1H), 7.18 (s, 1H), 7.08 (d, *J* = 7.4 Hz, 1H), 7.05–6.96 (m, 3H), 5.88 (dtd, *J* = 15.7, 10.5, 5.4 Hz, 1H), 5.32 (d, *J* = 9.8 Hz, 1H), 5.24 (d, *J* = 16.1 Hz, 1H), 4.75 (s, 2H), 4.10 (d, *J* = 5.3 Hz, 2H), 3.83 (s, 3H); ^13^C-NMR (126 MHz, CDCl_3_) δ 165.3, 163.1 (d, ^1^*J*_C−F_ = 247.0 Hz), 157.8, 143.2, 139.7, 139.4 (d, ^3^*J*_C−F_ = 6.9 Hz), 137.9, 132.9, 132.5 (d, ^3^*J*_C−F_ = 7.0 Hz), 130.4 (d, ^2^*J*_C−F_ = 10.3 Hz), 124.9, 123.8, 123.0, 118.3, 116.8, 114.6 (d, ^2^*J*_C−F_ = 21.2 Hz), 106.2, 55.5, 30.3, 29.4; (ESI-MS) *m/z* = 356.1 [M + H]^+^.

*N-(4-fluorobenzyl)-5-methoxybenzothiophene-2-carboxamide* (**2a**). The experimental details and synthesis of the compound have been previously reported in Ref. [2] **2**, annotated compound as **22a**.

*N-Methyl-N-(3-fluorobenzyl)-5-methoxybenzothiophene-2-carboxamide* (**2b**). The experimental details and synthesis of the compound have been previously reported in Ref. [2], annotated as compound **22b**.

*(RS)-N-(1-(4-fluorophenyl)ethyl)-5-methoxybenzothiophene-2-carboxamide* (**3a**). The compound was synthesized according to procedure C using (*RS*)-1-(4-fluoro-phenyl)-ethylamine to give a white solid: yield (43.7%); The product was purified by CC (DCM); mp 163.3–164.3 °C; ^1^H-NMR (500 MHz, DMSO-d_6_) δ 9.08 (d, *J* = 8.0 Hz, 1H), 8.11 (d, *J* = 0.6 Hz, 1H), 7.91–7.84 (m, 1H), 7.46–7.41 (m, 3H), 7.19–7.13 (m, 2H), 7.10 (dd, *J* = 8.9, 2.5 Hz, 1H), 5.14 (quint, *J* = 7.1 Hz, 1H), 3.83 (s, 3H), 1.49 (d, *J* = 7.1 Hz, 3H); ^13^C-NMR (126 MHz, DMSO-*d_6_*) δ 161.0 (d, ^1^*J*_C-F_ = 242.1 Hz), 160.7, 157.3, 140.9, 140.6 (d, ^4^*J*_C-F_ = 3.0 Hz), 140.2, 132.6, 128.0 (d, ^3^*J*_C-F_ = 8.1 Hz), 124.7, 123.5, 116.5, 114.9 (d, ^2^*J*_C-F_ = 21.2 Hz), 106.7, 55.3, 48.0, 22.1; (ESI-MS) *m/z* = 330.16 [M + H]^+^.

*(RS)-N-(1-(4-fluorophenyl)ethyl)-N-methyl-5-methoxybenzothiophene-2-carboxamide* (**3b**). The compound was synthesized according to procedure D using compound 3a and methyliodide to give a pale yellow solid: yield (40.6%); the product was purified by CC (DCM); mp 123.7 °C; ^1^H-NMR (500 MHz, DMSO-*d_6_*, 373 Kelvin) δ 7.84 (d, *J* = 8.8 Hz, 1H), 7.65 (s, 1H), 7.43 (d, *J* = 2.4 Hz, 1H), 7.41–7.35 (m, 2H), 7.22–7.15 (m, 2H), 7.09 (dd, *J* = 8.8, 2.5 Hz, 1H), 5.72 (q, *J* = 6.9 Hz, 1H), 3.84 (s, 3H), 2.87 (s, 3H), 1.61 (d, *J* = 7.0 Hz, 3H); ^13^C-NMR (126 MHz, DMSO-*d_6_*, 373 Kelvin) δ 163.44, 161.02 (d, ^1^*J*_C-F_ = 243.8 Hz), 157.22, 139.50, 138.33, 136.03 (d, ^4^*J*_C-F_ = 3.1 Hz), 131.53, 128.39 (d, ^3^*J*_C-F_ = 8.1 Hz), 124.56, 122.59, 115.76, 114.70 (d, ^2^*J*_C-F_ = 21.4 Hz), 106.83, 55.11, 52.81, 29.74, 16.06; (ESI-MS) *m/z* = 344.18 [M + H]^+^.

*(R)-N-(1-(4-fluorophenyl)ethyl)-5-methoxybenzothiophene-2-carboxamide* (**4a**). The compound was synthesized according to procedure C using *(R)-*1-(4-fluoro-phenyl)-ethylamine to give a white solid: yield (44.4%); the product was purified by CC (DCM); mp 150 -151 °C; ^1^H-NMR (500 MHz, DMSO-*d_6_*) δ 9.08 (d, *J* = 8.0 Hz, 1H), 8.11 (d, *J* = 0.5 Hz, 1H), 7.88 (d, *J* = 8.9 Hz, 1H), 7.46–7.41 (m, 3H), 7.19–7.13 (m, 2H), 7.10 (dd, *J* = 8.9, 2.5 Hz, 1H), 5.14 (quint, *J* = 7.1 Hz, 1H), 3.83 (s, 3H), 1.49 (d, *J* = 7.1 Hz, 3H); ^13^C-NMR (126 MHz, DMSO-*d_6_*) δ 161.1 (d, ^1^*J*_C-F_ = 242.1 Hz), 160.8, 157.4, 141.0, 140.7 (d, ^4^*J*_C-F_ = 3.0 Hz), 140.3, 132.7, 128.1 (d, ^3^*J*_C-F_ = 8.1 Hz), 124.8, 123.6, 116.5, 115.0 (d, ^2^*J*_C-F_ = 21.2 Hz), 106.8, 55.4, 48.1, 22.12; (ESI-MS) *m/z* = 330.15 [M + H]^+^.

*(R)-N-(1-(4-fluorophenyl)ethyl)-N-methyl-5-methoxybenzothiophene-2-carboxamide* (**4b**). The compound was synthesized according to procedure D using compound 4a and methyliodide to give a pale yellow solid: yield (26%); the product was purified by CC (DCM); mp 76.8 °C; ^1^H-NMR (500 MHz, DMSO-*d_6_,* 373 Kelvin) δ 7.84 (d, *J* = 8.8 Hz, 1H), 7.64 (s, 1H), 7.43 (d, *J* = 2.4 Hz, 1H), 7.41–7.36 (m, 2H), 7.22–7.14 (m, 2H), 7.09 (dd, *J* = 8.8, 2.5 Hz, 1H), 5.72 (q, *J* = 7.0 Hz, 1H), 3.85 (s, 3H), 2.87 (s, 3H), 1.62 (d, *J* = 7.0 Hz, 3H); ^13^C-NMR (126 MHz, DMSO-*d_6_*, 373 Kelvin) δ 163.4, 161.0 (d, ^1^*J*_C-F_ = 244.0 Hz), 157.2, 139.4, 138.3, 136.01 (d, ^4^*J*_C-F_ = 3.1 Hz), 131.5, 128.3 (d, ^3^*J*_C-F_ = 8.2 Hz), 124.5, 122.5, 115.7, 114.6 (d, ^2^*J*_C-F_ = 21.4 Hz), 106.8, 55.1, 52.8, 29.6, 16.0; (ESI-MS) *m/z =* 344.18 [M + H]^+^.

*(3, 4-dihydro-1H-isoquinolin-2-yl)-(5-methoxy-benzo[b]thiophen-2-yl)-methanone* (**5a**). The compound was synthesized according to procedure C using 1,2,3,4-tetrahydro-isoquinoline to give a yellow oil: yield (27.8%); the product was purified by CC (DCM/Ethyl acetate 99:1); ^1^H-NMR (500 MHz, DMSO-*d_6_*, 373 Kelvin) δ 7.86 (d, *J* = 8.8 Hz, 1H), 7.69 (s, 1H), 7.47 (d, *J* = 2.5 Hz, 1H), 7.23–7.18 (m, 4H), 7.11 (dd, *J* = 8.8, 2.5 Hz, 1H), 4.85 (s, 2H), 3.91 (t, *J* = 6.0 Hz, 2H), 3.86 (s, 3H), 2.96 (t, *J* = 6.0 Hz, 2H); ^13^C-NMR (126 MHz, DMSO-*d_6_*, 373 Kelvin) δ 162.6, 157.2, 139.5, 138.0, 134.1, 132.7, 131.6, 127.9, 126.1, 125.8, 125.7, 124.7, 122.6, 115.8, 106.9, 55.1, 46.2, 42.9, 28.0; (ESI-MS) *m/z =* 324.21 [M + H]^+^.

*(RS)-(5-methoxy-benzo[b]thiophen-2-yl)-(2-phenyl-pyrrolidin-1-yl)-methanone* (**6a**). The compound was synthesized according to procedure C using *(RS)-*2-phenyl-pyrrolidine to give a beige solid: yield (18.4%); the product was purified by CC (DCM/Ethyl acetate 99:1); mp 142.5 °C; ^1^H-NMR (500 MHz, DMSO-*d_6_*, 373 Kelvin) δ 7.80 (d, *J* = 8.8 Hz, 1H), 7.71 (s, 1H), 7.37 (s, 1H), 7.34–7.29 (m, 2H), 7.26 (d, *J* = 7.2 Hz, 2H), 7.24–7.19 (m, 1H), 7.08 (dd, *J* = 8.8, 2.5 Hz, 1H), 5.35 (dd, *J* = 7.0, 4.0 Hz, 1H), 4.06–3.91 (m, 2H), 3.83 (s, 3H), 2.41 (dq, *J* = 12.3, 7.7 Hz, 1H), 2.01–1.93 (m, 2H), 1.90–1.84 (m, 1H); ^13^C-NMR (126 MHz, DMSO-*d_6_*, 373 Kelvin) δ 161.2, 157.1, 143.1, 139.9, 139.7, 131.9, 127.7, 126.0, 125.3, 125.0, 122.5, 116.0, 106.8, 61.4, 55.1, 48.7, 33.9, 22.9; (ESI-MS) *m/z =* 338.2 [M + H]^+^.

*N-(3-chlorobenzyl)-5-methoxybenzothiophene-2-carboxamide* (**7a**). The compound was synthesized according to procedure C using 3-chlorobenzylamine to give a white solid: yield (94%); the product was purified by CC (DCM/Petroleum ether 4:1); mp 158-160 °C; ^1^H-NMR (500 MHz, DMSO-*d_6_*) δ 9.35 (t, *J* = 6.0 Hz, 1H), 8.04 (s, 1H), 7.89 (d, *J* = 8.9 Hz, 1H), 7.44 (d, *J* = 2.4 Hz, 1H), 7.38 (dd, *J* = 10.3, 4.7 Hz, 2H), 7.32 (t, *J* = 8.7 Hz, 2H), 7.10 (dd, *J* = 8.9, 2.5 Hz, 1H), 4.49 (d, *J* = 6.0 Hz, 2H), 3.83 (s, 3H); ^13^C-NMR (126 MHz, DMSO-*d_6_*) δ 161.6, 157.4, 141.9, 140.5, 140.3, 133.0, 132.7, 130.3, 127.1, 126.8, 126.0, 124.8, 123.6, 116.8, 106.8, 55.4, 42.1; (ESI-MS) *m/z* = 332.07 [M + H]^+^.

*N-Methyl-N-(3-chlorobenzyl)-5-methoxybenzothiophene-2-carboxamide* (**7b**). The compound was synthesized according to procedure D using compound **7a** and methyl iodide to give a yellow solid: yield (38%); the product was purified by salting out, the product was dissolved in 3 ml DCM, 30 ml Petroleum ether were then added, this was followed by filtration over anhydrous MgSO_4_, 7b was recovered from the filtrate after removing the solvent under reduced pressure; mp 145-147 °C; ^1^H-NMR (500 MHz, CDCl_3_) δ 7.63 (d, *J* = 8.7 Hz, 1H), 7.38 (s, 1H), 7.28–7.22 (m, 3H), 7.14 (s, 2H), 6.99 (dd, *J* = 8.9, 1.9 Hz, 1H), 4.70 (s, 2H), 3.78 (s, 3H), 3.08 (s, 3H); ^13^C-NMR (126 MHz, CDCl_3_) δ 159.4, 158.2, 152.2, 142.1, 140.5, 140.10, 139.1, 135.2, 134.2, 133.3, 130.6, 128.3, 125.9, 123.4, 117.2, 106.6, 55.9, 30.1, 23.0; (ESI-MS) *m/z* = 346.13 [M + H]^+^; HRMS (ESI): calcd for (C_18_H_17_ClNO_2_S) 346.0663, found 346.0653 [M + H]^+^.

*N-(4-methoxybenzyl)-5-methoxybenzothiophene-2-carboxamide* (**8a**). The compound was synthesized according to procedure C using 4-methoxybenzylamine to give a white solid: yield (97.3%); the product was purified by CC (DCM); mp 176-178 °C; ^1^H-NMR (500 MHz, DMSO-*d_6_*) δ 9.22 (t, *J* = 6.0 Hz, 1H), 8.02 (s, 1H), 7.88 (d, *J* = 8.9 Hz, 1H), 7.42 (d, *J* = 2.5 Hz, 1H), 7.28–7.25 (m, 2H), 7.09 (dd, *J* = 8.9, 2.5 Hz, 1H), 6.92–6.88 (m, 2H), 4.41 (d, *J* = 6.0 Hz, 2H), 3.82 (s, 3H), 3.73 (s, 3H); ^13^C-NMR (126 MHz, DMSO-*d_6_*) δ 161.2, 158.1, 157.2, 140.9, 140.1, 132.5, 131.1, 128.6, 124.4, 123.4, 116.5, 113.6, 106.6, 55.2, 54.9, 42.0; (ESI-MS) *m/z* = 328.02 [M + H]^+^.

*N-Methyl-N-(4-methoxybenzyl)-5-methoxybenzothiophene-2-carboxamide* (**8b**). The compound was synthesized according to procedure D using compound **8a** and methyliodide to give an orange oil: yield (14.4%); the product was purified by CC (DCM); ^1^H-NMR (500 MHz, Acetone-*d_6_*) δ 7.83 (d, *J* = 8.9 Hz, 1H), 7.63 (s, 1H), 7.39 (s, 1H), 7.30 (d, *J* = 8.2 Hz, 2H), 7.08 (dd, *J* = 8.9, 2.5 Hz, 1H), 6.95 (d, *J* = 8.6 Hz, 2H), 4.75 (s, 2H), 3.85 (s, 3H), 3.80 (s, 3H), 3.13 (s, 3H); ^13^C-NMR (126 MHz, Acetone-*d_6_*) δ 160.2, 158.9, 143.0, 141.2, 129.9, 125.1, 123.9, 123.8, 117.7, 117.4, 114.9, 114.6, 107.2, 55.8, 55.5, 55.5, 43.5; (ESI-MS) *m/z* = 342.14 [M + H]^+^.

*N-(2,4-difluorobenzyl)-5-methoxybenzothiophene-2-carboxamide* (**9a**). The compound was synthesized according to procedure C using 2,4-difluorobenzylamine to give a yellowish white solid: yield (91%); the product was purified by CC (DCM/Petroleum ether 5:1); mp 166-168 °C; ^1^H-NMR (500 MHz, DMSO-*d_6_*) δ 9.27 (t, *J* = 5.8 Hz, 1H), 8.04 (d, *J* = 0.6 Hz, 1H), 7.89 (d, *J* = 8.9 Hz, 1H), 7.48–7.44 (m, 1H), 7.43 (d, *J* = 2.4 Hz, 1H), 7.24 (ddd, *J* = 10.5, 9.4, 2.6 Hz, 1H), 7.11–7.06 (m, 2H), 4.49 (d, *J* = 5.7 Hz, 2H), 3.83 (s, 3H); ^13^C-NMR (126 MHz, DMSO-*d_6_*) δ 161.6 (dd, ^1^*J*_C−F_, ^3^*J*_C−F_ = 176.8, 12.3 Hz), 161.5, 159.6 (dd, ^1^*J*_C−F_, ^3^*J*_C−F_ = 179.0, 12.3 Hz), 157.2, 140.3, 140.1, 132.6, 130.9 (dd, ^3^*J*_C−F_, ^3^*J*_C−F_ = 9.9, 5.9 Hz), 124.7, 123.4, 122.0 (dd, ^2^*J*_C−F_, ^4^*J*_C−F_ = 15.0, 3.6 Hz), 116.6, 111.2 (dd, ^2^*J*_C−F_, ^4^*J*_C−F_ = 21.1, 3.6 Hz), 106.6, 103.5 (t, ^2^*J*_C−F_ = 25.8 Hz), 55.2, 36.0 (d, ^3^*J*_C−F_ = 3.8 Hz); (ESI-MS) *m/z* = 334.08 [M + H]^+^.

*N-Methyl-N-(2,4-difluorobenzyl)-5-methoxybenzothiophene-2-carboxamide* (**9b**). The compound was synthesized according to procedure D using compound **9a** and methyliodide to give a yellow solid: yield (40.6%); the product was purified by CC (DCM/Petroleum ether 4:1); mp 116-118 °C; ^1^H-NMR(500 MHz, DMSO-*d_6_*) δ 7.88 (d, *J* = 8.9 Hz, 1H), 7.75 (s, 1H), 7.43 (p, *J* = 8.4 Hz, 2H), 7.29 (ddd, *J* = 10.6, 9.4, 2.6 Hz, 1H), 7.15–7.11 (m, 1H), 7.09 (dd, *J* = 8.9, 2.5 Hz, 1H), 4.77 (s, 2H), 3.81 (s, 3H), 3.19 (s, 3H); ^13^C NMR(126 MHz, DMSO-*d_6_*) δ 162.0 (dd, ^1^*J*_C−F_, ^3^*J*_C−F_ = 160.8, 12.0 Hz), 160.0 (dd, ^1^*J*_C−F_, ^3^*J*_C−F_ = 162.4, 12.2 Hz), 157.4, 139.9, 138.3, 131.9, 131.0 (dd, ^3^*J*_C−F_, ^3^*J*_C−F_ = 11.3, 2.0 Hz), 126.1, 124.9, 123.2, 120.2 (dd, ^2^*J*_C−F_, ^4^*J*_C−F_ = 15.4, 3.2 Hz), 116.6, 111.70 (dd, ^2^*J*_C−F_, ^4^*J*_C−F_ = 21.2, 3.3 Hz), 106.7, 104.1 (t, ^2^*J*_C−F_ = 25.4 Hz), 55.3, 45.1 (d, ^3^*J*_C−F_ = 7.6 Hz), 29.2; (ESI-MS) *m/z* = 348.06 [M + H]^+^.

*N-(3,5-difluorobenzyl)-5-methoxybenzothiophene-2-carboxamide* (**10a**). The compound was synthesized according to procedure C using 3,5-difluorobenzylamine to give a white solid: yield (88.1%); the product was purified by CC (DCM/Petroleum ether 4:1.5); mp 170-172 °C; ^1^H NMR(500 MHz, DMSO-*d_6_*) δ 9.37 (t, *J* = 6.0 Hz, 1H), 8.05 (d, *J* = 0.5 Hz, 1H), 7.90 (d, *J* = 8.9 Hz, 1H), 7.45 (d, *J* = 2.5 Hz, 1H), 7.16–7.09 (m, 2H), 7.08–7.03 (m, 2H), 4.51 (d, *J* = 6.0 Hz, 2H), 3.83 (s, 3H); ^13^C-NMR (126 MHz, DMSO-*d_6_*) δ 162.4 (dd, ^1^*J*_C−F_, ^3^*J*_C−F_ = 246.2, 13.1 Hz), 161.8, 157.4, 144.1 (t, ^3^*J*_C−F_ = 8.8 Hz), 140.3, 140.2, 132.7, 125.0, 123.6, 116.8, 110.2 (dd, ^2^*J*_C−F_, ^4^*J*_C−F_ = 19.5, 5.8 Hz), 106.8, 102.3 (t, ^2^*J*_C−F_ = 25.8 Hz), 55.4, 42.0; (ESI-MS) *m/z* = 334.09 [M + H]^+^.

*N-Methyl-N-(3,5-difluorobenzyl)-5-methoxybenzothiophene-2-carboxamide* (**10b**). The compound was synthesized according to procedure D using compound 10a and methyliodide to give a white solid: yield (72.3%), the product was purified by CC (DCM/Petroleum ether 4:1); mp 107–109 °C; ^1^H-NMR (500 MHz, DMSO-*d_6_*) δ 7.89 (d, *J* = 8.9 Hz, 1H), 7.43 (s, 1H), 7.18 (t, *J* = 9.4 Hz, 1H), 7.09 (d, *J* = 7.8 Hz, 1H), 7.04 (d, *J* = 6.7 Hz, 2H), 4.75 (s, 2H), 3.81 (s, 3H), 3.25 (s, 3H); ^13^C NMR(126 MHz, DMSO-*d_6_*) δ 165.0, 162.0 (dd, ^1^*J*_C−F_, ^3^*J*_C−F_ = 247.5, 12.6 Hz), 161.7, 157.6, 139.9, 138.9, 133.9, 129.2, 123.8, 118.0, 112.0 (dd, ^2^*J*_C−F_, ^4^*J*_C−F_ = 27.0, 13.8 Hz), 107.8 (t, ^2^*J*_C−F_ = 25.8 Hz), 107.2, 55.4, 54.9, 45.8; (ESI-MS) *m/z* = 348.1 [M + H]^+^; HRMS (ESI): calcd for (C_18_H_16_F_2_NO_2_S) 348.0864, found 348.0855 (M+ H)^+^.

*N-(2,5-difluorobenzyl)-5-methoxybenzothiophene-2-carboxamide* (**11a**). The compound was synthesized according to procedure C using 2,5-difluorobenzylamine to give a white solid: yield (89.6%); the compound was purified by CC (DCM/Petroleum ether 4:1); mp 143-145 °C; ^1^H-NMR (500 MHz, DMSO-*d_6_*) δ 9.30 (t, *J* = 5.8 Hz, 1H), 8.06 (d, *J* = 0.6 Hz, 1H), 7.89 (d, *J* = 8.9 Hz, 1H), 7.44 (d, *J* = 2.5 Hz, 1H), 7.27 (td, *J* = 9.2, 4.5 Hz, 1H), 7.23–7.14 (m, 2H), 7.11 (dd, *J* = 8.9, 2.5 Hz, 1H), 4.51 (d, *J* = 5.8 Hz, 2H), 3.83 (s, 3H); ^13^C-NMR (126 MHz, DMSO-*d_6_*) δ 161.7, 158.1 (dd, ^1^*J*_C−F_, ^4^*J*_C−F_ = 240.0, 2.1 Hz), 157.4, 156.1 (dd, ^1^*J*_C−F_, ^4^*J*_C−F_ = 240.7, 2.0 Hz), 140.3, 140.2, 132.7, 128.0 (dd, ^2^*J*_C−F_, ^3^*J*_C−F_ = 17.6, 7.6 Hz), 125.0, 123.6, 116.8, 116.7 (dd, ^2^*J*_C−F_, ^3^*J*_C−F_ = 24.2, 9.0 Hz), 115.8 (dd, ^2^*J*_C−F_, ^3^*J*_C−F_ = 24.8, 4.8 Hz), 115.3 (dd, ^2^*J*_C−F_, ^3^*J*_C−F_ = 24.0, 8.6 Hz), 106.8, 55.4, 36.5; (ESI-MS) *m/z* = 334.07 [M + H]^+^.

*N-Methyl-N-(2,5-difluorobenzyl)-5-methoxybenzothiophene-2-carboxamide* (**11b**). The compound was synthesized according to procedure D using compound 11a and methyliodide to give a yellowish white solid: yield (49.3%); the compound was purified by CC (DCM/Petroleum ether 3:1); mp 91-93 °C; ^1^H-NMR (500 MHz, DMSO-*d_6_*) δ 7.88 (d, *J* = 8.9 Hz, 1H), 7.81 (s, 1H), 7.44 (d, *J* = 2.2 Hz, 1H), 7.30 (td, *J* = 9.2, 4.5 Hz, 1H), 7.20 (tdd, *J* = 8.9, 6.6, 3.2 Hz, 2H), 7.09 (dd, *J* = 8.8, 2.4 Hz, 1H), 4.78 (s, 2H), 3.81 (s, 3H), 3.24 (s, 3H); ^13^C-NMR (126 MHz, DMSO-*d_6_*) δ 163.8, 158.3 (d, ^1^*J*_C−F_ = 240.2 Hz), 157.4, 156.4 (d, ^1^*J*_C−F_ = 231.8 Hz), 139.9, 138.3 (dd, ^2^*J*_C−F_, ^3^*J*_C−F_ = 7.2, 4.5 Hz), 131.9, 126.0 (dd, ^2^*J*_C−F_, ^3^*J*_C−F_ = 17.3, 7.5 Hz), 123.2, 122.8, 117.0 (dd, ^2^*J*_C−F_, ^3^*J*_C−F_ = 24.8, 8.4 Hz), 116.6, 115.7 (dd, ^2^*J*_C−F_, ^3^*J*_C−F_ = 24.4, 7.0 Hz), 106.7, 55.3, 45.7, 38.6; (ESI-MS) *m/z* = 348.05 [M + H]^+^; HRMS (ESI): calcd for (C_18_H_16_F_2_NO_2_S) 348.0864, found 348.0855 [M + H]^+^.

*N-(3-fluoro-4-methoxybenzyl)-5-methoxybenzothiophene-2-carboxamide* (**12a**). The compound was synthesized according to procedure C using 3-fluoro-4-methoxybenzylamine to give a white solid: yield (86.4%); the compound was purified by CC (DCM); mp 170–172 °C; ^1^H-NMR (500 MHz, DMSO-*d_6_*) δ 9.26 (t, *J* = 6.0 Hz, 1H), 8.02 (d, *J* = 0.6 Hz, 1H), 7.89 (dt, *J* = 8.9, 0.5 Hz, 1H), 7.43 (d, *J* = 2.4 Hz, 1H), 7.19–7.11 (m, 3H), 7.10 (dd, *J* = 8.9, 2.5 Hz, 1H), 4.41 (d, *J* = 6.0 Hz, 2H), 3.83 (s, 3H), 3.81 (s, 3H); ^13^C-NMR (126 MHz, DMSO-*d_6_*) δ 161.5, 157.4, 151.3 (d, ^1^*J*_C−F_ = 243.7 Hz), 146.0 (d, ^2^*J*_C−F_ = 10.6 Hz), 140.7, 140.3, 132.7, 132.2 (d, ^3^*J*_C−F_ = 5.8 Hz), 124.7, 123.6, 123.6, 116.67, 115.0 (d, ^2^*J*_C−F_ = 18.2 Hz), 113.7 (d, ^3^*J*_C−F_ = 1.6 Hz), 106.7, 56.0, 55.4, 41.8; (ESI-MS) *m/z* = 346.07 [M + H]^+^.

*N-Methyl-N-(3-fluoro-4-methoxybenzyl)-5-methoxybenzothiophene-2-carboxamide* (**12b**). The compound was synthesized according to procedure D using compound **12a** and methyliodide to give a white solid: yield (37.3%); the compound was purified by CC (DCM/Petroleum ether 3:1); mp 107–109 °C); ^1^H-NMR (500 MHz, DMSO-*d_6_*) δ 7.88 (d, *J* = 8.9 Hz, 1H), 7.75 (s, 1H), 7.42 (d, *J* = 1.7 Hz, 1H), 7.18 (t, *J* = 8.6 Hz, 2H), 7.09 (dd, *J* = 8.8, 2.3 Hz, 2H), 4.67 (s, 2H), 3.83 (s, 3H), 3.80 (s, 3H), 3.15 (s, 3H); ^13^C-NMR (126 MHz, DMSO-*d_6_*) δ 163.6, 157.4, 151.4 (d, ^1^*J*_C−F_ = 244.6 Hz), 146.4 (d, ^2^*J*_C−F_ = 10.5 Hz), 140.0, 138.4, 131.8, 129.9 (d, ^3^*J*_C−F_ = 4.7 Hz), 126.1, 123.8 (d, ^3^*J*_C−F_ = 2.3 Hz), 123.2, 116.5, 115.3 (d, ^2^*J*_C−F_ = 12.7 Hz), 114.0, 106.6, 56.0, 55.3, 50.4, 36.9; (ESI-MS) *m/z* = 360.11 [M + H]^+^; HRMS (ESI): calcd for (C_19_H_19_FNO_3_S) 360.1064, found 360.1054 (M+ H)^+^.

*N-(3-fluoro-2-methoxybenzyl)-5-methoxybenzothiophene-2-carboxamide* (**13a**). The compound was synthesized according to procedure C using 3-fluoro-2-methoxybenzylamine to give a white solid: yield (93.6%); the compound was purified by CC (DCM/Petroleum ether 4:1); mp 136-138 °C); ^1^H-NMR (500 MHz, DMSO-*d_6_*) δ 9.21 (t, *J* = 5.8 Hz, 1H), 8.06 (d, *J* = 0.5 Hz, 1H), 7.89 (d, *J* = 8.9 Hz, 1H), 7.43 (d, *J* = 2.4 Hz, 1H), 7.18 (ddd, *J* = 11.6, 8.0, 1.8 Hz, 1H), 7.15–7.12 (m, 1H), 7.12–7.06 (m, 2H), 4.52 (d, *J* = 5.8 Hz, 2H), 3.90 (d, *J* = 1.5 Hz, 3H), 3.83 (s, 3H); ^13^C-NMR (126 MHz, DMSO-*d_6_*) δ 161.6, 157.4, 154.9 (d, ^1^*J*_C−F_ = 245.0 Hz), 144.8 (d, ^2^*J*_C−F_ = 10.9 Hz), 140.7, 140.3, 133.8 (d, ^4^*J*_C−F_ = 1.8 Hz), 132.7, 124.7, 124.0 (d, ^3^*J*_C−F_ = 2.8 Hz), 123.9 (d, ^3^*J*_C−F_ = 8.0 Hz), 123.6, 116.7, 115.5 (d, ^2^*J*_C−F_ = 19.0 Hz), 106.8, 61.2 (d, ^4^*J*_C−F_ = 5.2 Hz), 55.4, 37.4; (ESI-MS) *m/z* = 346.06 [M + H]^+^.

*N-Methyl-N-(3-fluoro-2-methoxybenzyl)-5-methoxybenzothiophene-2-carboxamide* (**13b**). The compound was synthesized according to procedure D using compound 13a and methyliodide to give a white oil: yield (75%); the compound was purified by CC (DCM/Petroleum ether 4:1); ^1^H-NMR (500 MHz, DMSO-*d_6_*) δ 7.88 (d, *J* = 8.9 Hz, 1H), 7.79 (s, 1H), 7.43 (s, 1H), 7.26–7.20 (m, 1H), 7.13 (dt, *J* = 12.2, 6.0 Hz, 1H), 7.08 (dd, *J* = 10.1, 4.4 Hz, 2H), 4.78 (s, 2H), 3.84 (s, 3H), 3.81 (s, 3H), 3.22 (s, 3H); ^13^C-NMR (126 MHz, DMSO-*d_6_*) δ 163.6, 157.4, 154.9 (d, ^1^*J*_C−F_ = 245.1 Hz), 145.1 (d, ^2^*J*_C−F_ = 14.5 Hz), 139.9, 138.6, 131.8, 131.6, 126.0, 124.1 (d, ^3^*J*_C−F_ = 7.9 Hz), 123.8 (d, ^3^*J*_C−F_ = 6.4 Hz), 123.2, 116.5, 116.1 (d, ^2^*J*_C−F_ = 14.3 Hz), 106.6, 61.1 (d, ^4^*J*_C−F_ = 2.7 Hz), 55.3, 49.9, 37.5; (ESI-MS) *m/z* = 360.14 [M + H]^+^.

*N-(5-fluoro-2-methoxybenzyl)-5-methoxybenzothiophene-2-carboxamide* (**14a**). The compound was synthesized according to procedure C using 5-fluoro-2-methoxybenzylamine to give a white solid: yield (82.5%); the compound was purified by CC (DCM/Petroleum ether 4:1); mp 172-174 °C; ^1^H-NMR (500 MHz, DMSO-*d_6_*) δ 9.19 (t, *J* = 5.8 Hz, 1H), 8.09 (s, 1H), 7.90 (d, *J* = 8.9 Hz, 1H), 7.45 (d, *J* = 2.4 Hz, 1H), 7.11 (dd, *J* = 8.9, 2.5 Hz, 1H), 7.07 (dd, *J* = 8.5, 3.1 Hz, 1H), 7.01 (dd, *J* = 9.0, 4.0 Hz, 2H), 4.44 (d, *J* = 5.8 Hz, 2H), 3.83 (s, 3H), 3.82 (s, 3H); ^13^C-NMR (126 MHz, DMSO-*d_6_*) δ 161.8, 157.4, 156.3 (d, ^1^*J*_C−F_ = 235.5 Hz), 152.8, 140.6, 140.3, 132.7, 128.7 (d, ^3^*J*_C−F_ = 6.9 Hz), 124.8, 123.6, 116.7, 114.2 (d, ^2^*J*_C−F_ = 24.3 Hz), 113.7 (d, ^2^*J*_C−F_ = 22.6 Hz), 111.8 (d, ^3^*J*_C−F_ = 8.2 Hz), 106.8, 56.0, 55.4, 37.5; (ESI-MS) *m/z =* 346.07 [M + H]^+^.

*N-Methyl-N-(5-fluoro-2-methoxybenzyl)-5-methoxybenzothiophene-2-carboxamide* (**14b**). The compound was synthesized according to procedure D using compound 14a and methyliodide to give a white oil: yield (75%); the compound was purified by CC (DCM/Petroleum ether 4:1); ^1^H-NMR (500 MHz, DMSO-*d_6_*) δ 7.88 (d, *J* = 8.8 Hz, 1H), 7.51 (s, 1H), 7.43 (s, 1H), 7.14–7.06 (m, 2H), 7.04 (dd, *J* = 9.0, 4.5 Hz, 2H), 4.67 (s, 2H), 3.81 (s, 6H), 3.26 (s, 3H); ^13^C-NMR (126 MHz, DMSO-*d_6_*) δ 157.4, 156.4 (d, ^1^*J*_C−F_ = 236.1 Hz), 153.3, 139.9, 138.6, 131.8, 130.6, 126.5 (d, ^3^*J*_C−F_ = 5.8 Hz), 126.2, 123.2, 116.5, 114.40 (d, ^2^*J*_C−F_ = 24.0 Hz), 114.1 (d, ^3^*J*_C−F_ = 4.8 Hz), 112.1 (d, ^2^*J*_C−F_ = 8.1 Hz), 106.6, 55.9, 55.3, 54.9, 44.9; (ESI-MS) *m/z* = 360.1 [M + H]^+^.

*N-(3-fluoro-5-methybenzyl)-5-methoxybenzothiophene-2-carboxamide* (**15a**). The compound was synthesized according to procedure C using 3-fluoro-5-methylbenzylamine to give a white solid: yield (93.9%); the compound was purified by CC (DCM/Petroleum ether 4:1); mp; ^1^H-NMR (500 MHz, DMSO-*d_6_*) δ 9.30 (t, *J* = 6.0 Hz, 1H), 8.04 (d, *J* = 0.5 Hz, 1H), 7.89 (d, *J* = 8.9 Hz, 1H), 7.44 (d, *J* = 2.4 Hz, 1H), 7.10 (dd, *J* = 8.9, 2.5 Hz, 1H), 7.00–6.98 (m, 1H), 6.95–6.90 (m, 2H), 4.46 (d, *J* = 6.0 Hz, 2H), 3.83 (s, 3H), 2.30 (s, 3H); ^13^C-NMR (126 MHz, DMSO-*d_6_*) δ 162.2 (d, ^1^*J*_C−F_ = 242.8 Hz), 161.6, 157.4, 141.9 (d, ^3^*J*_C−F_ = 7.7 Hz), 140.6, 140.3, 140.3, 132.7, 124.8, 123.9 (d, ^3^*J*_C−F_ = 2.2 Hz), 123.6, 116.7, 114.1 (d, ^2^*J*_C−F_ = 20.9 Hz), 111.0 (d, ^2^*J*_C−F_ = 21.7 Hz), 106.7, 55.4, 42.2 (d, ^4^*J*_C−F_ = 1.6 Hz), 20.8 (d, ^4^*J*_C−F_ = 1.7 Hz); (ESI-MS) *m/z* = 330.07 [M + H]^+^.

*N-Methyl-N-(3-fluoro-5-methybenzyl)-5-methoxybenzothiophene-2-carboxamide* (**15b**). The compound was synthesized according to procedure D using compound 15a and methyliodide to give a white oil: yield (57.5%); the compound was purified by CC (DCM/Petroleum ether 4:1); ^1^H-NMR (500 MHz, CDCl_3_) δ 7.68 (d, *J* = 8.8 Hz, 1H), 7.45 (s, 1H), 7.18 (s, 1H), 7.03 (dd, *J* = 8.9, 2.2 Hz, 1H), 6.89 (d, *J* = 14.2 Hz, 1H), 6.81 (d, *J* = 9.1 Hz, 2H), 4.72 (s, 2H), 3.83 (s, 3H), 3.12 (s, 3H), 2.34 (s, 3H); ^13^C-NMR (126 MHz, CDCl_3_) δ 163.4 (d, ^1^*J*_C−F_ = 246.7 Hz), 161.6, 158.0, 141.3, 140.0, 139.1 (d, ^3^*J*_C−F_ = 7.5 Hz), 138.4 (d, ^3^*J*_C−F_ = 5.6 Hz), 133.2, 124.7 (d, ^4^*J*_C−F_ = 3.8 Hz), 123.5, 123.3, 116.9, 115.5 (d, ^2^*J*_C−F_ = 21.0 Hz), 111.4 (d, ^2^*J*_C−F_ = 23.7 Hz), 106.4, 55.8, 29.9, 26.0 (d, ^4^*J*_C−F_ = 4.1 Hz), 21.6 (d, ^4^*J*_C−F_ = 1.5 Hz); (ESI-MS) *m/z* = 344.12 [M + H]^+^; HRMS (ESI): calcd for (C_19_H_19_FNO_2_S) 344.1115, found 344.1105 (M+ H)^+^.

### 3.2. Biological Assays

#### 3.2.1. Protein Kinases and Inhibition Assays

Active human Clk1 and Clk2 were purchased from Life Technologies (Catalog No. PV3315 and PV4279, respectively). RS repeat substrate peptide for Clk1 (GRSRSRSRSRSRSRSR) was custom synthesized at the Department of Medical Biochemistry and Molecular Biology, Saarland University, Homburg, Germany. Kinase Inhibition assays for Clk1 and Clk2 were performed as described previously, in the presence of 15 μM ATP [20]. The calculated IC_50_ values are representative of at least two independent determinations performed in duplicates. The larger panel of kinases shown in Table 4 was screened by the SelectScreen Kinase Profiling Service, Thermo Fisher Scientific, Paisley, UK.

#### 3.2.2. Antiproliferative Assay

The T24 bladder carcinoma cell line was cultured in Dulbecco’s Modified Eagles Medium (DMEM, Sigma-Aldrich). The DMEM medium was supplemented with 1% pen/strep solution (Invitrogen) and 5% fetal calf serum (FCS, Sigma-Aldrich, St. Louis, MO, USA). T24 cells were seeded in a 96-well plate (5000 cells per well) already containing DMSO as a control (0.2% final concentration) or the test compounds dissolved in DMSO. The cells were grown for five days at 37 °C in a humidified incubator containing 5% CO_2_, without further change of medium, before the detection was carried out as described in [30].

### 3.3. Molecular Modeling

All procedures were performed using the Molecular Operating Environment (MOE) software package (version 2016, Chemical Computing Group, Montreal, Canada). For the docking simulations, PDB entry 1Z57 (Clk1 co-crystallized with hymenialdisine) [18] was used. Molecular docking simulations with the **3a (*S*), 5a, 6a, 7b, 9b** and **10b** ligands were performed using the MMFF94x force field, and the Amber10:EHT force field was used for compound **7b**; the placement method was set to “Alpha PMI” (number of return poses set to 2000), and refinement was set to “induced fit” to enable free side chain movements. Using these settings, docking runs were performed in two different ways: (i) the binding pocket was defined by selecting nearby residues to the co-crystallized ligand (hymenialdisine); (ii) after an initial run using condition i, the MOE LigX routine was applied (settings: receptor strength = 5, ligand strength = 5000) for energy minimization, and a pharmacophore was defined based on a suitable pose by selecting both the methoxy oxygen close to the NH of the leucine and the carbonyl oxygen near the conserved lysine as acceptor points (this binding orientation prevailed among the poses with the compound docked deep in the pocket). In the pharmacophore definition window, the radius of the pharmacophore points was raised to 1.2 Å. The subsequent docking was performed using the pharmacophore-supported placement. The number of retained poses was set to 500 each time. Only the poses with the top 10 scoring values were further evaluated for plausibility. The final selected poses were optimized again using the LigX routine, using the same settings as described above.

## 4. Conclusions

In the present study, various strategies were employed to increase the potency and/or selectivity of our previously reported *N*-methylbenzothiophene-2-carboxamides as inhibitors of Clk1 [2]. While keeping the methylated amide function, introduction of the *m*-chlorobenzyl as well as di-substituted benzyl extensions led to an enhanced selectivity for Clk1 over Clk2 without loss of the high potency toward Clk1, when compared with the former flagship compound **1b**. To the best of our knowledge, **10b**, **7b**, **12b** and **15b** show the highest selectivity for Clk1/4 over Clk2, as reported previously for Clk1 inhibitors. In addition, compound **10b** was more potent in cells than **1b,** exhibiting the highest anti-proliferative activity against T24 bladder carcinoma cells. The overall selectivity profile of **10b** against the most common off-targets for Clk1 inhibitors was comparable to that of **1b**, with the important difference that **10b** was four times more selective for Clk2 inhibition. The latter could be considered a significant achievement, because more selective Clk inhibitors might reduce the side effects that can be expected following the application of pan Clk inhibitors; in such cases, the alternative splicing in all tissues might be strongly compromised due to the simultaneous blocking of all Clk activities. In terms of a targeted tumor therapy, it seems more appropriate to inhibit only the Clk isoforms that are overexpressed in the respective tumor type. An analysis of the Oncomine mRNA expression database (oncomine.org) revealed that for dual Clk1/Clk4 inhibitors, the most promising oncologic indications are glioblastoma and lymphoma, in which mRNA overexpression compared to healthy tissue was detected for both isoforms.

## Figures and Tables

**Table 1:**
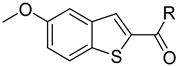
Clk1/Clk2 inhibitory activity of the mono-substituted benzyl derivatives.

Cpd. No.	R	Clk1% Inhibition at 100 nM *^a^*	IC_50_ (nM) *^b^*	Clk2% Inhibition at 250 nM *^a^*	IC_50_ (nM) *^b^*	Cpd. No.	R	Clk1% Inhibition at 100 nM *^a^*	IC_50_ (nM)*^b^*	Clk2 % Inhibition at 250 nM *^a^*	IC_50_ (nM) *^b^*
**1a** *^c^*	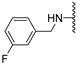	33.9	n.d.	29.8	n.d.	**4a**	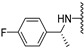	54	114 ± 5.4	n.i.	n.d.
**1b** *^c^*	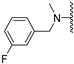	80	6.9 ± 0.12	--	19.1 ± 0.5	**4b**	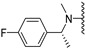	n.i.	n.d.	n.i.	n.d.
**1c**	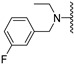	50.2	100	42.4	n.d.	**5a**	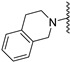	n.i.	n.d.	n.i.	n.d.
**1d**	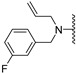	n.i.	n.d.	n.i.	n.d.	**6a**	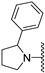	n.i.	n.d.	n.i.	n.d.
**2a** *^c^*	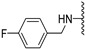	23.5	n.d.	n.d.	n.d.	**7a**	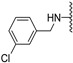	34.7	n.d.	55	n.d.
**2b** *^c^*	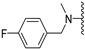	28.7	n.d.	n.d.	n.d.	**7b**	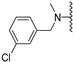	88.5	6.2 ± 0.1	73.4	104 ± 4.2
**3a**	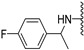	28.7	n.d.	n.i.	n.d.	**8a**	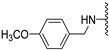	n.i.	n.d.	n.i.	n.d.
**3b**	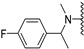	n.i.	n.d.	n.i.	n.d.	**8b**	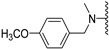	45.5	n.d.	23.7	n.d.

*^a^* Data shown are the mean of at least two independent experiments, SD ≤ 10%. *^b^* IC50 ± SD, n.d., not determined; n.i., no inhibition. *^c^* Compounds **1a**, **1b**, **2a** and **2b** were reported previously in Ref. [2].

**Table 2:**
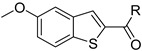
Clk1/Clk2 inhibitory activity of the di-substituted benzyl derivatives.

Cpd. No.	R	Clk1% Inhibition at 100 nM *^a^*	IC50 (nM) *^b^*	Clk2% Inhibition at 250 nM *^a^*	IC50 (nM) *^b^*	Cpd. No.	R	Clk1% Inhibition at 100 nM *^a^*	IC50 (nM) *^b^*	Clk2% Inhibition at 250 nM *^a^*	IC50 (nM) *^b^*
**9a**	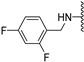	n.i.	n.d.	25.8	n.d.	**12b**	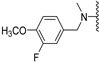	82.8	16.8 ± 1.2	39.7	n.d.
**9b**	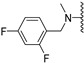	8.5	n.d.	16.9	n.d.	**13a**	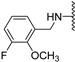	12.5	n.d.	26.9	n.d.
**10a**	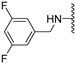	62.2	53.5 ± 3.6	30	n.d.	**13b**	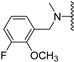	17.6	n.d.	17	n.d.
**10b**	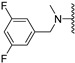	88.8	12.7 ± 0.9	74.7	125 ± 7	**14a**	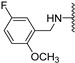	43.2	n.d.	24.5	n.d.
**11a**	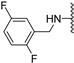	31.8	n.d.	3	n.d.	**14b**	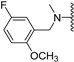	49.2	n.d.	44	n.d.
**11b**	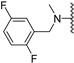	71.2	33.6 ± 1.8	52.7	103 ± 5	**15a**	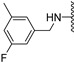	42	n.d.	20	n.d.
**12a**	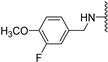	n.i.	n.d.	27	n.d.	**15b**	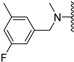	87.8	10.9 ± 0.6	72.5	167 ± 9

*^a^* Data shown are the mean of at least two independent experiments, SD ≤ 10%. *^b^* IC50 ± SD, n.d., not determined; n.i., no inhibition.

**Table 3:** Growth inhibition of the most potent Clk1 inhibitors against the T24 cancer cell line.

Cpd. No.	GI_50_ (μM) *^a^*	Clk1 Inhibition IC_50_ (nM)	Selectivity Factor for Clk1 over Clk2
**1b**	0.63	6.9	2.7
**7b**	8.3	6.2	16
**10b**	0.43	12.7	10
**12b**	12.12	16.8	>15
**15b**	7.92	10.9	15

*^a^* Data shown are the mean of two independent experiments, S.D. ≤ 12%.

**Table 4:** Selectivity profiling of compound **10b**.

10b	
Kinase	% Inhibition at 1 μM *^a^*
CDK5/p25	3
DYRK1A *^b^*	75% @ 0.75 µM IC_50_ = 355 ± 20 nM
DYRK1B	70
DYRK2	19
DYRK3	3
Clk1	80
Clk3	48
Clk4	100
PIM1	6
CK1 γ2	2
CK2 α1	5
Haspin	0
MLCK2	3
STK17A (DRAK1)	25
SRPK1	4
SRPK2	3

*^a^* Screenings were performed as a service at ThermoFisher Scientific using ATP concentrations at the K_m_ value. Data represent mean values of duplicates that differed by less than 7%. *^b^* Dyrk1a screening and IC_50_ determination were performed in house at an ATP concentration of 15 µM; IC_50_ value ± SD.

## Data Availability

Data is contained within the article and in the Supplementary Materials.

## Supplementary Materials

The following are available online, Figure S1: Molecular docking of compound **5a** in the ATP binding pocket of Clk1, Figure S2: Molecular docking of compounds **3a (*S*)** and **6a** in the ATP binding pocket of Clk1, Figure S3: Molecular docking of compound **9b** in the ATP binding pocket of Clk1.

## Sample Availability

Samples of the compounds are available from the authors.

## References

[1] Iwai K., Yaguchi M., Nishimura K., Yamamoto Y., Tamura T., Nakata D., Dairiki R., Kawakita Y., Mizojiri R., Ito Y. (2018). Anti-tumor efficacy of a novel CLK inhibitor via targeting RNA splicing and MYC-dependent vulnerability. EMBO Mol. Med..

[2] ElHady A.K., Abdel-Halim M., Abadi A.H., Engel M. (2017). Development of Selective Clk1 and-4 Inhibitors for Cellular Depletion of Cancer-Relevant Proteins. J. Med. Chem..

[3] Corkery D.P., Holly A.C., Lahsaee S., Dellaire G. (2015). Connecting the speckles: Splicing kinases and their role in tumorigenesis and treatment response. Nucleus.

[4] Capra M., Nuciforo P.G., Confalonieri S., Quarto M., Bianchi M., Nebuloni M., Boldorini R., Pallotti F., Viale G., Gishizky M.L. (2006). Frequent alterations in the expression of serine/threonine kinases in human cancers. Cancer Res..

[5] Duchon A., Herault Y. (2016). DYRK1A, a dosage-sensitive gene involved in neurodevelopmental disorders, is a target for drug development in Down syndrome. Front. Behav. Neurosci..

[6] Kuhn C., Frank D., Will R., Jaschinski C., Frauen R., Katus H.A., Frey N. (2009). DYRK1A is a novel negative regulator of cardiomyocyte hypertrophy. J. Biol. Chem..

[7] Soppa U., Schumacher J., Florencio Ortiz V., Pasqualon T., Tejedor F., Becker W. (2014). The Down syndrome-related protein kinase DYRK1A phosphorylates p27Kip1 and Cyclin D1 and induces cell cycle exit and neuronal differentiation. Cell Cycle.

[8] Yoshida T., Kim J.H., Carver K., Su Y., Weremowicz S., Mulvey L., Yamamoto S., Brennan C., Mei S., Long H. (2015). CLK2 is an oncogenic kinase and splicing regulator in breast cancer. Cancer Res..

[9] Loidreau Y., Marchand P., Dubouilh-Benard C., Nourrisson M.-R., Duflos M., Loaëc N., Meijer L., Besson T. (2013). Synthesis and biological evaluation of N-aryl-7-methoxybenzo [b] furo [3,2-d] pyrimidin-4-amines and their N-arylbenzo [b] thieno [3,2-d] pyrimidin-4-amine analogues as dual inhibitors of CLK1 and DYRK1A kinases. Eur. J. Med. Chem..

[10] Esvan Y.J., Zeinyeh W., Boibessot T., Nauton L., Théry V., Knapp S., Chaikuad A., Loaëc N., Meijer L., Anizon F. (2016). Discovery of pyrido [3,4-g] quinazoline derivatives as CMGC family protein kinase inhibitors: Design, synthesis, inhibitory potency and X-ray co–crystal structure. Eur. J. Med. Chem..

[11] Zeinyeh W., Esvan Y.J., Nauton L., Loaëc N., Meijer L., Théry V., Anizon F., Giraud F., Moreau P. (2016). Synthesis and preliminary in vitro kinase inhibition evaluation of new diversely substituted pyrido [3,4-g] quinazoline derivatives. Bioorg. Med. Chem. Lett..

[12] Tazarki H., Zeinyeh W., Esvan Y.J., Knapp S., Chatterjee D., Schröder M., Joerger A.C., Khiari J., Josselin B., Baratte B. (2019). New pyrido [3,4-g] quinazoline derivatives as CLK1 and DYRK1A inhibitors: Synthesis, biological evaluation and binding mode analysis. Eur. J. Med. Chem..

[13] Schmitt C., Miralinaghi P., Mariano M., Hartmann R.W., Engel M. (2014). Hydroxybenzothiophene ketones are efficient pre-mRNA splicing modulators due to dual inhibition of Dyrk1A and Clk1/4. ACS Med. Chem. Lett..

[14] Bendjeddou L.Z., Loaëc N., Villiers B., Prina E., Späth G.F., Galons H., Meijer L., Oumata N. (2017). Exploration of the imidazo [1,2-b] pyridazine scaffold as a protein kinase inhibitor. Eur. J. Med. Chem..

[15] Ferrari S.M., Centanni M., Virili C., Miccoli M., Ferrari P., Ruffilli I., Ragusa F., Antonelli A., Fallahi P. (2019). Sunitinib in the treatment of thyroid cancer. Curr. Med. Chem..

[16] Murár M., Dobiaš J., Šramel P., Addová G., Hanquet G., Boháč A. (2017). Novel CLK1 inhibitors based on N-aryloxazol-2-amine skeleton-a possible way to dual VEGFR2 TK/CLK ligands. Eur. J. Med. Chem..

[17] Araki S., Dairiki R., Nakayama Y., Murai A., Miyashita R., Iwatani M., Nomura T., Nakanishi O. (2015). Inhibitors of CLK protein kinases suppress cell growth and induce apoptosis by modulating pre-mRNA splicing. PLoS ONE.

[18] Bullock A.N., Das S., Debreczeni J.É., Rellos P., Fedorov O., Niesen F.H., Guo K., Papagrigoriou E., Amos A.L., Cho S. (2009). Kinase domain insertions define distinct roles of CLK kinases in SR protein phosphorylation. Structure.

[19] Prak K., Kriston-Vizi J., Chan A.E., Luft C., Costa J.R., Pengo N., Ketteler R. (2016). Benzobisthiazoles represent a novel scaffold for kinase inhibitors of CLK family members. Biochemistry.

[20] Schmitt C., Kail D., Mariano M., Empting M., Weber N., Paul T., Hartmann R.W., Engel M. (2014). Design and synthesis of a library of lead-like 2, 4-bisheterocyclic substituted thiophenes as selective Dyrk/Clk inhibitors. PLoS ONE.

[21] Tahtouh T., Elkins J.M., Filippakopoulos P., Soundararajan M., Burgy G., Durieu E., Cochet C., Schmid R.S., Lo D.C., Delhommel F. (2012). Selectivity, cocrystal structures, and neuroprotective properties of leucettines, a family of protein kinase inhibitors derived from the marine sponge alkaloid leucettamine B. J. Med. Chem..

[22] Anastassiadis T., Deacon S.W., Devarajan K., Ma H., Peterson J.R. (2011). Comprehensive assay of kinase catalytic activity reveals features of kinase inhibitor selectivity. Nat. Biotechnol..

[23] Cuny G.D., Robin M., Ulyanova N.P., Patnaik D., Pique V., Casano G., Liu J.-F., Lin X., Xian J., Glicksman M.A. (2010). Structure–activity relationship study of acridine analogs as haspin and DYRK2 kinase inhibitors. Bioorg. Med. Chem. Lett..

[24] McLauchilan H., ELlliott M., Cohen P. (2003). The specificities of protein kinase inhibitors: An update. Biochem. J..

[25] Rosenthal A.S., Tanega C., Shen M., Mott B.T., Bougie J.M., Nguyen D.-T., Misteli T., Auld D.S., Maloney D.J., Thomas C.J. (2011). Potent and selective small molecule inhibitors of specific isoforms of Cdc2-like kinases (Clk) and dual specificity tyrosine-phosphorylation-regulated kinases (Dyrk). Bioorg. Med. Chem. Lett..

[26] Coombs T.C., Tanega C., Shen M., Wang J.L., Auld D.S., Gerritz S.W., Schoenen F.J., Thomas C.J., Aubé J. (2013). Small-molecule pyrimidine inhibitors of the cdc2-like (Clk) and dual specificity tyrosine phosphorylation-regulated (Dyrk) kinases: Development of chemical probe ML315. Bioorg. Med. Chem. Lett..

[27] Zhou Q., Phoa A.F., Abbassi R.H., Hoque M., Reekie T.A., Font J.S., Ryan R.M., Stringer B.W., Day B.W., Johns T.G. (2017). Structural optimization and pharmacological evaluation of inhibitors targeting dual-specificity tyrosine phosphorylation-regulated kinases (DYRK) and CDC-like kinases (CLK) in glioblastoma. J. Med. Chem..

[28] Mott B.T., Tanega C., Shen M., Maloney D.J., Shinn P., Leister W., Marugan J.J., Inglese J., Austin C.P., Misteli T. (2009). Evaluation of substituted 6-arylquinazolin-4-amines as potent and selective inhibitors of cdc2-like kinases (Clk). Bioorg. Med. Chem. Lett..

[29] Fedorov O., Huber K., Eisenreich A., Filippakopoulos P., King O., Bullock A.N., Szklarczyk D., Jensen L.J., Fabbro D., Trappe J. (2011). Specific CLK inhibitors from a novel chemotype for regulation of alternative splicing. Chem. Biol..

[30] Mariano M., Hartmann R.W., Engel M. (2016). Systematic diversification of benzylidene heterocycles yields novel inhibitor scaffolds selective for Dyrk1A, Clk1 and CK2. Eur. J. Med. Chem..

