# Supplementary material for: 5-Methoxybenzothiophene-2-Carboxamides as Inhibitors of Clk1/4: Optimization of Selectivity and Cellular Potency
Source: Molecules. 2021 Feb 13;26(4):1001. doi: 10.3390/molecules26041001 (PMC7918793; doi:10.3390/molecules26041001)
Supplement: Supplementary file 1 [file molecules-26-01001-s001.pdf]

Supplementary Material

5-Methoxybenzothiophene-2-carboxamides as inhibitors of Clk1/4: optimization of the selectivity and cellular potency

*Ahmed K. ElHady[1,2], Dalia S. El-Gamil[1], Po-Jen Chen[3,4], Tsong-Long Hwang[3,5,6,7], Ashraf H. Abadi[1], Mohammad Abdel-Halim[1], Matthias Engel[8]**

AUTHOR ADDRESS

[1]Department of Pharmaceutical Chemistry, Faculty of Pharmacy and Biotechnology, German University in Cairo, Cairo 11835, Egypt.

[2]School of Life and Medical Sciences, University of Hertfordshire hosted by Global Academic Foundation, New Administrative Capital, Cairo, Egypt.

[3]Graduate Institute of Natural Products, College of Medicine, Chang Gung University, Taoyuan 333, Taiwan.

[4]Department of Cosmetic Science, Providence University, Taichung 433, Taiwan

[5]Research Center for Chinese Herbal Medicine, Graduate Institute of Health Industry Technology, College of Human Ecology, Chang Gung University of Science and Technology, Taoyuan 333, Taiwan.

[6]Department of Anesthesiology, Chang Gung Memorial Hospital, Taoyuan 333, Taiwan.

[7]Department of Chemical Engineering, Ming Chi University of Technology, New Taipei City 243, Taiwan.

[8]Pharmaceutical and Medicinal Chemistry, Saarland University, Campus C2.3, D-66123 Saarbrücken, Germany

*Correspondence:

Dr. Matthias Engel. Phone: +49 681 302 70312. Fax: +49 681 302 70308. E-mail: ma.engel@mx.uni-saarland.de.

Contents

Figure S1 3

Figure S2 4

Figure S3 5

^{1}H-NMR (500 MHz, DMSO) and ^{13}C-NMR (126 MHz, DMSO) spectra of all synthesized compounds. 14

Figure S1

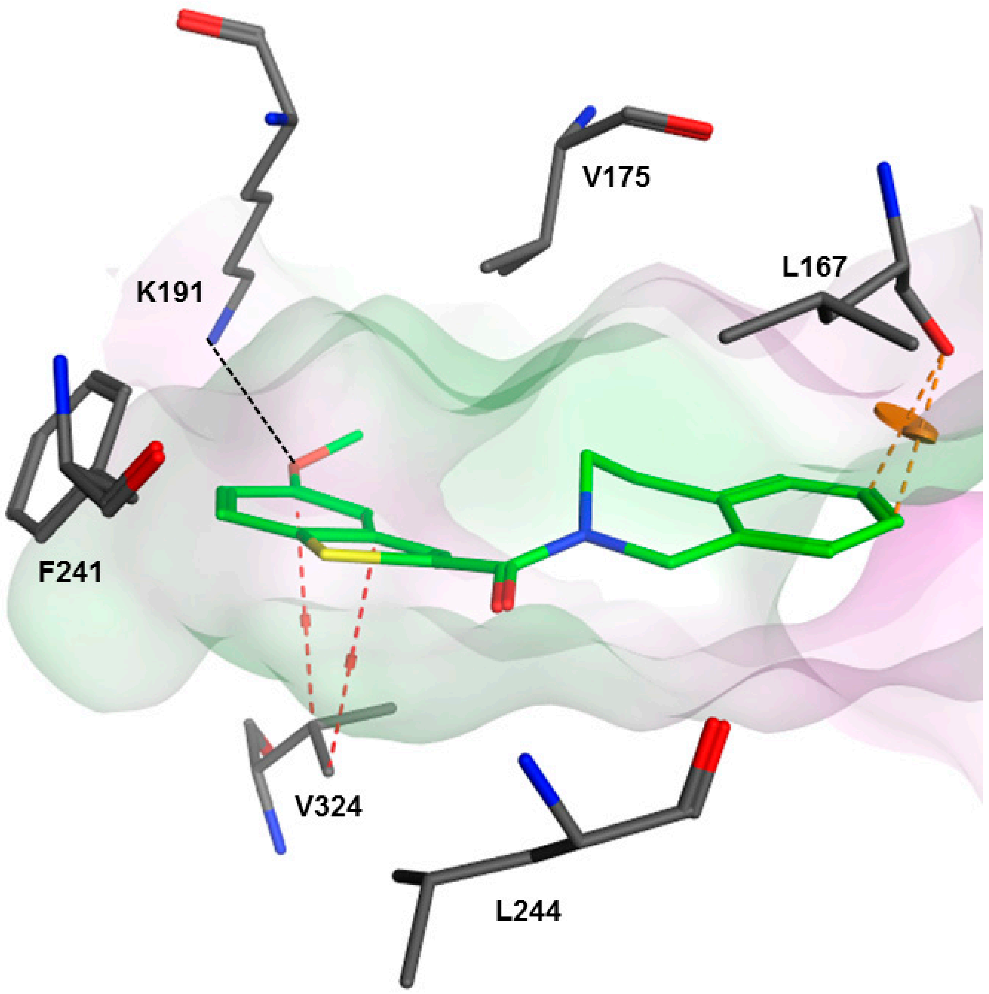

Figure S1: Molecular docking of compound **5a** (green) in the ATP binding pocket of Clk1 (PDB code of the coordinates: 1Z57) using MOE. Depicted is the least impaired potential binding pose. Although H-bonds were predicted with Lys191 (indicated in black), and CH-π interactions between the benzothiophene core and Val324, the steric clash with Leu167 (indicated in orange) is expected to strongly compromise the potential binding affinity. In addition, no H-bond was formed between Leu244 and the carbonyl oxygen of **5a**.

Figure S2

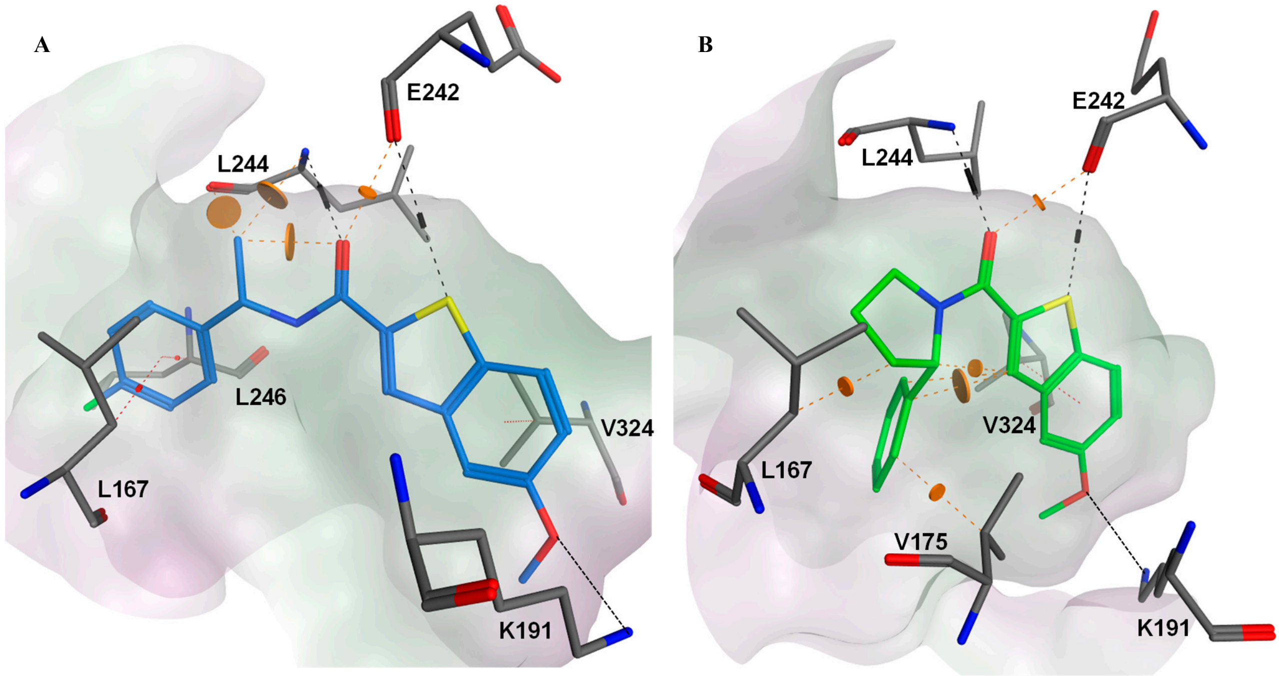

Figure S2: Molecular docking of compounds **3a (*S*)** (blue) and **6a** (green). **3a (*S*)** and **6a** were docked in the ATP binding pocket of Clk1 (PDB code: 1Z57) using MOE, and the binding poses with the lowest steric interferences were selected. (A) **3a (*S*)** (blue) was predicted to form H-bonds with Leu244 and Lys191 (indicated by black dashed lines) and CH-π interactions with Leu167, Leu264 and Val324 (red lines). However, steric clashes (orange) with Leu244 and Glu242 were also inherent to this pose. (B) **6a** (green) was predicted to form H-bonds with Leu244 and Lys191 (black dashed lines), in addition to a CH-π interaction between the benzothiophene core and Val324 (red line). However, steric clashes with Leu167 and Val175 as well as intramolecular steric interference also occurred with this pose (indicated in orange).

Figure S3

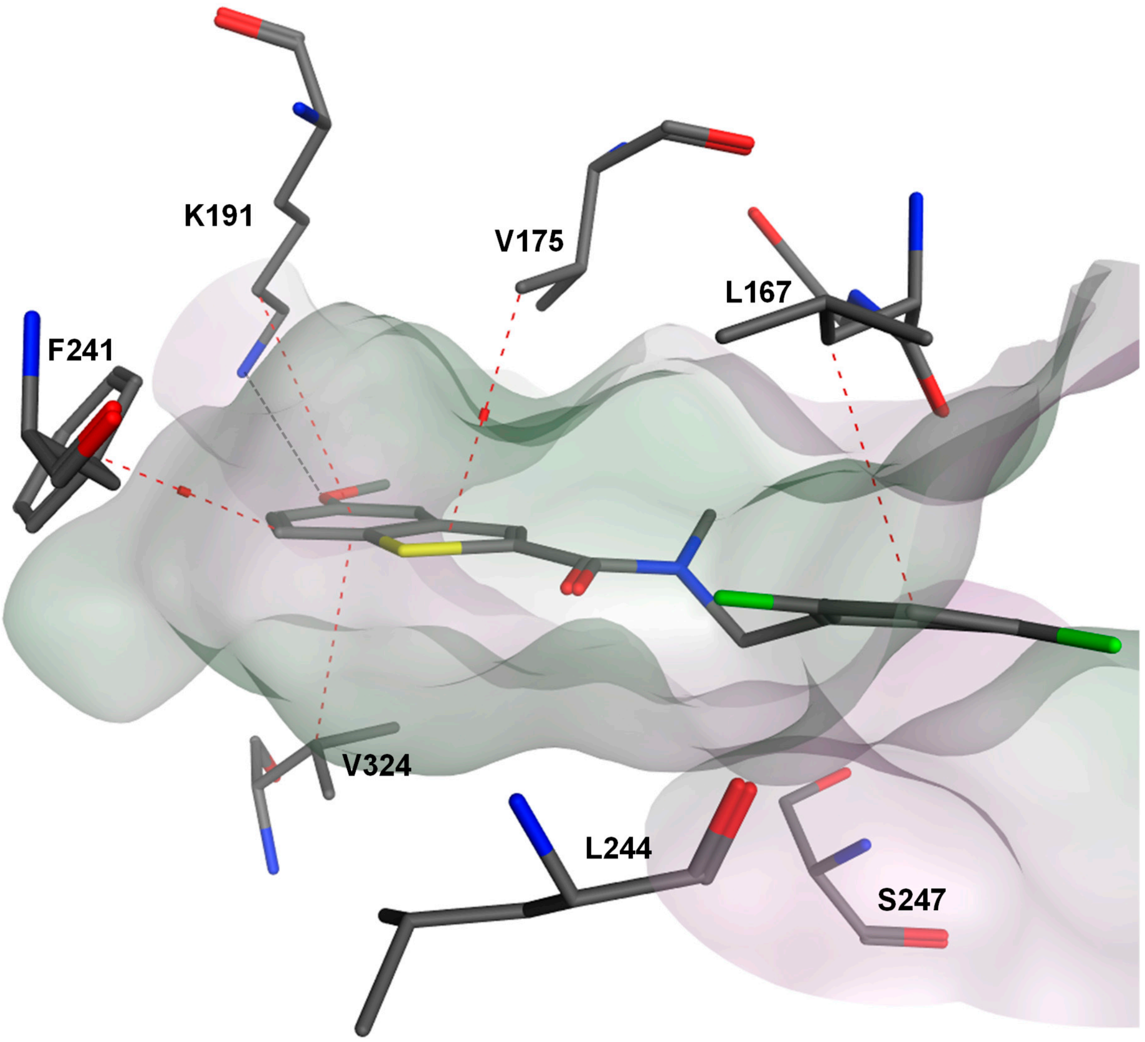

Figure S3: Molecular docking of compound **9b** (dark grey) in the binding pocket of Clk1 (PDB code 1Z57) using MOE. **9b** was predicted to interact through an H-bond with Lys191 (indicated by black dashed lines), CH-π interactions with Leu167, Val175, Lys191 and Val324 residues (red dashed lines), and an edge-to-face CH-π interaction with Phe241. However, the H-bond between Leu244 and the carbonyl oxygen could not form, suggesting a strong reduction of the overall binding affinity.

^{1}H-NMR (500 MHz, DMSO) and ^{13}C-NMR (126 MHz, DMSO) spectra of all synthesized compounds.

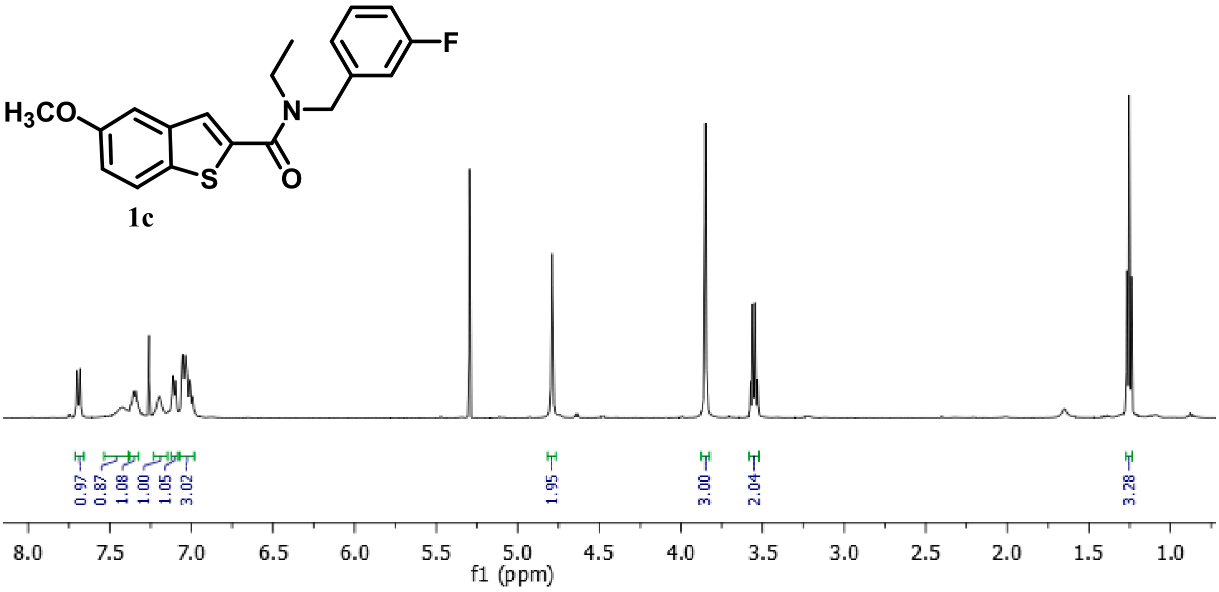

1c

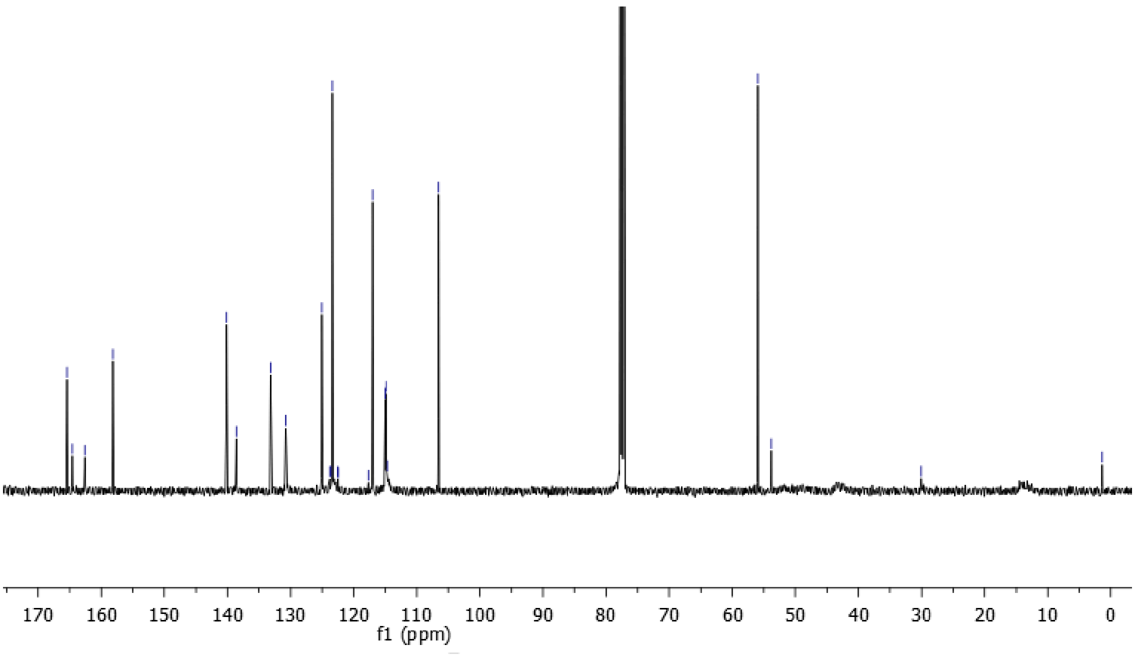

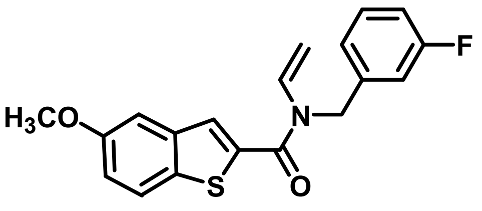
H3CO
N
O
S
F

1d

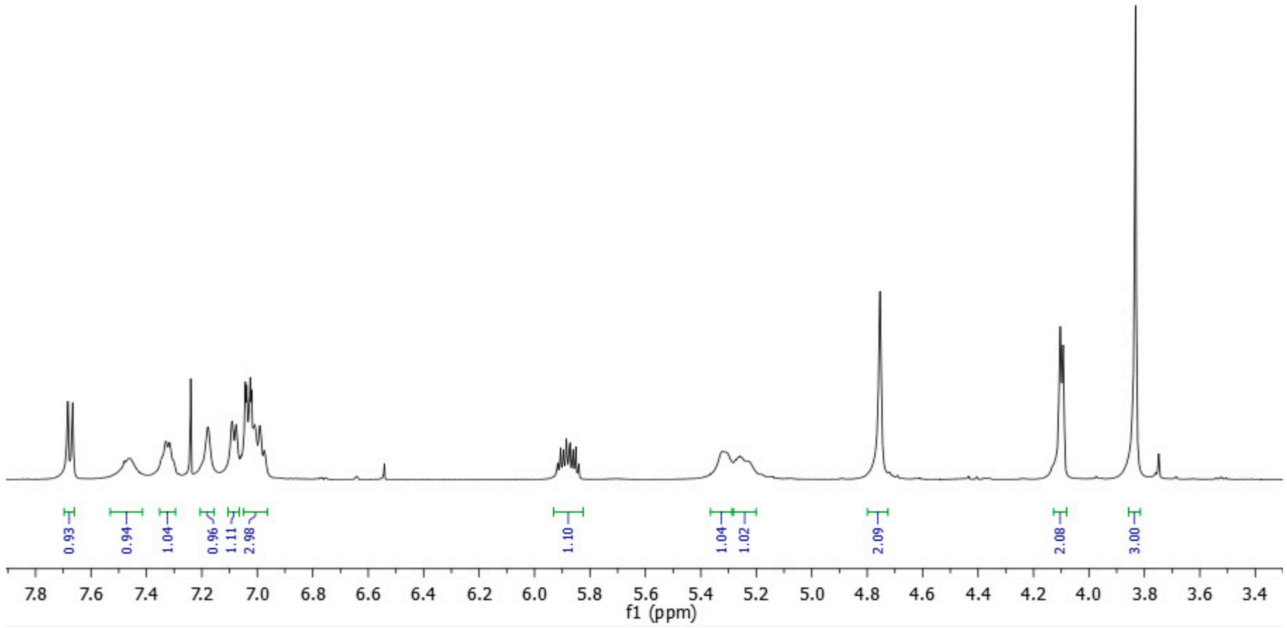
0.93
0.94
1.04
0.96
1.11
2.98
1.10
1.04
1.02
2.09
2.08
3.00
7.8 7.6 7.4 7.2 7.0 6.8 6.6 6.4 6.2 6.0 5.8 5.6 5.4 5.2 5.0 4.8 4.6 4.4 4.2 4.0 3.8 3.6 3.4
f1 (ppm)

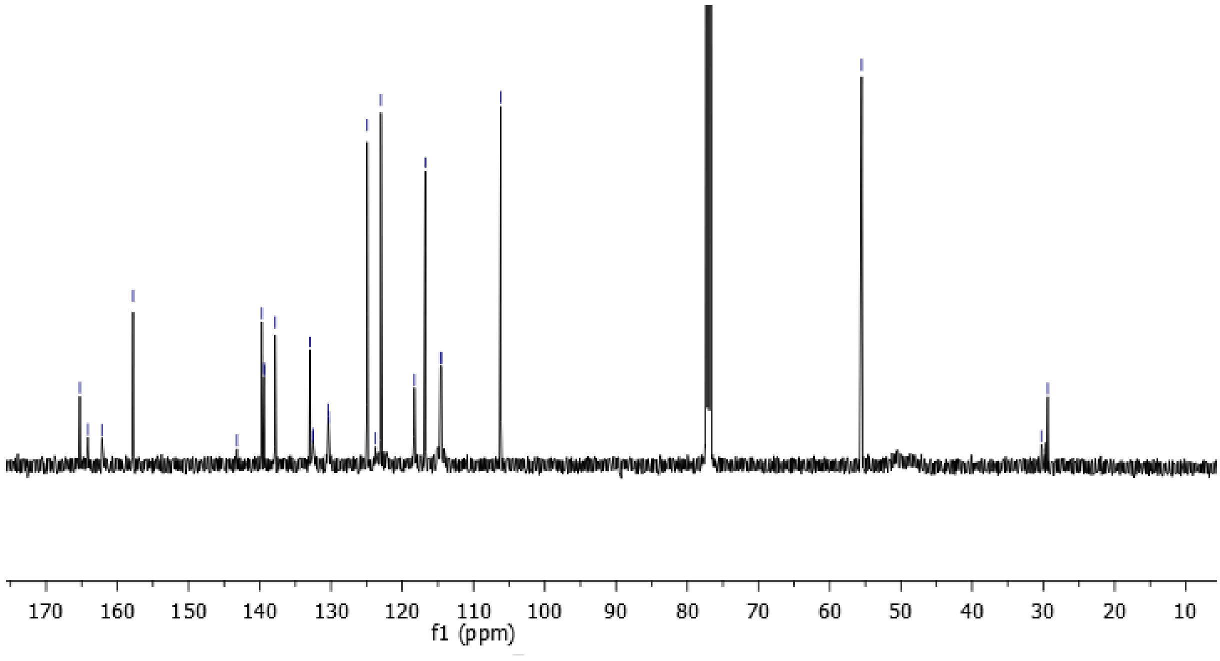
170 160 150 140 130 120 110 100 90 80 70 60 50 40 30 20 10
f1 (ppm)

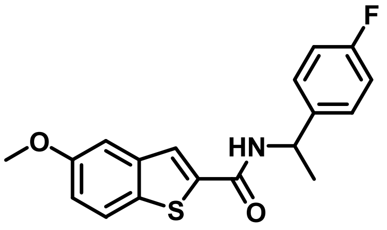
O
HN
F
S
O

3a

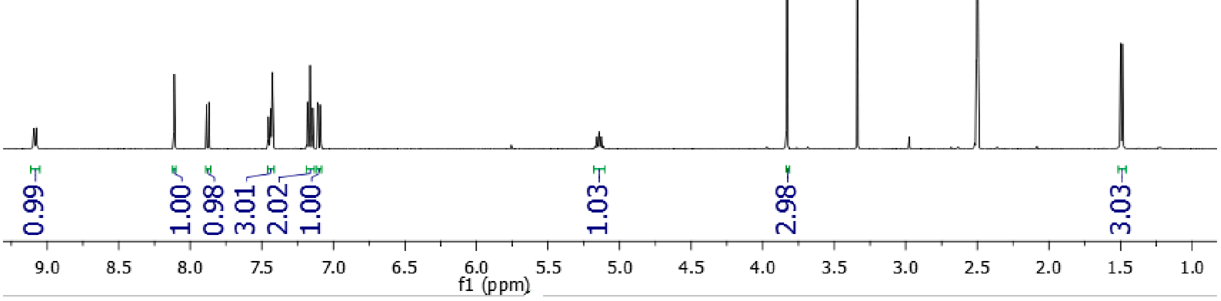
0.99
1.00
0.98
3.01
2.02
1.00
1.03
2.98
3.03
9.0 8.5 8.0 7.5 7.0 6.5 6.0 5.5 5.0 4.5 4.0 3.5 3.0 2.5 2.0 1.5 1.0
f1 (ppm)

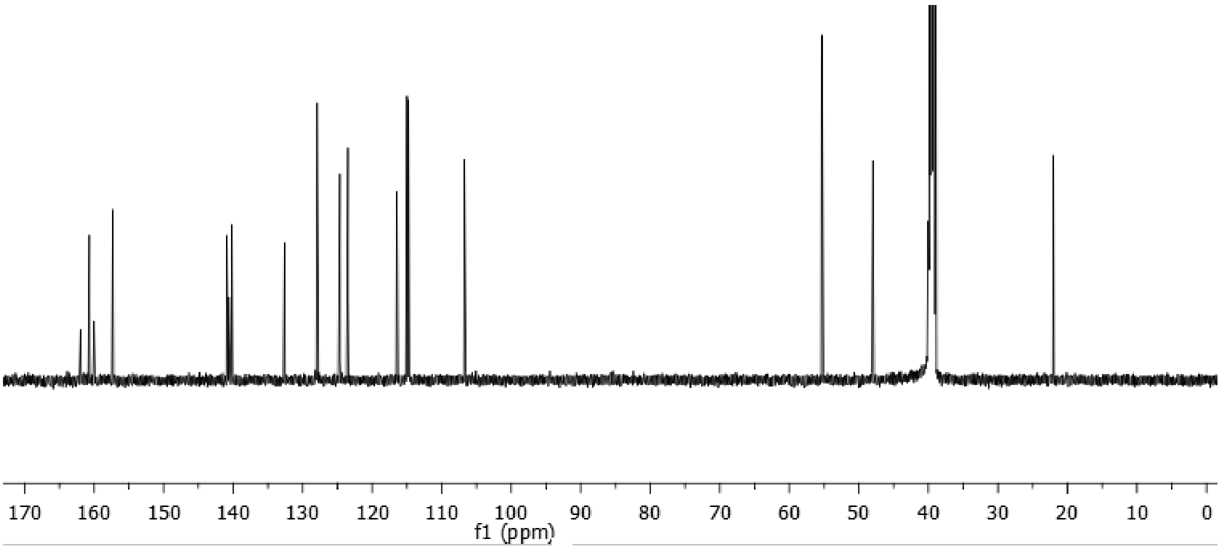
170 160 150 140 130 120 110 100 90 80 70 60 50 40 30 20 10 0
f1 (ppm)

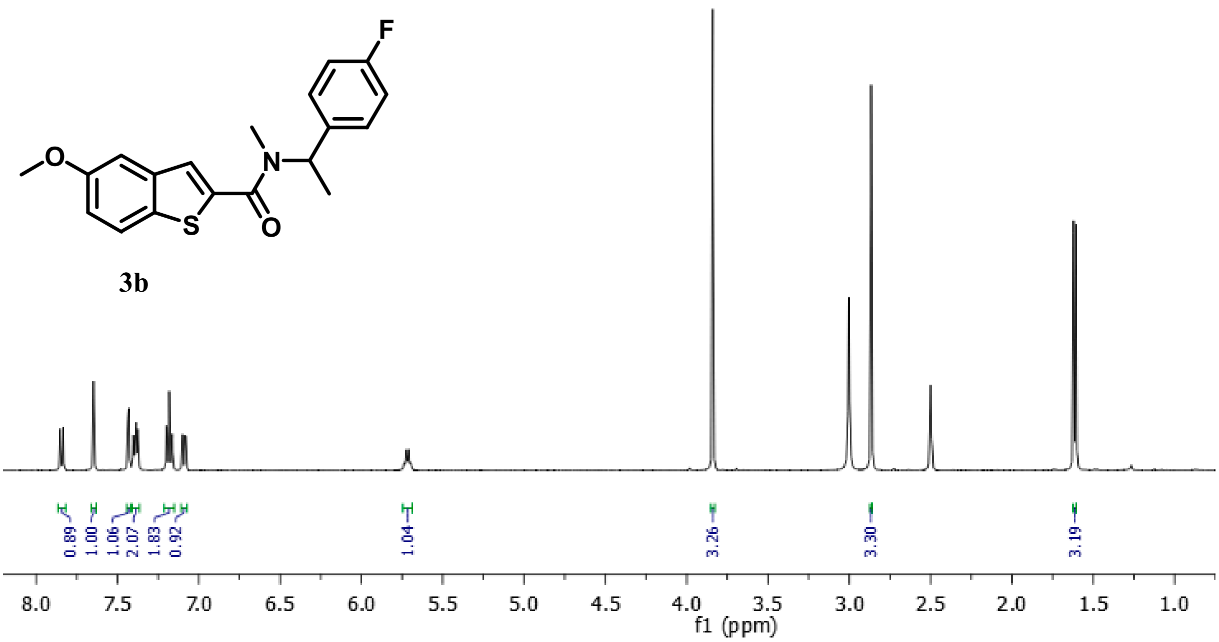
O
N
S
O
F
3b
0.89
1.00
1.06
2.07
1.83
0.92
1.04
3.26
3.30
3.19
8.0 7.5 7.0 6.5 6.0 5.5 5.0 4.5 4.0 3.5 3.0 2.5 2.0 1.5 1.0
f1 (ppm)

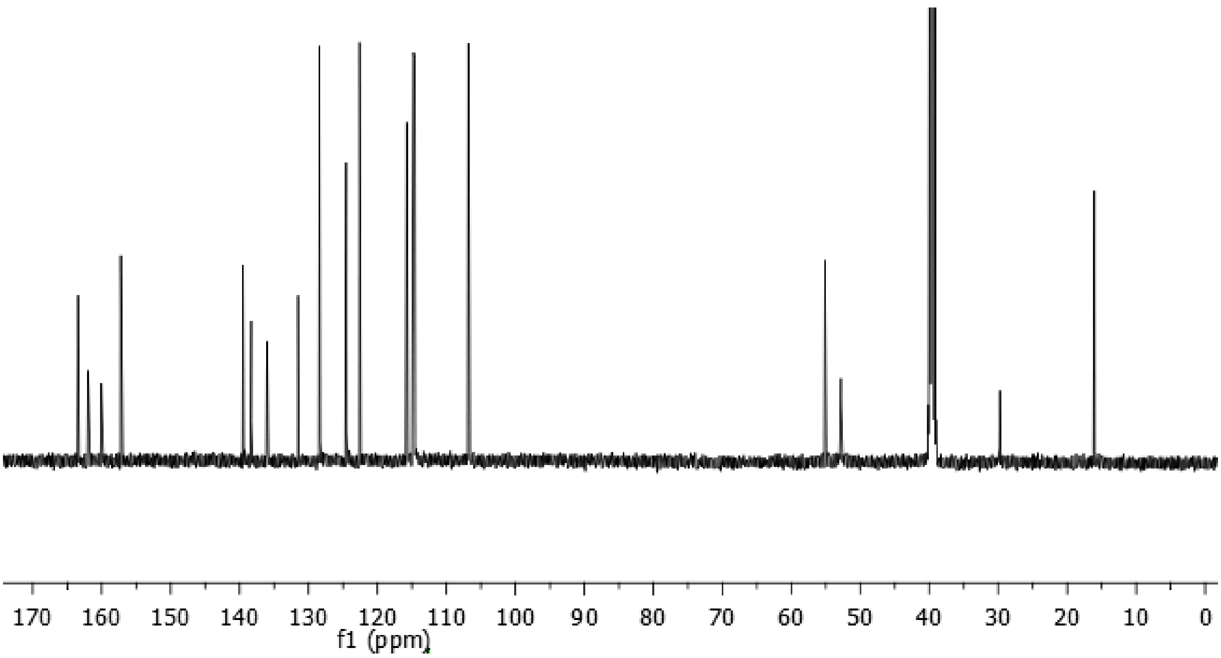
170 160 150 140 130 120 110 100 90 80 70 60 50 40 30 20 10 0
f1 (ppm)

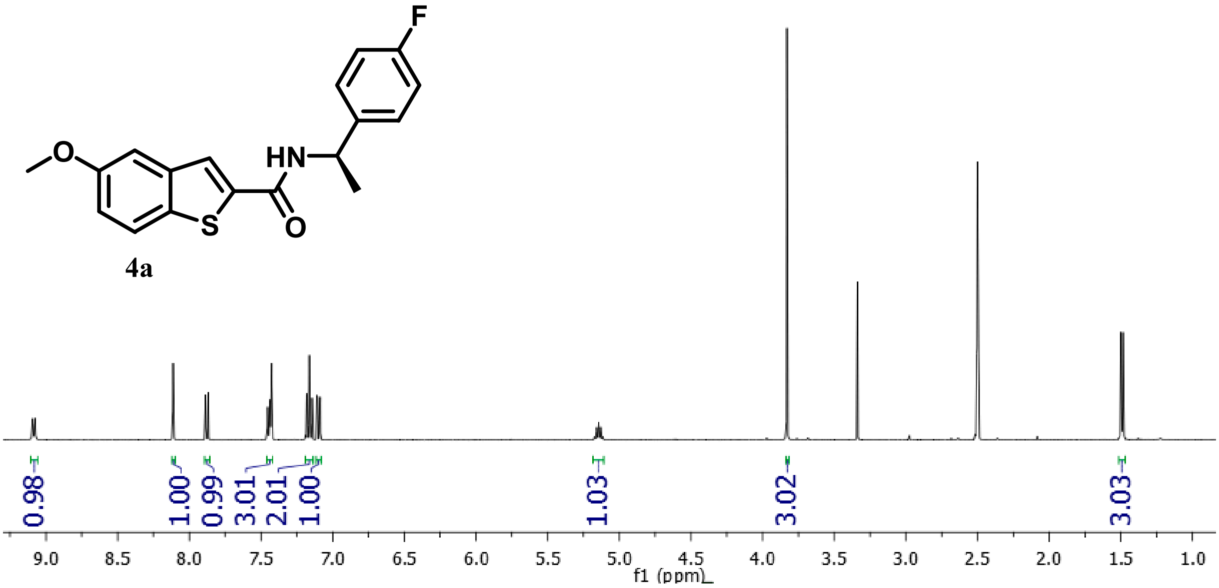
F
HN
O
O
S
4a
0.98
1.00
0.99
3.01
2.01
1.00
1.03
3.02
3.03
f1 (ppm)

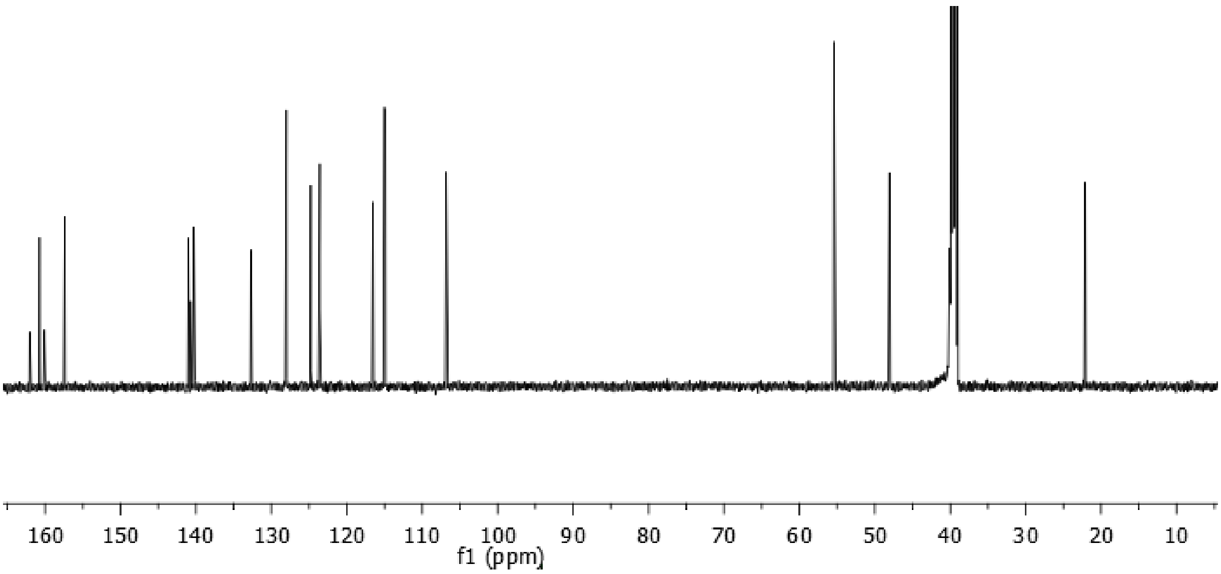
f1 (ppm)

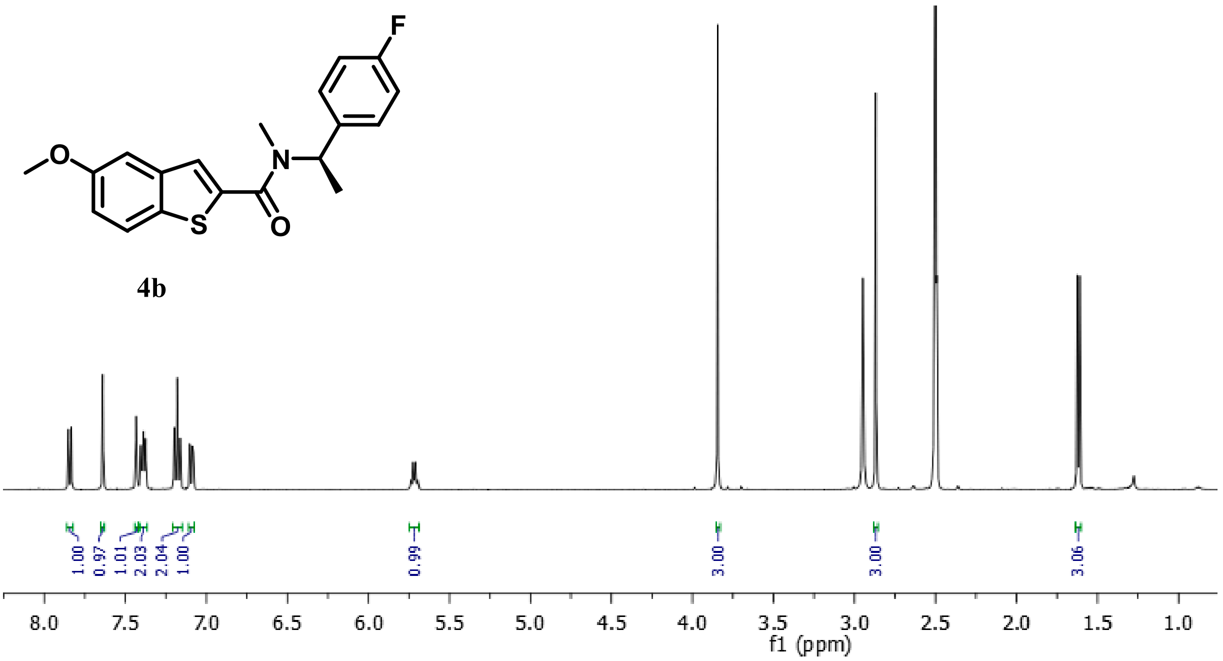
F
O
N
S
O
4b
1.00
0.97
1.01
2.03
2.04
1.00
0.99
3.00
3.00
3.06
8.0 7.5 7.0 6.5 6.0 5.5 5.0 4.5 4.0 3.5 3.0 2.5 2.0 1.5 1.0
f1 (ppm)

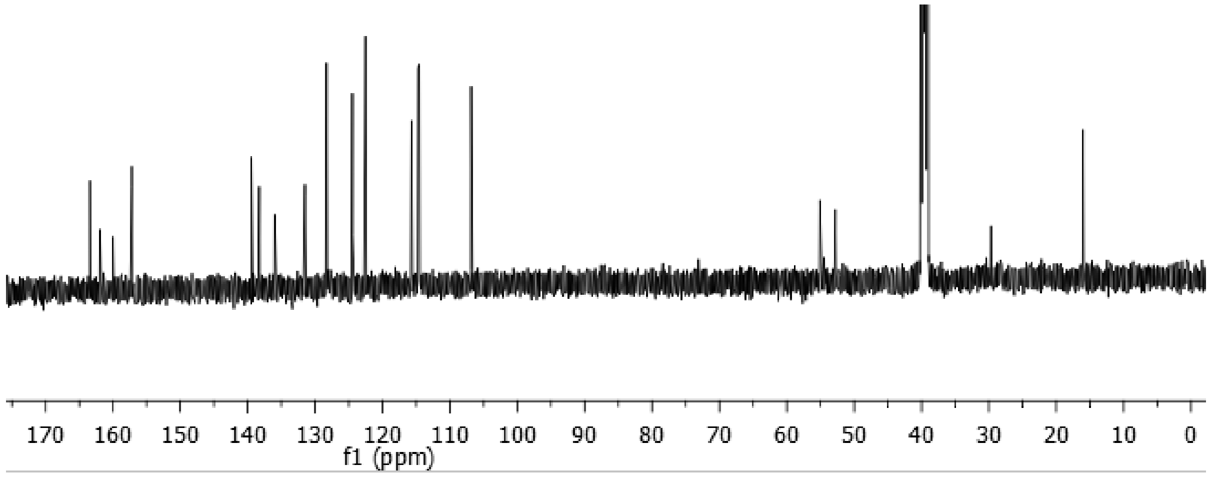
170 160 150 140 130 120 110 100 90 80 70 60 50 40 30 20 10 0
f1 (ppm)

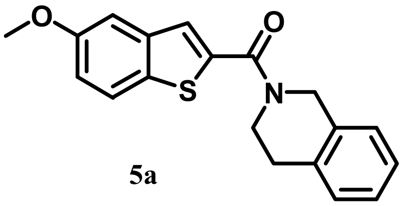
O
O
N
S

5a

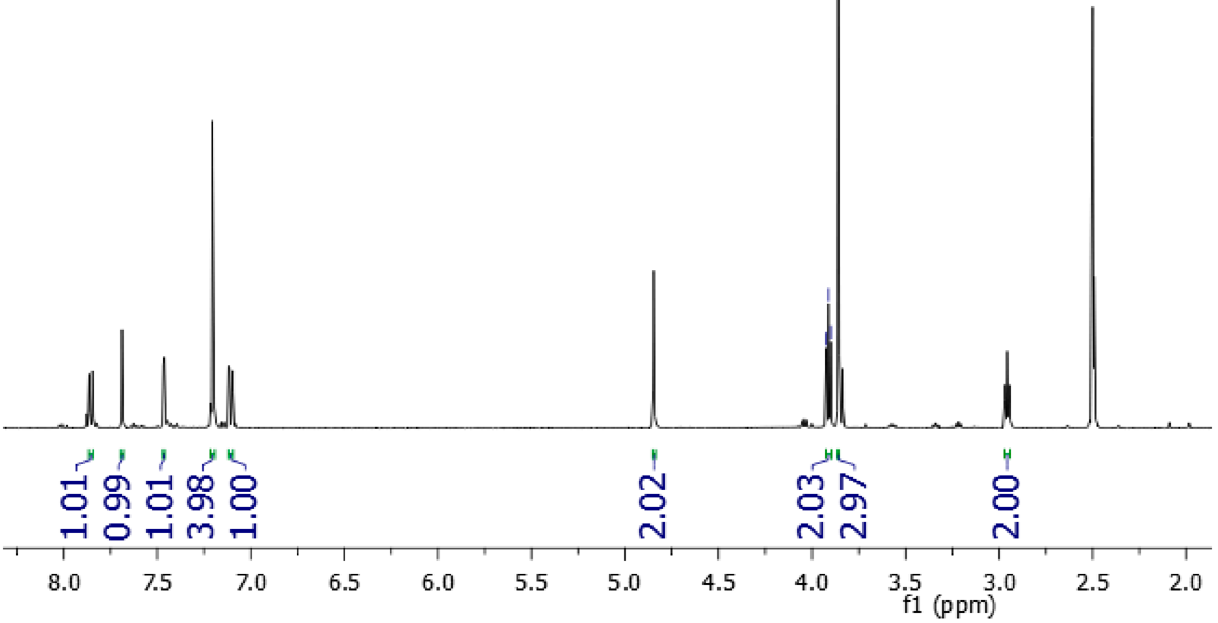
1.01
0.99
1.01
3.98
1.00
2.02
2.03
2.97
2.00
8.0 7.5 7.0 6.5 6.0 5.5 5.0 4.5 4.0 3.5 3.0 2.5 2.0
f1 (ppm)

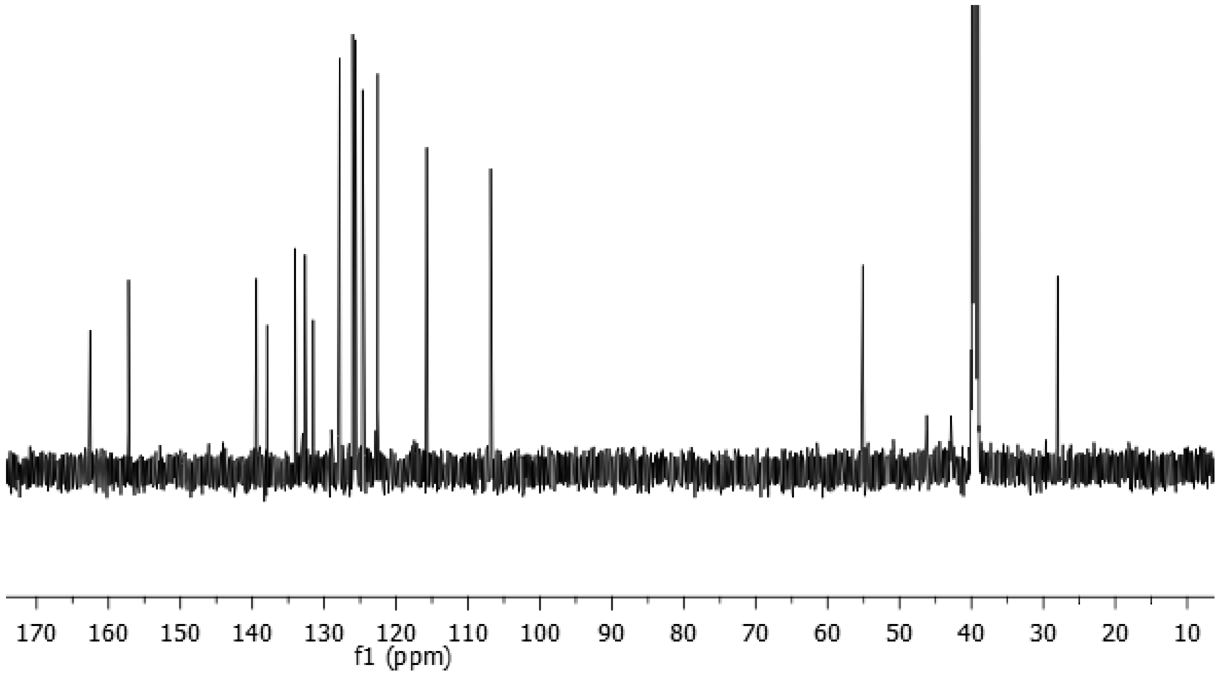
170 160 150 140 130 120 110 100 90 80 70 60 50 40 30 20 10
f1 (ppm)

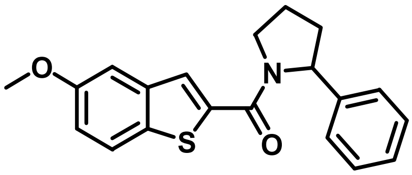
O
N
S
O

6a

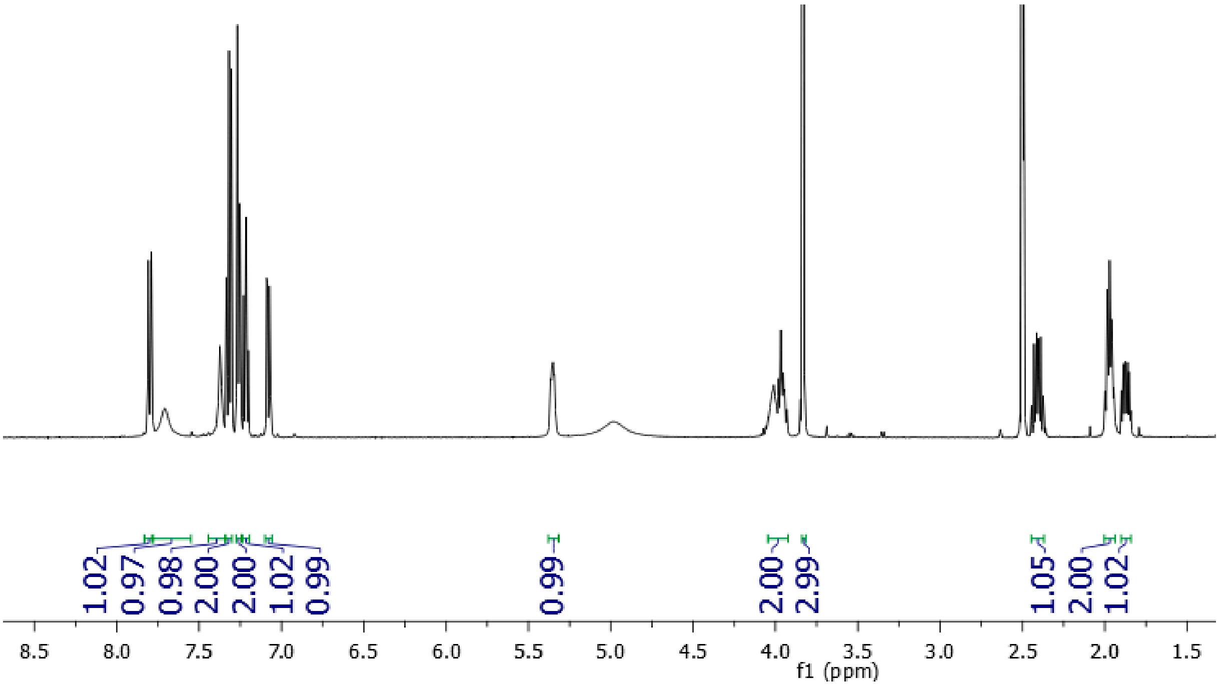
1.02
0.97
0.98
2.00
2.00
1.02
0.99
0.99
2.00
2.99
1.05
2.00
1.02
8.5 8.0 7.5 7.0 6.5 6.0 5.5 5.0 4.5 4.0 3.5 3.0 2.5 2.0 1.5
f1 (ppm)

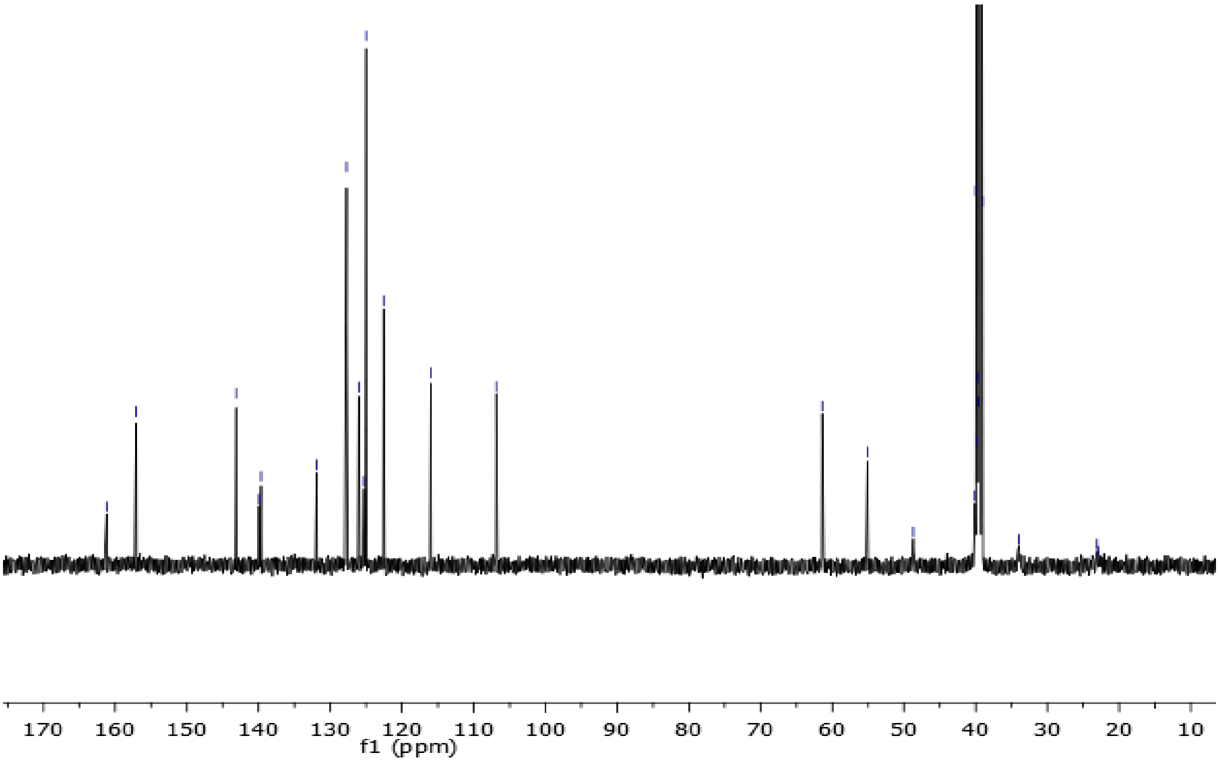
170 160 150 140 130 120 110 100 90 80 70 60 50 40 30 20 10
f1 (ppm)

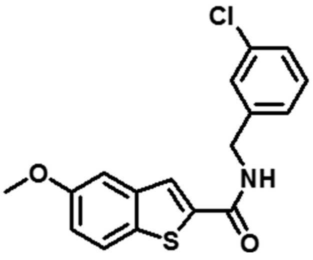
Cl
O
NH
S
O

7a

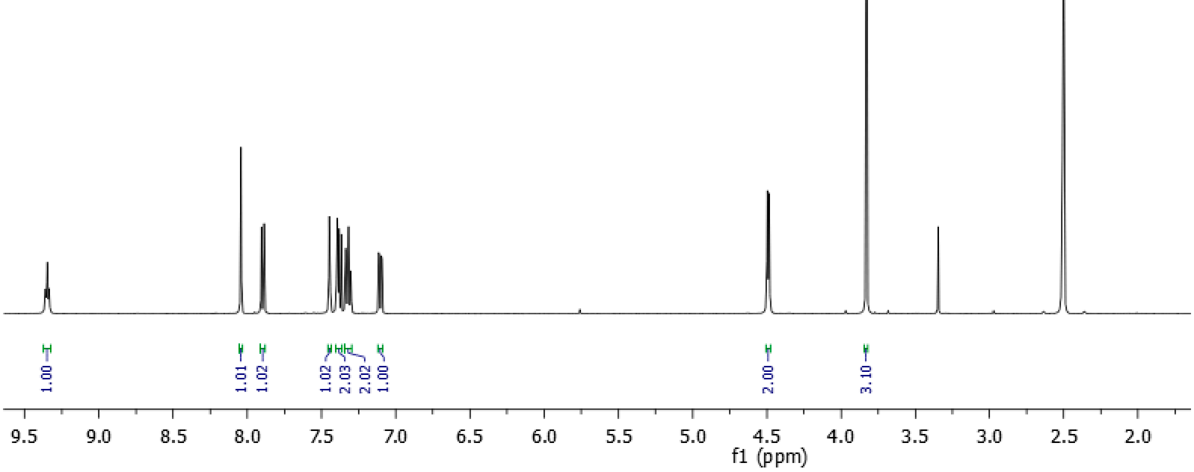
1.00
1.01
1.02
1.02
2.03
2.02
1.00
2.00
3.10
9.5 9.0 8.5 8.0 7.5 7.0 6.5 6.0 5.5 5.0 4.5 4.0 3.5 3.0 2.5 2.0
f1 (ppm)

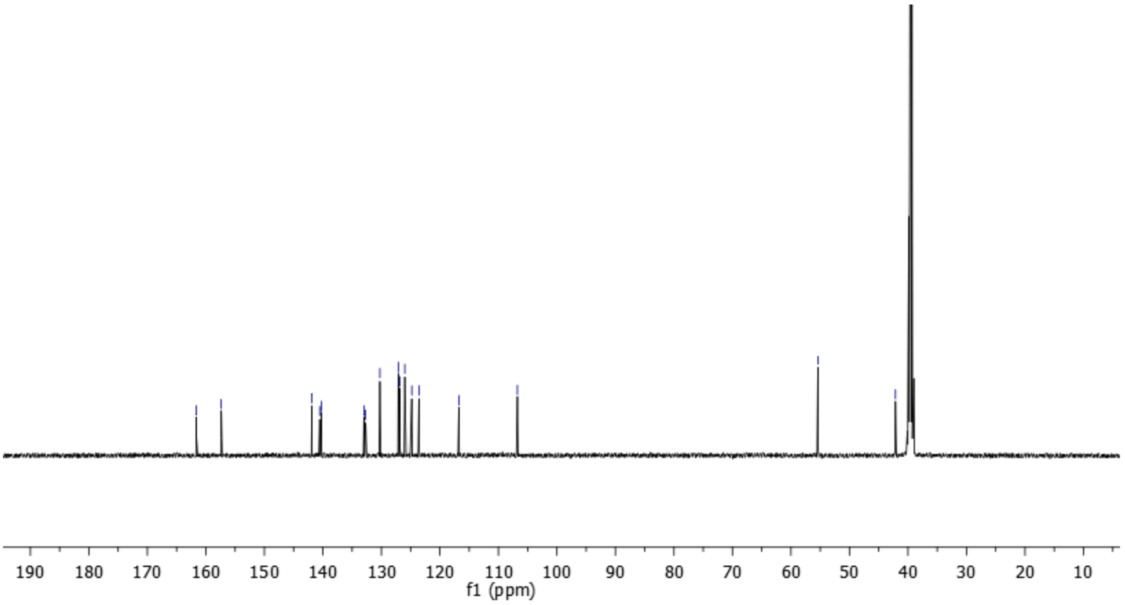
190 180 170 160 150 140 130 120 110 100 90 80 70 60 50 40 30 20 10
f1 (ppm)

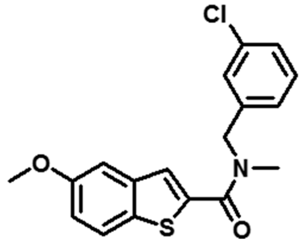
Cl
N
O
S
O

7b

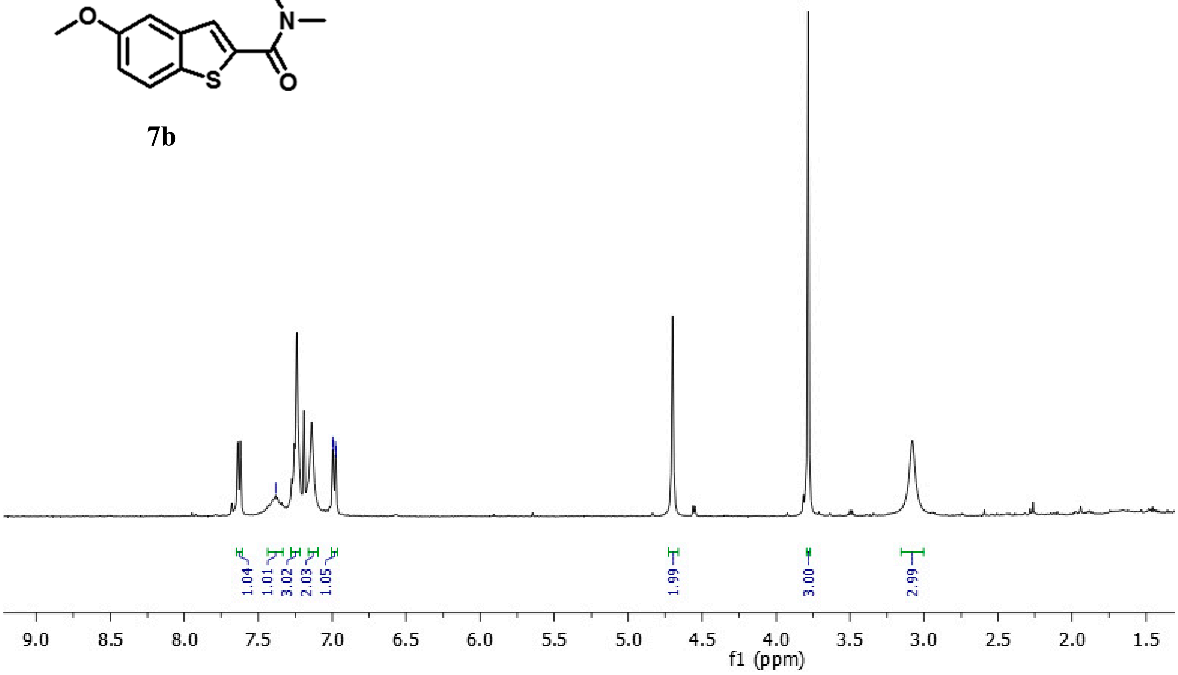
1.04
1.01
3.02
2.03
1.05
1.99
3.00
2.99
9.0 8.5 8.0 7.5 7.0 6.5 6.0 5.5 5.0 4.5 4.0 3.5 3.0 2.5 2.0 1.5
f1 (ppm)

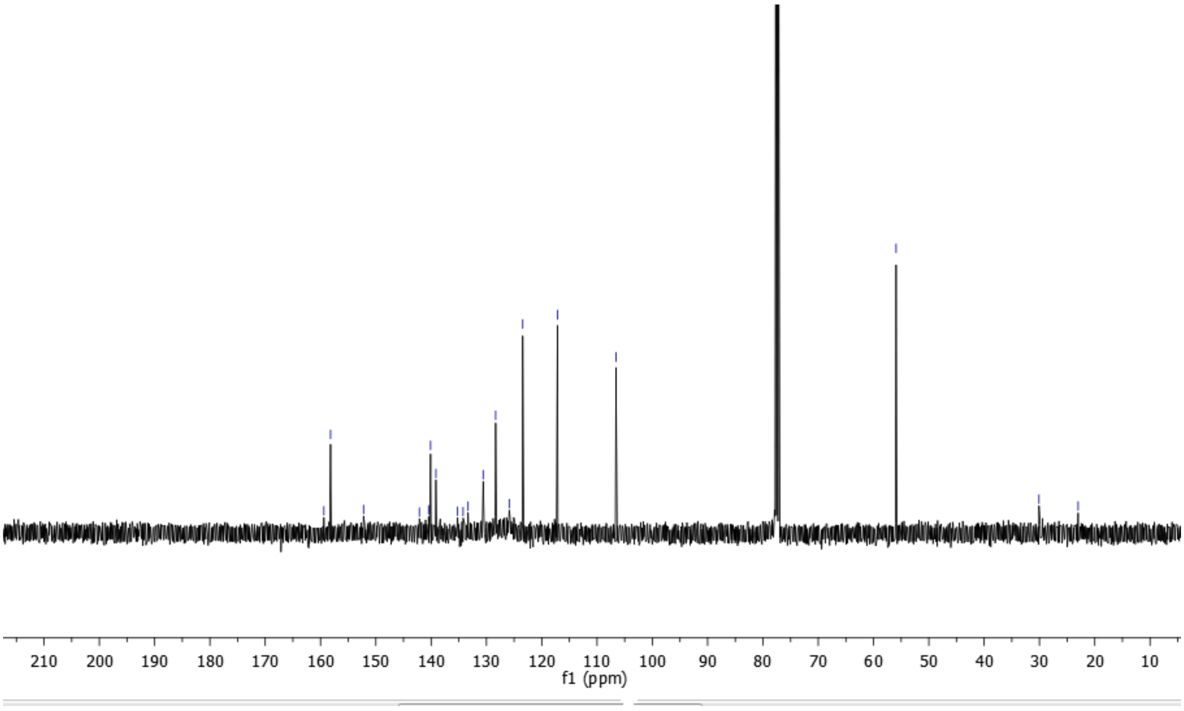
210 200 190 180 170 160 150 140 130 120 110 100 90 80 70 60 50 40 30 20 10
f1 (ppm)

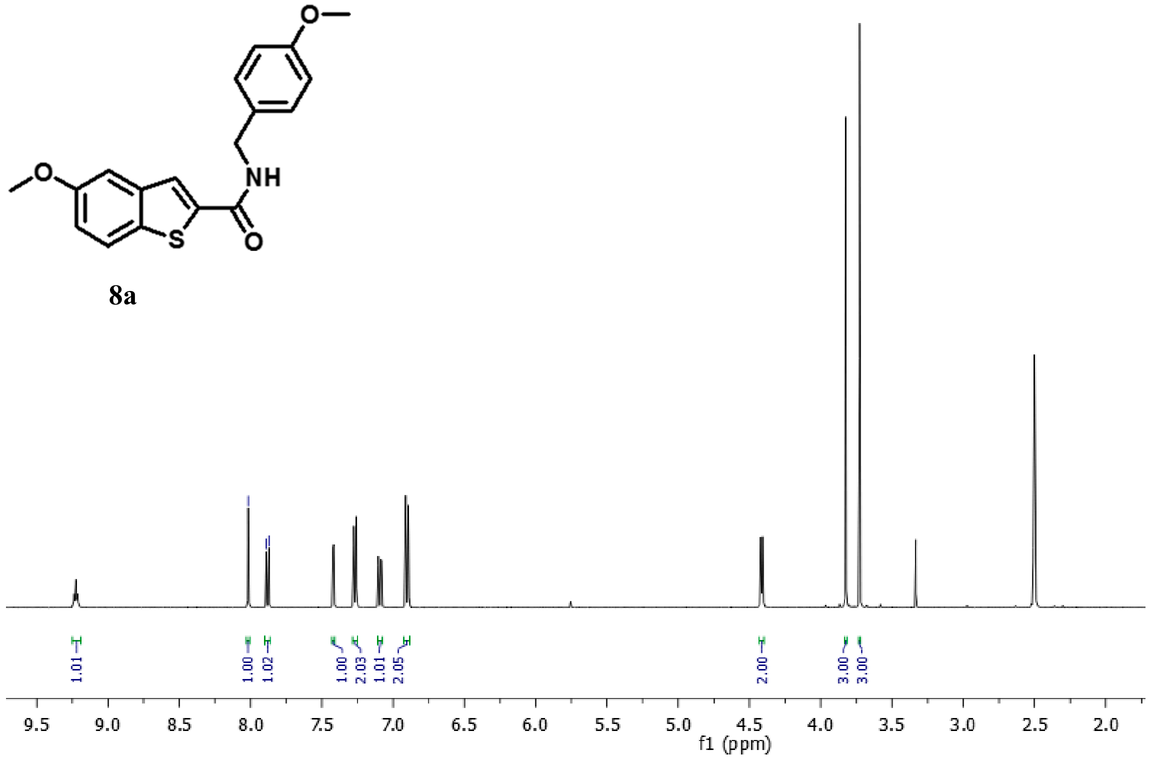
O
NH
O
S
8a
1.01
1.00
1.02
1.00
2.03
1.01
2.05
2.00
3.00
3.00
9.5 9.0 8.5 8.0 7.5 7.0 6.5 6.0 5.5 5.0 4.5 4.0 3.5 3.0 2.5 2.0
f1 (ppm)

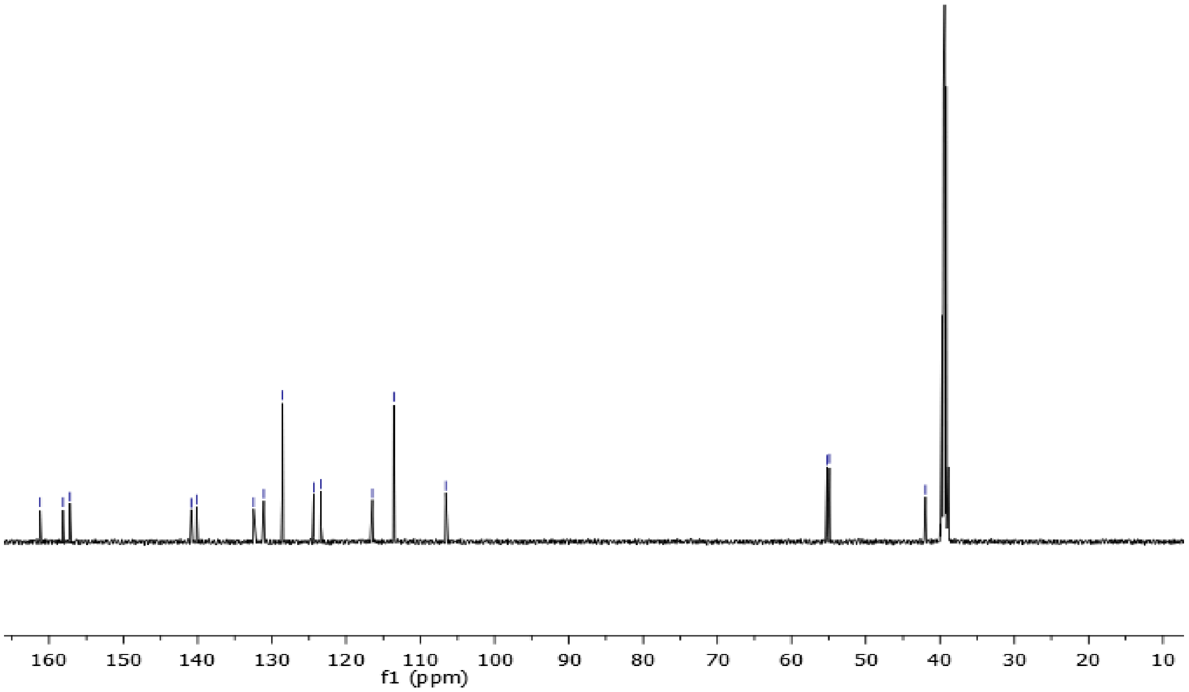
160 150 140 130 120 110 100 90 80 70 60 50 40 30 20 10
f1 (ppm)

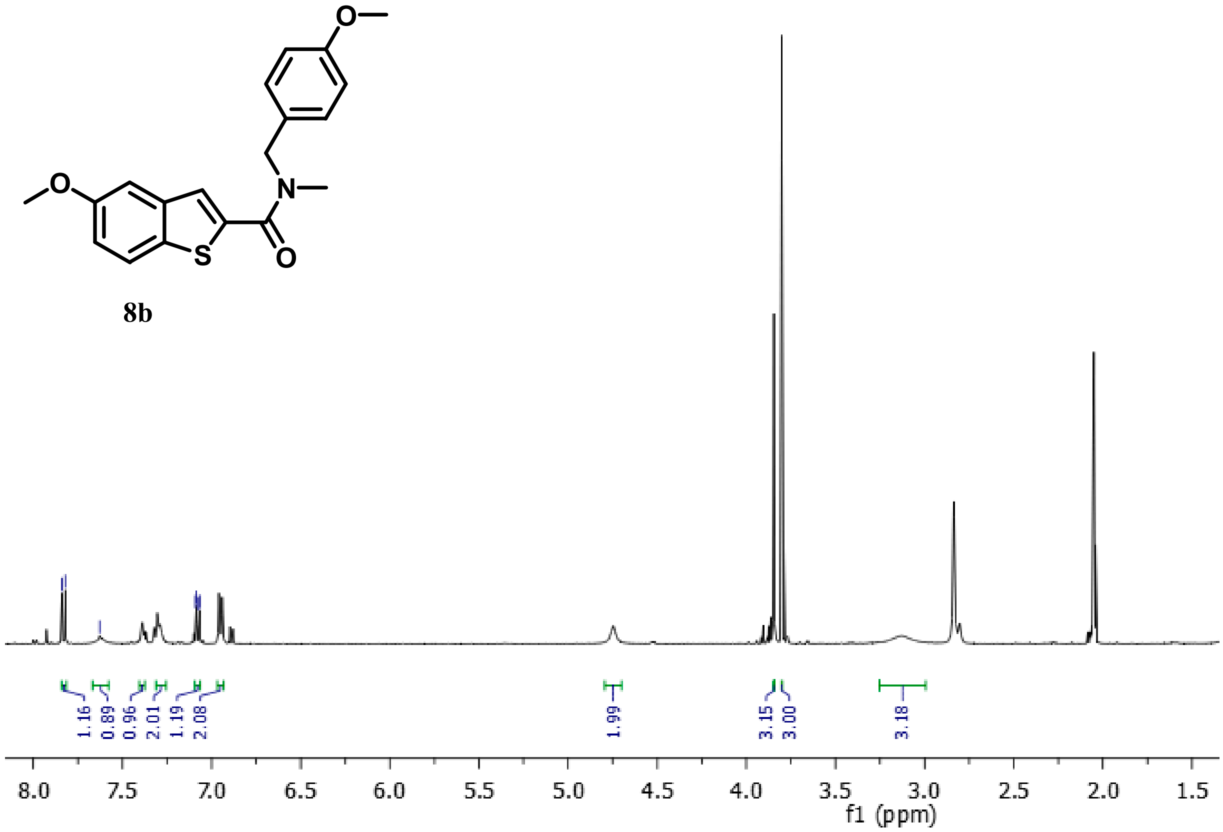
8b

1.16
0.89
0.96
2.01
1.19
2.08
1.99
3.15
3.00
3.18
8.0
7.5
7.0
6.5
6.0
5.5
5.0
4.5
4.0
3.5
3.0
2.5
2.0
1.5
f1 (ppm)

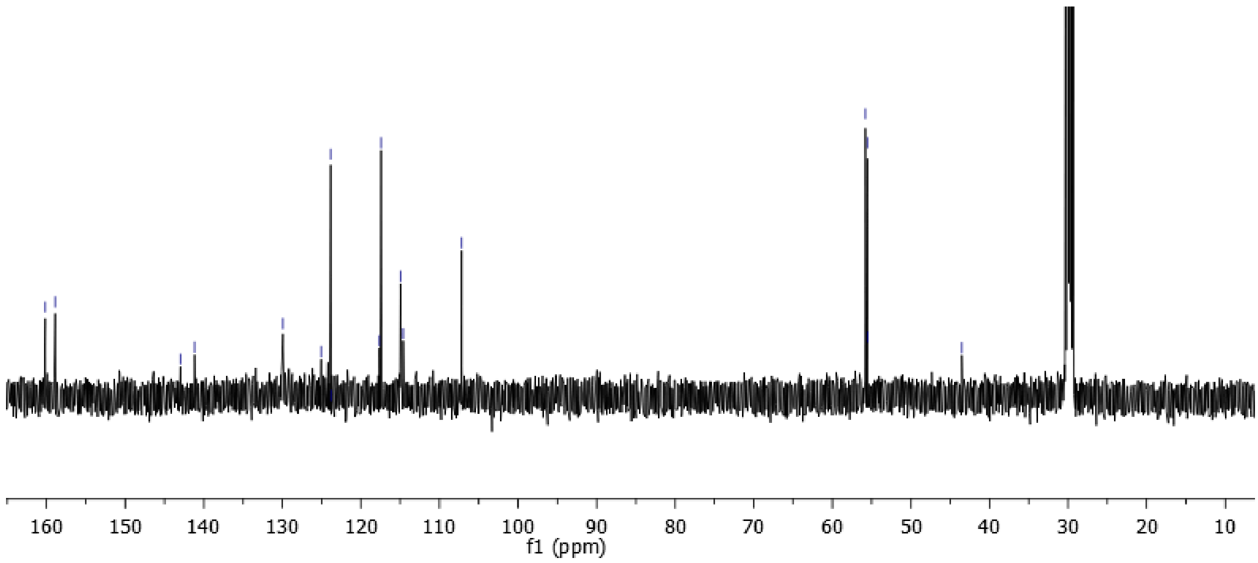
160
150
140
130
120
110
100
90
80
70
60
50
40
30
20
10
f1 (ppm)

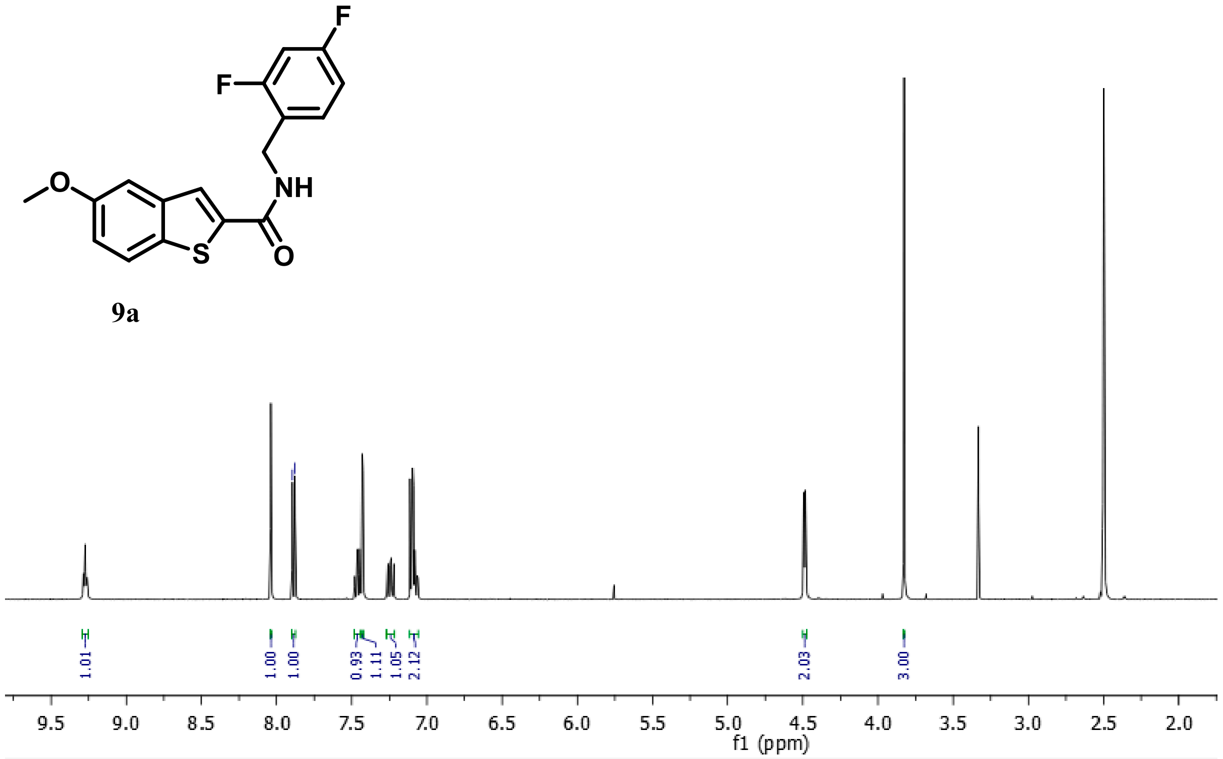
F
F
NH
O
S
O
1.01
1.00
1.00
0.93
1.11
1.05
2.12
2.03
3.00
9.5 9.0 8.5 8.0 7.5 7.0 6.5 6.0 5.5 5.0 4.5 4.0 3.5 3.0 2.5 2.0
f1 (ppm)

9a

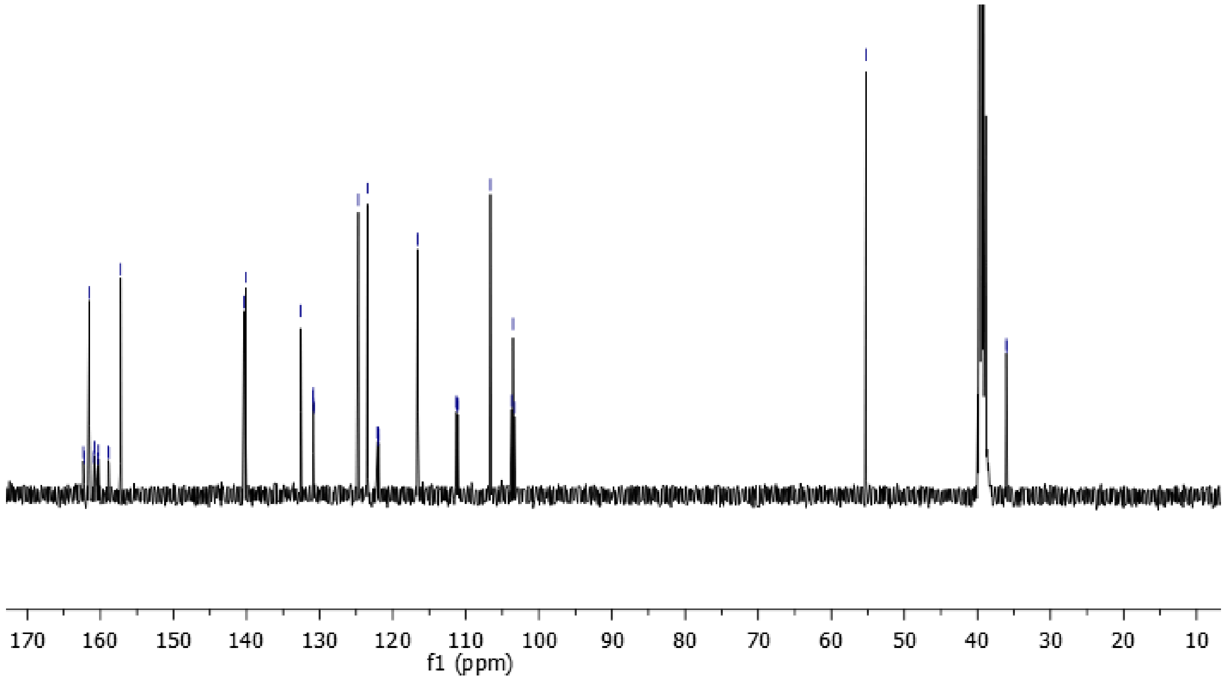
170 160 150 140 130 120 110 100 90 80 70 60 50 40 30 20 10
f1 (ppm)

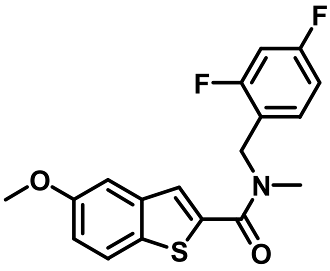
F
F
O
N
S
O

9b

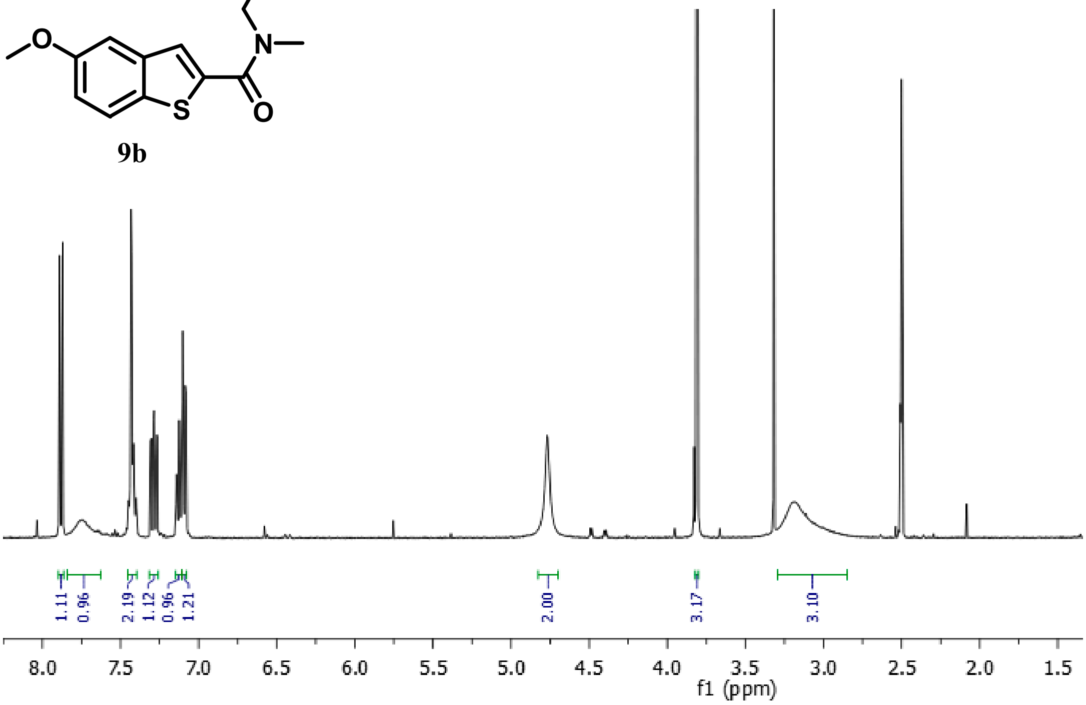
1.11
0.96
2.19
1.12
0.96
1.21
2.00
3.17
3.10
8.0
7.5
7.0
6.5
6.0
5.5
5.0
4.5
4.0
3.5
3.0
2.5
2.0
1.5
f1 (ppm)

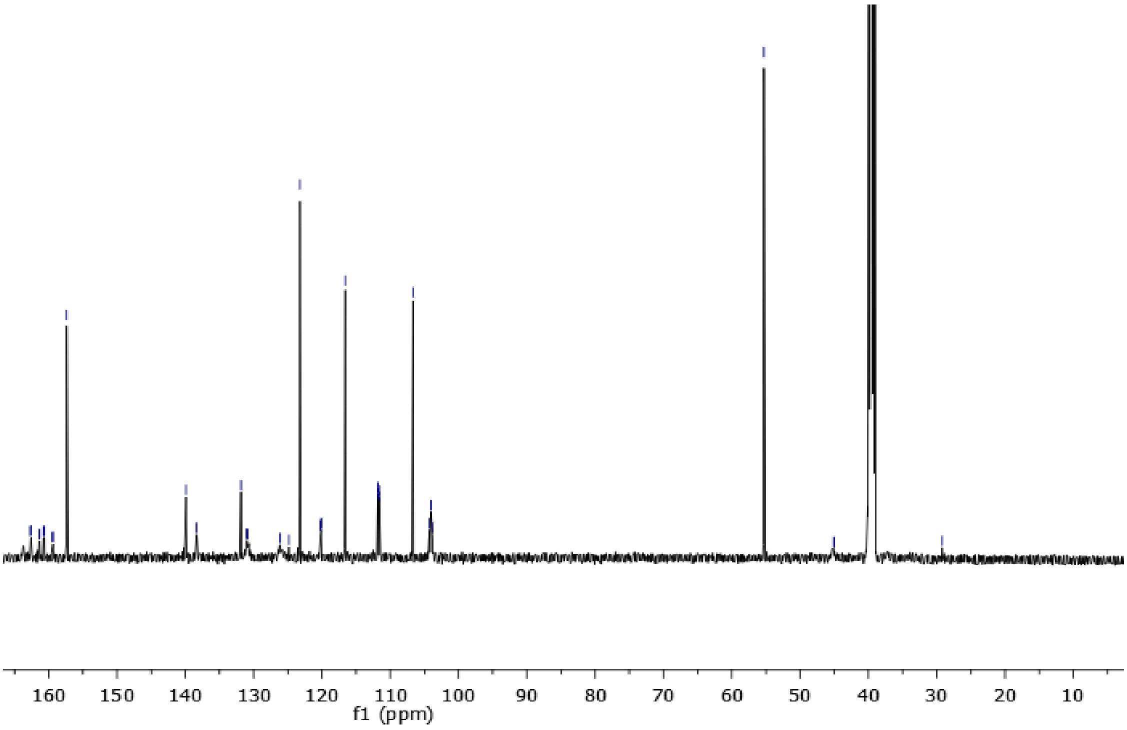
160
150
140
130
120
110
100
90
80
70
60
50
40
30
20
10
f1 (ppm)

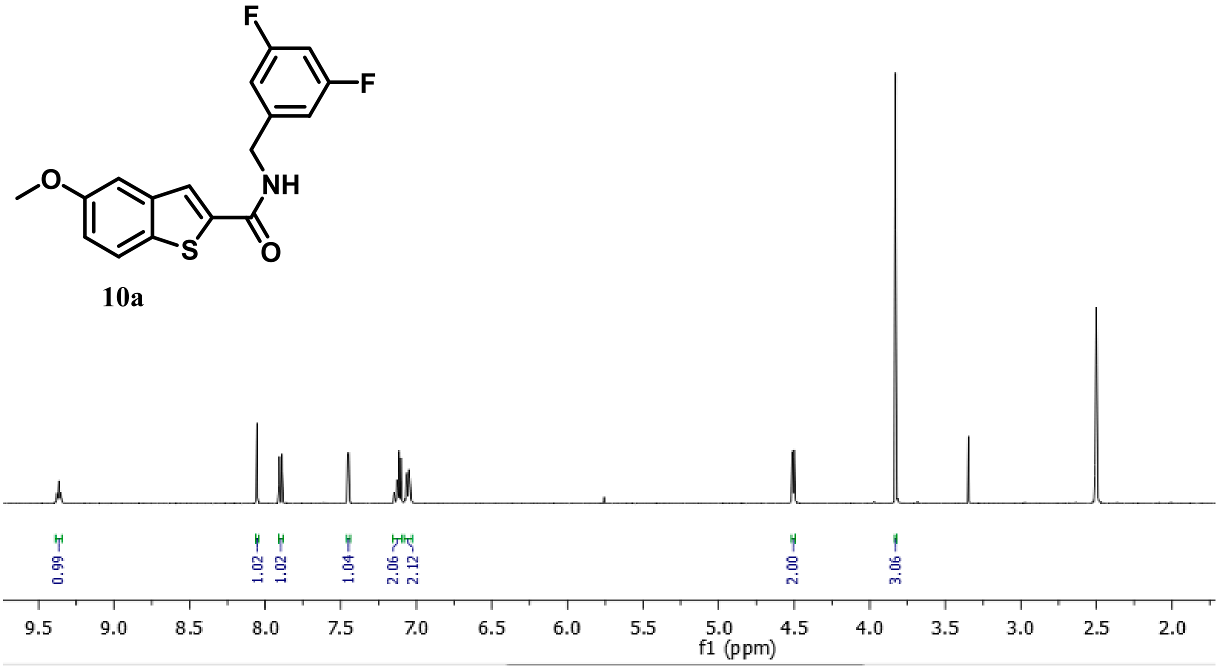
F
F
O
NH
S
O
10a
0.99
1.02
1.02
1.04
2.06
2.12
2.00
3.06
9.5 9.0 8.5 8.0 7.5 7.0 6.5 6.0 5.5 5.0 4.5 4.0 3.5 3.0 2.5 2.0
f1 (ppm)

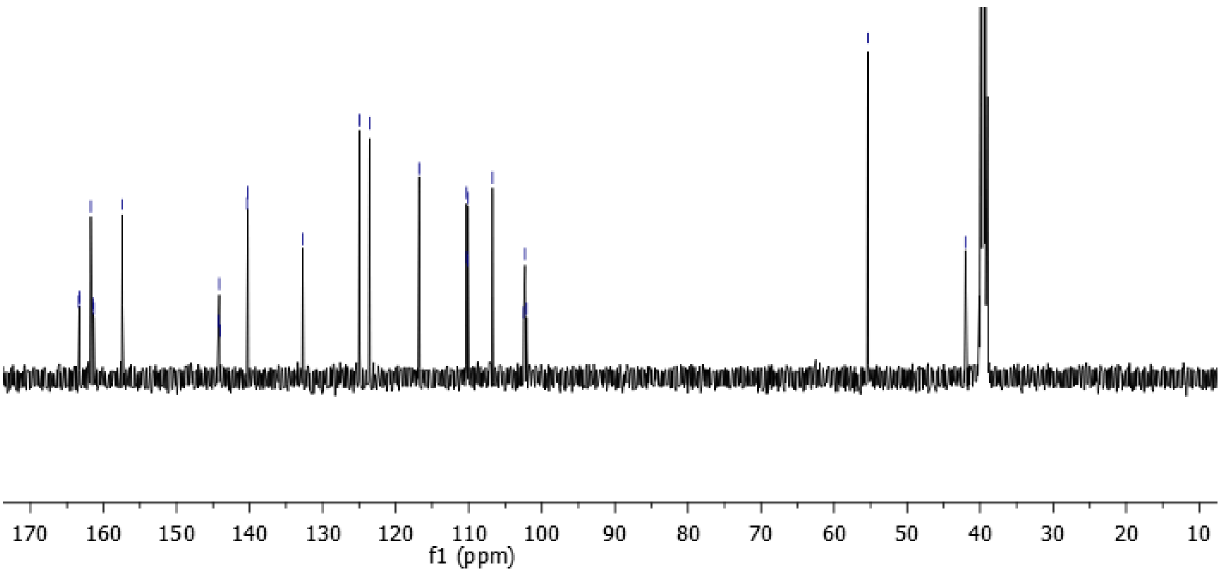
170 160 150 140 130 120 110 100 90 80 70 60 50 40 30 20 10
f1 (ppm)

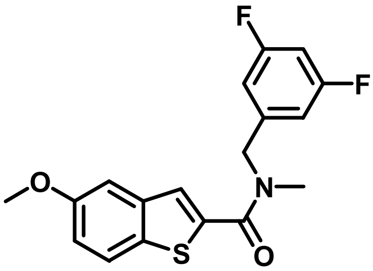
F
F
O
N
S
O

10b

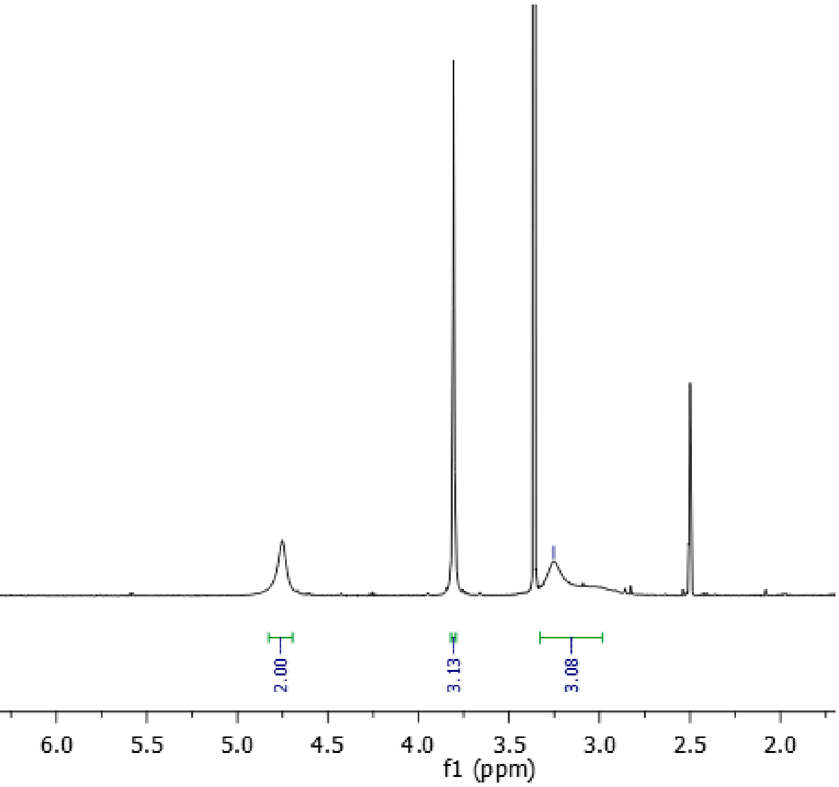
1.16
1.11
1.11
1.16
2.07
2.00
3.13
3.08
8.0 7.5 7.0 6.5 6.0 5.5 5.0 4.5 4.0 3.5 3.0 2.5 2.0
f1 (ppm)

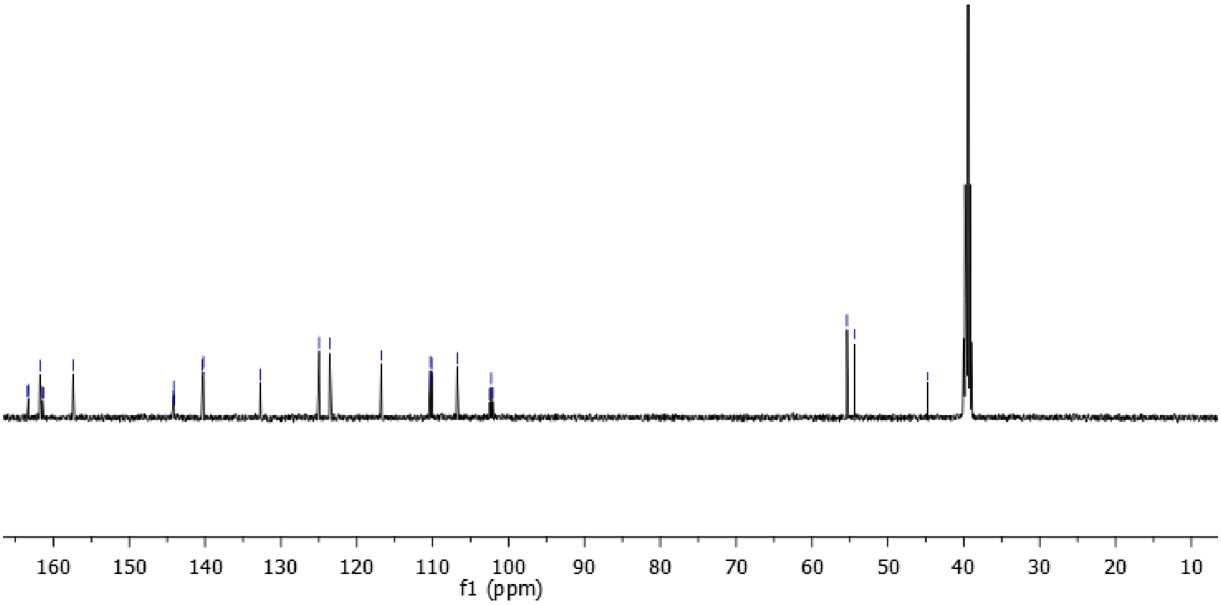
160 150 140 130 120 110 100 90 80 70 60 50 40 30 20 10
f1 (ppm)

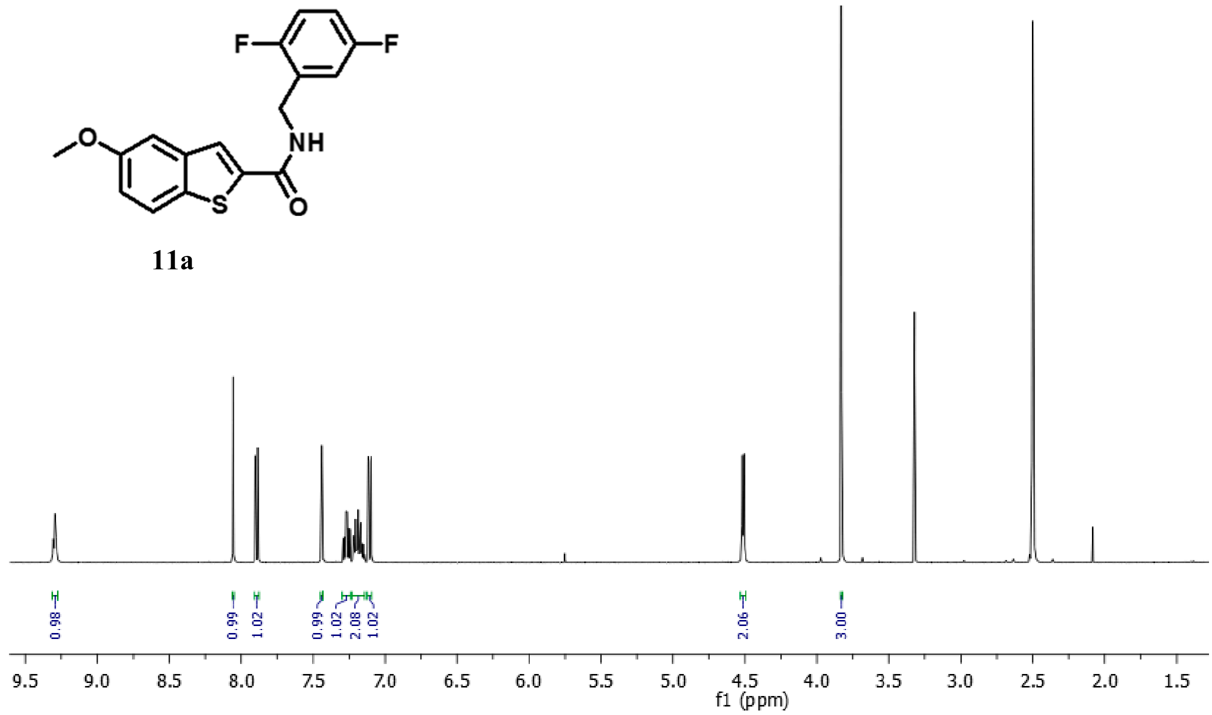

11a

0.98
0.99
1.02
0.99
1.02
2.08
1.02
2.06
3.00
9.5 9.0 8.5 8.0 7.5 7.0 6.5 6.0 5.5 5.0 4.5 4.0 3.5 3.0 2.5 2.0 1.5
f1 (ppm)

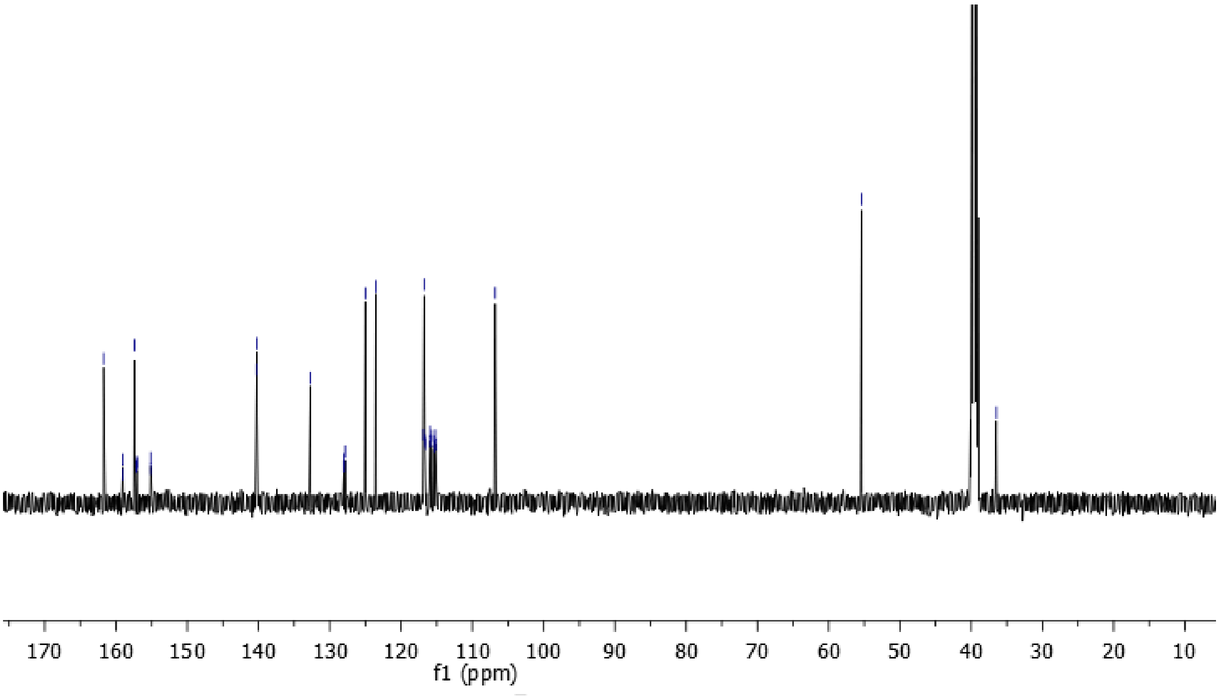

170 160 150 140 130 120 110 100 90 80 70 60 50 40 30 20 10
f1 (ppm)

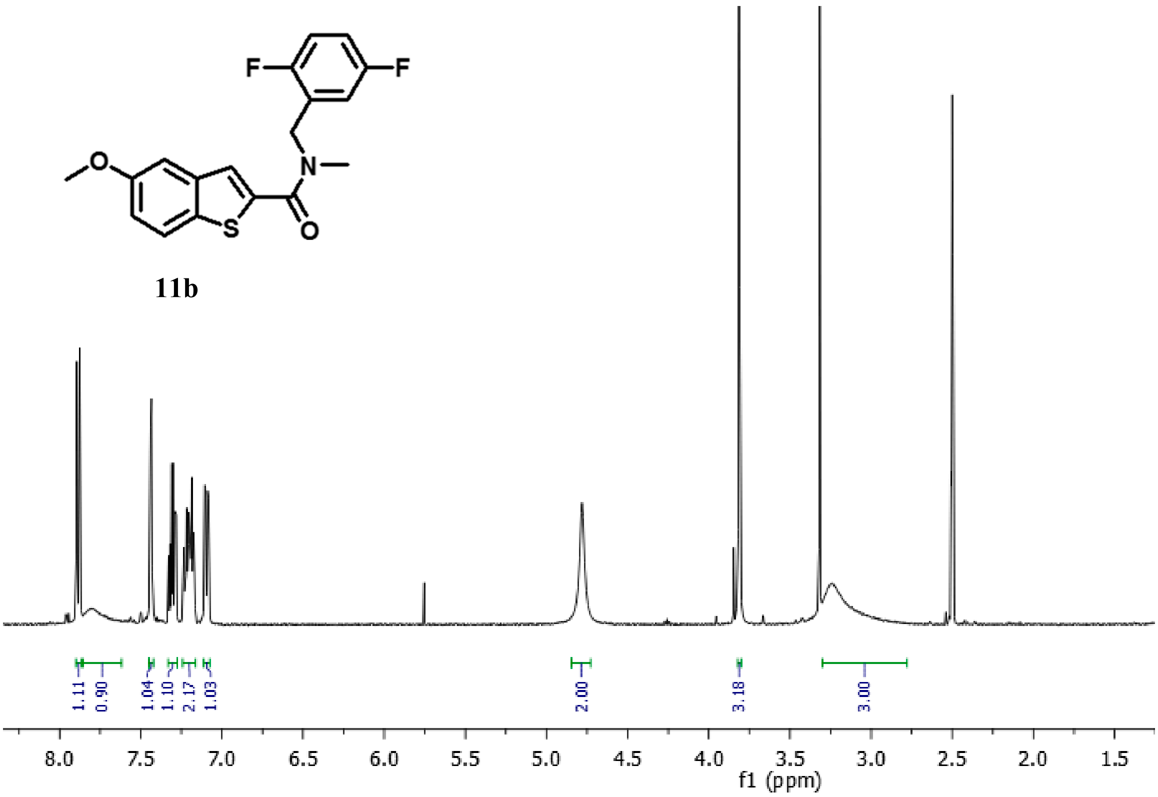
F
F
O
N
S
O
11b
1.11
0.90
1.04
1.10
2.17
1.03
2.00
3.18
3.00
8.0 7.5 7.0 6.5 6.0 5.5 5.0 4.5 4.0 3.5 3.0 2.5 2.0 1.5
f1 (ppm)

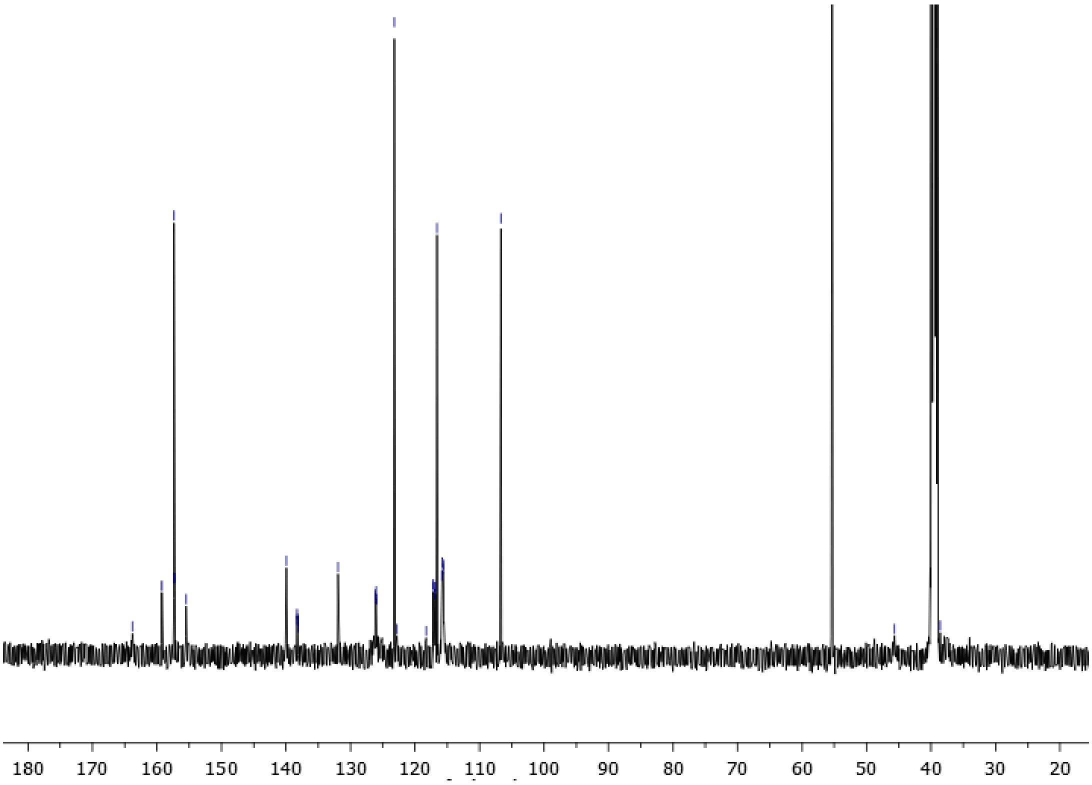
180 170 160 150 140 130 120 110 100 90 80 70 60 50 40 30 20

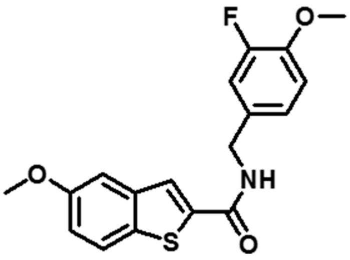

12a

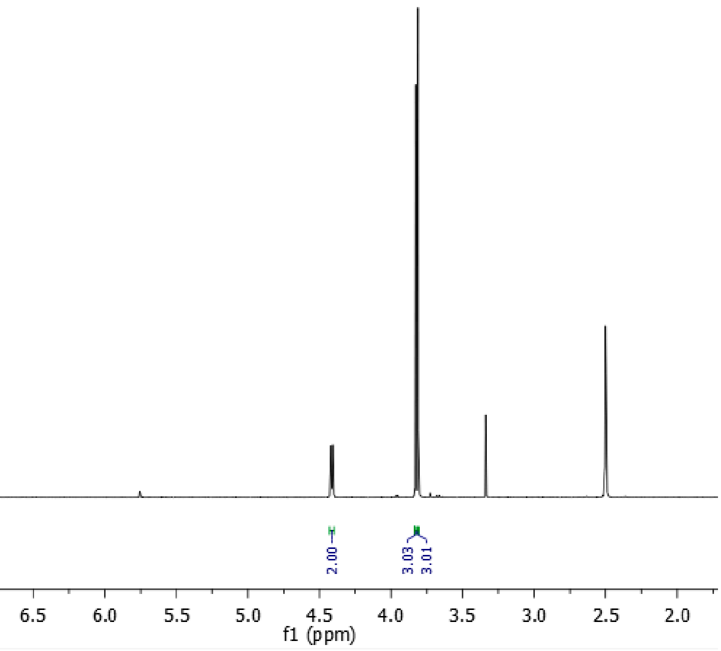
0.99
1.00
1.00
1.00
2.75
1.16
2.00
3.03
3.01
9.5 9.0 8.5 8.0 7.5 7.0 6.5 6.0 5.5 5.0 4.5 4.0 3.5 3.0 2.5 2.0
f1 (ppm)

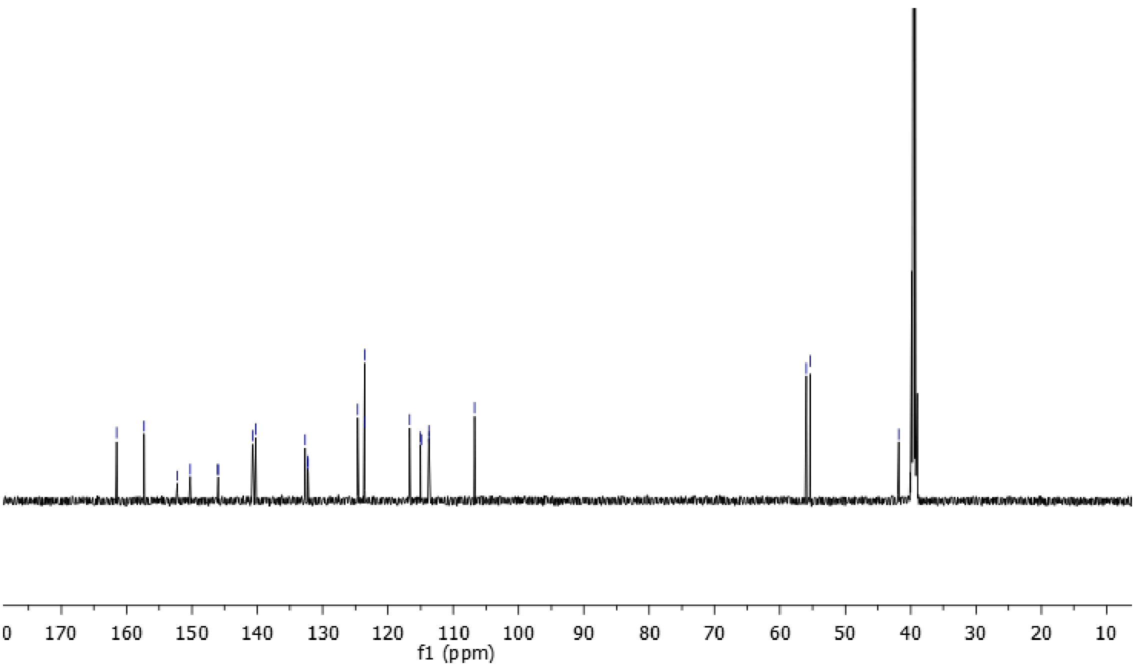
0 170 160 150 140 130 120 110 100 90 80 70 60 50 40 30 20 10
f1 (ppm)

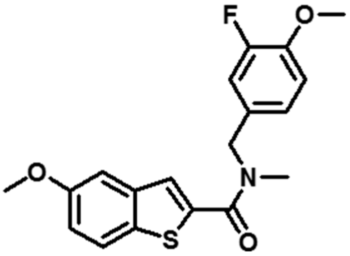
F
O
N
O
S
O

12b

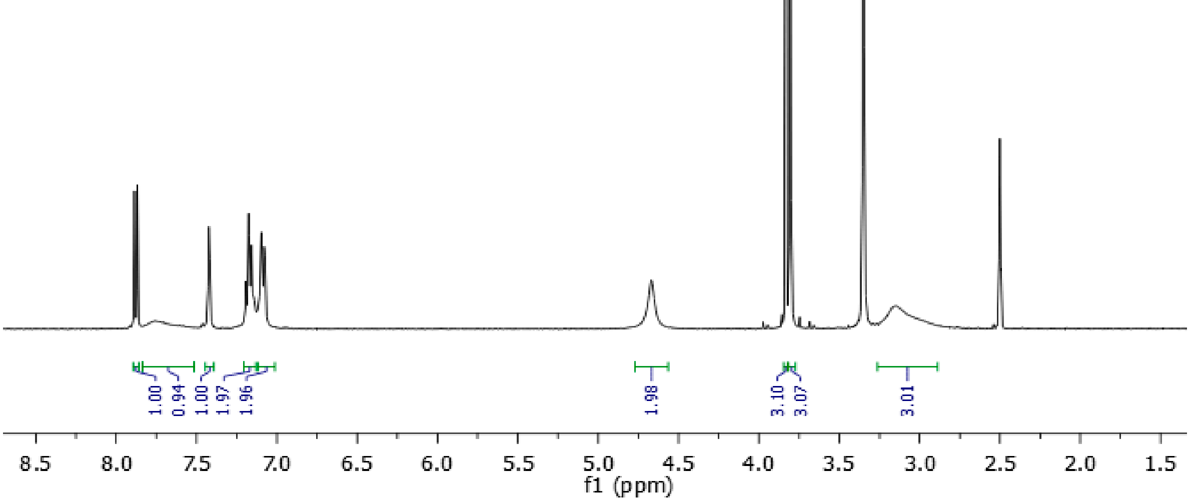
1.00
0.94
1.00
1.97
1.96
1.98
3.10
3.07
3.01
8.5
8.0
7.5
7.0
6.5
6.0
5.5
5.0
4.5
4.0
3.5
3.0
2.5
2.0
1.5
f1 (ppm)

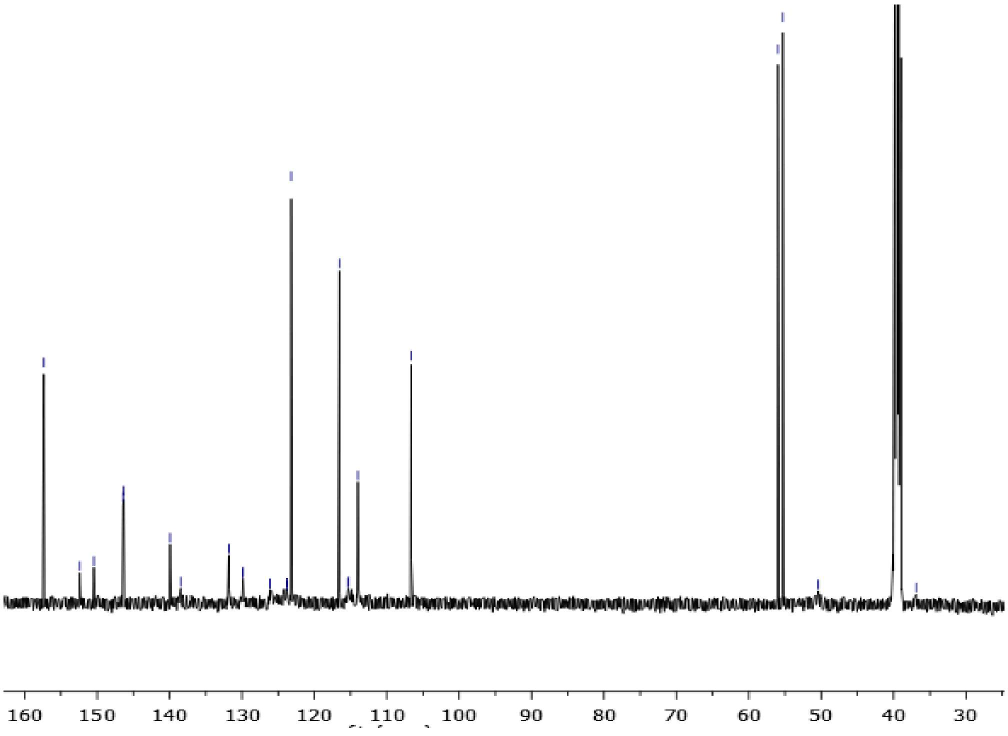
160
150
140
130
120
110
100
90
80
70
60
50
40
30

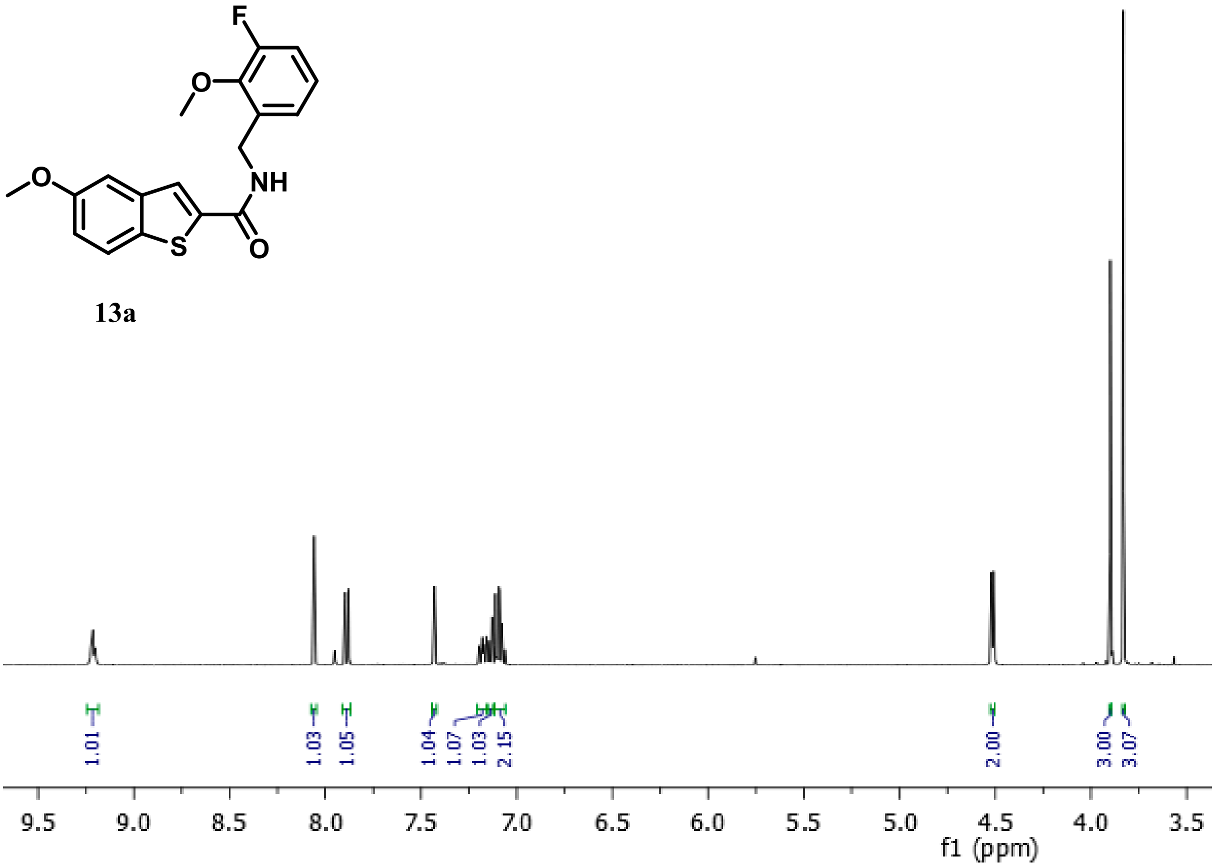
F
O
NH
O
O
S
13a
1.01
1.03
1.05
1.04
1.07
1.03
2.15
2.00
3.00
3.07
9.5 9.0 8.5 8.0 7.5 7.0 6.5 6.0 5.5 5.0 4.5 4.0 3.5
f1 (ppm)

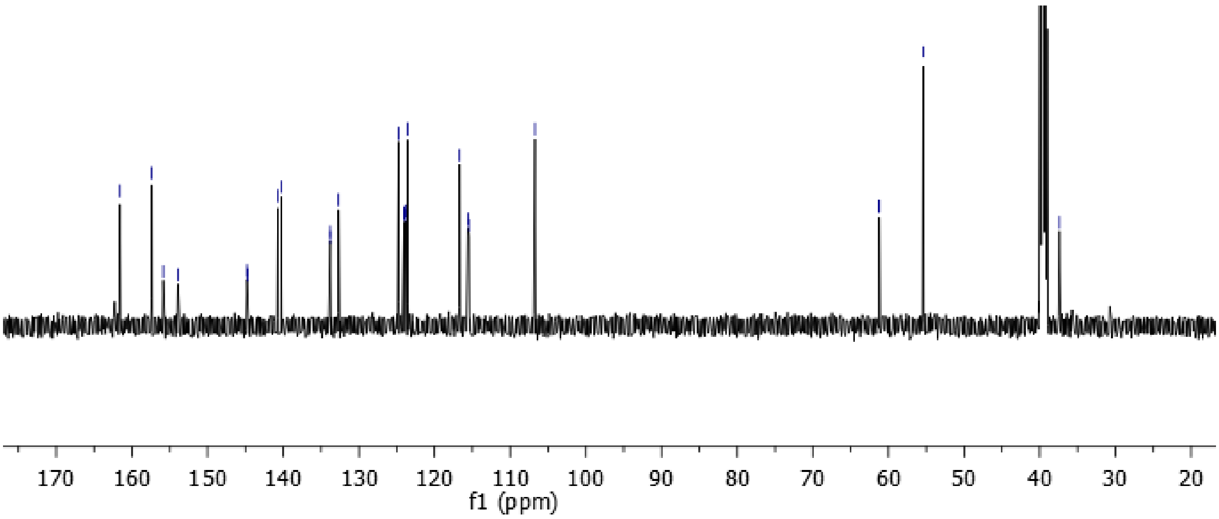
170 160 150 140 130 120 110 100 90 80 70 60 50 40 30 20
f1 (ppm)

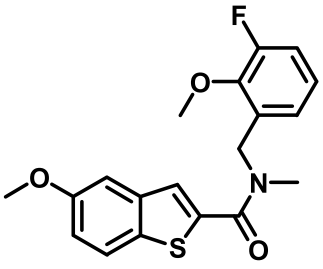
F
O
O
N
S
O

13b

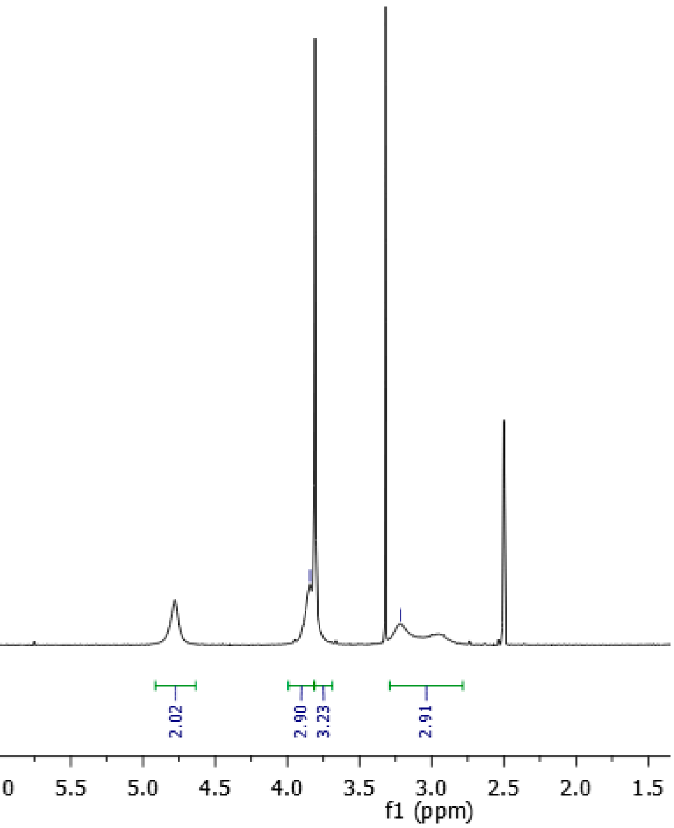
1.00
0.90
1.00
0.99
1.06
1.80
2.02
2.90
3.23
2.91
8.0 7.5 7.0 6.5 6.0 5.5 5.0 4.5 4.0 3.5 3.0 2.5 2.0 1.5
f1 (ppm)

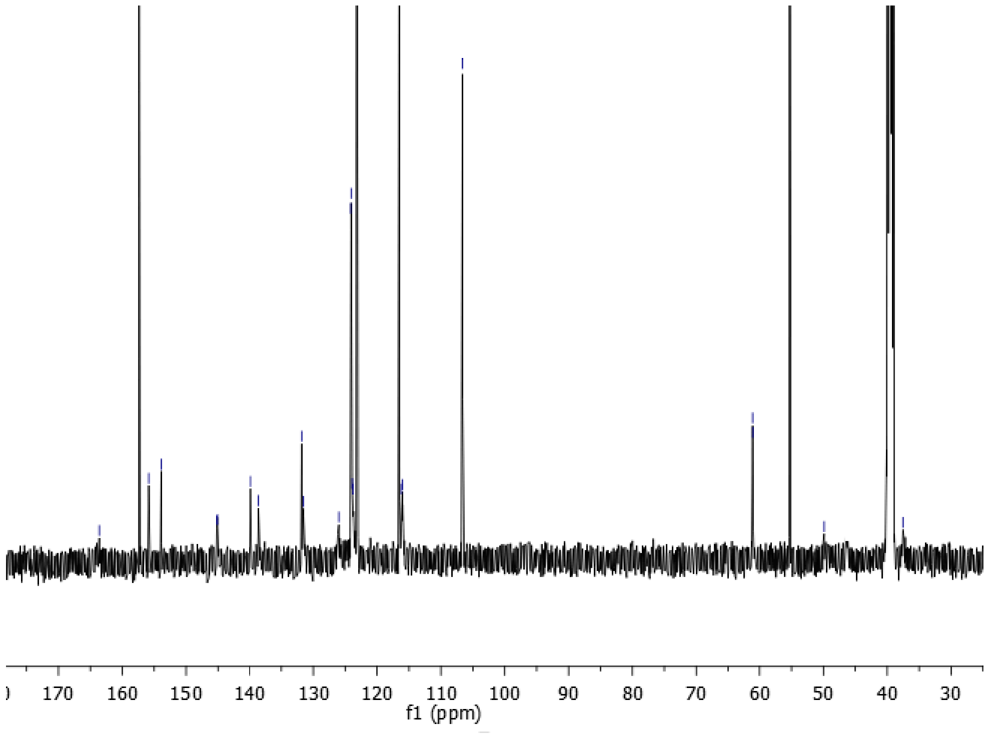
170 160 150 140 130 120 110 100 90 80 70 60 50 40 30
f1 (ppm)

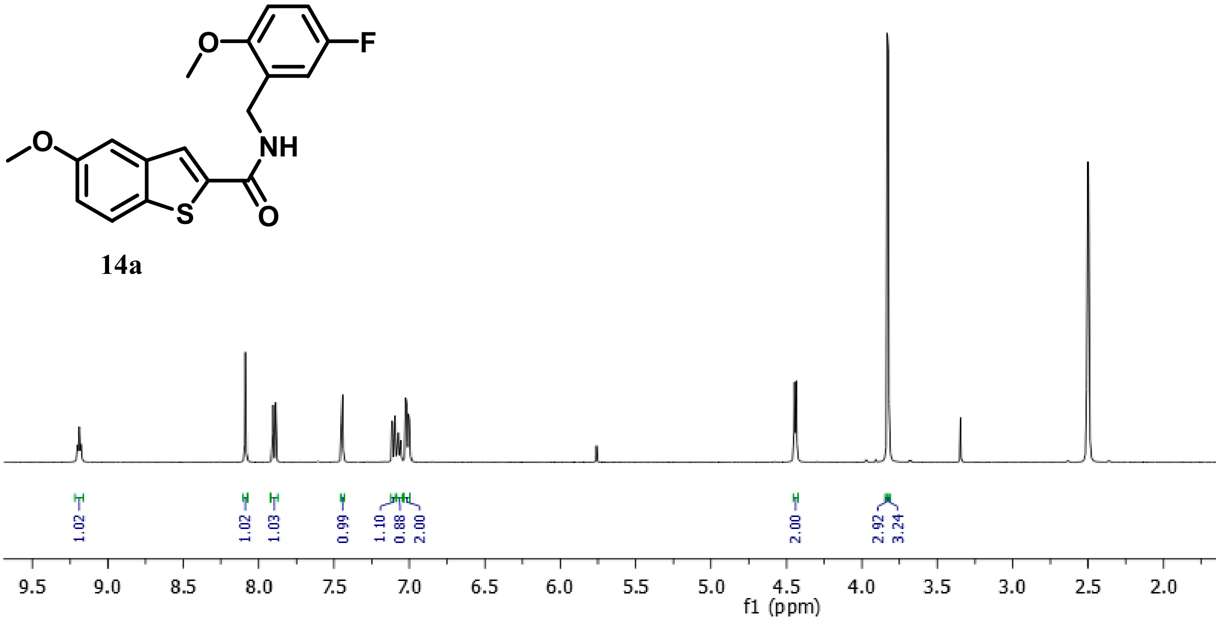
O
F
O
NH
S
O
14a
1.02
1.02
1.03
0.99
1.10
0.88
2.00
2.00
2.92
3.24
9.5 9.0 8.5 8.0 7.5 7.0 6.5 6.0 5.5 5.0 4.5 4.0 3.5 3.0 2.5 2.0
f1 (ppm)

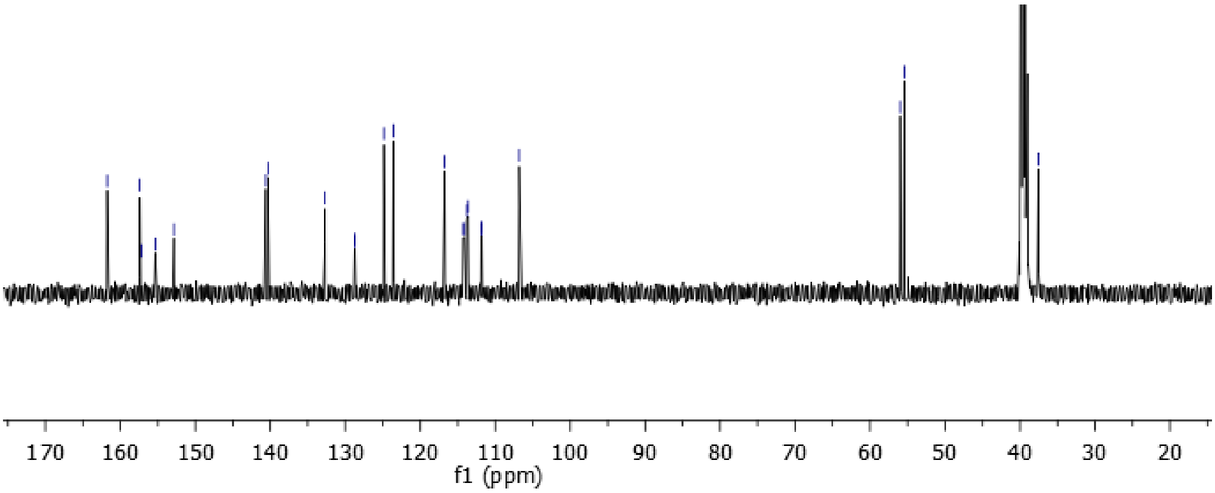
170 160 150 140 130 120 110 100 90 80 70 60 50 40 30 20
f1 (ppm)

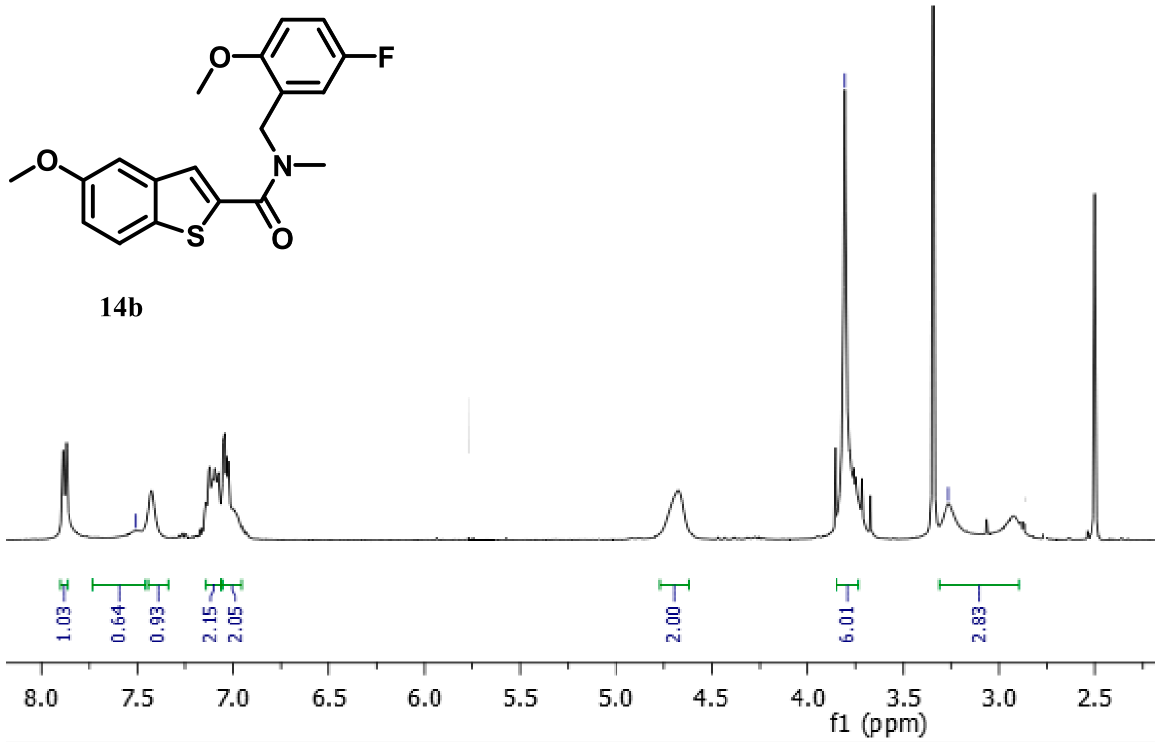

O
F
O
N
S
O
1.03
0.64
0.93
2.15
2.05
2.00
6.01
2.83
8.0 7.5 7.0 6.5 6.0 5.5 5.0 4.5 4.0 3.5 3.0 2.5
f1 (ppm)

14b

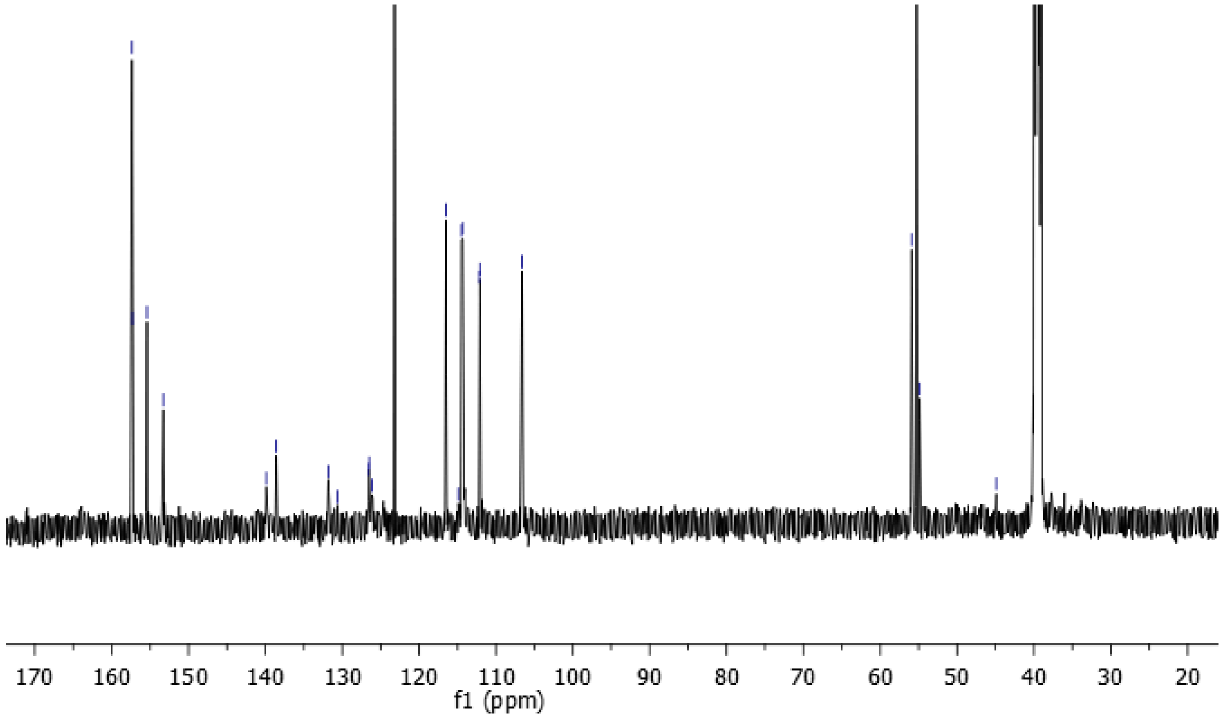

170 160 150 140 130 120 110 100 90 80 70 60 50 40 30 20
f1 (ppm)

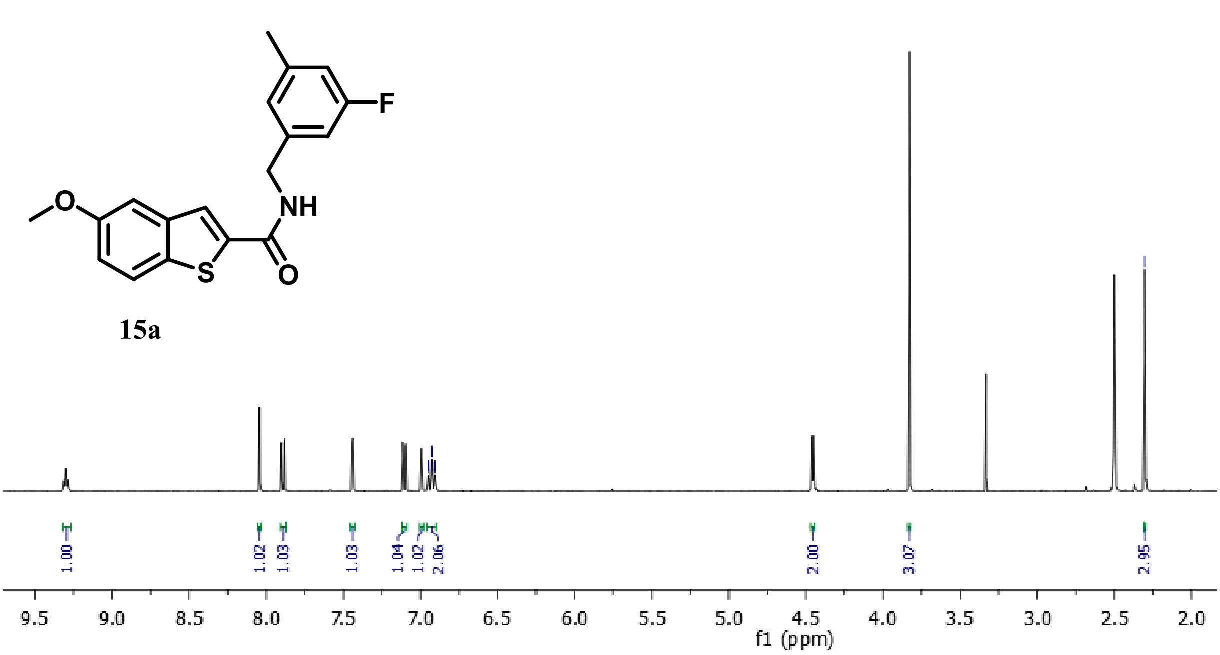
F
O
NH
S
O

15a

1.00
1.02
1.03
1.03
1.04
1.02
2.06
2.00
3.07
2.95
9.5 9.0 8.5 8.0 7.5 7.0 6.5 6.0 5.5 5.0 4.5 4.0 3.5 3.0 2.5 2.0
f1 (ppm)

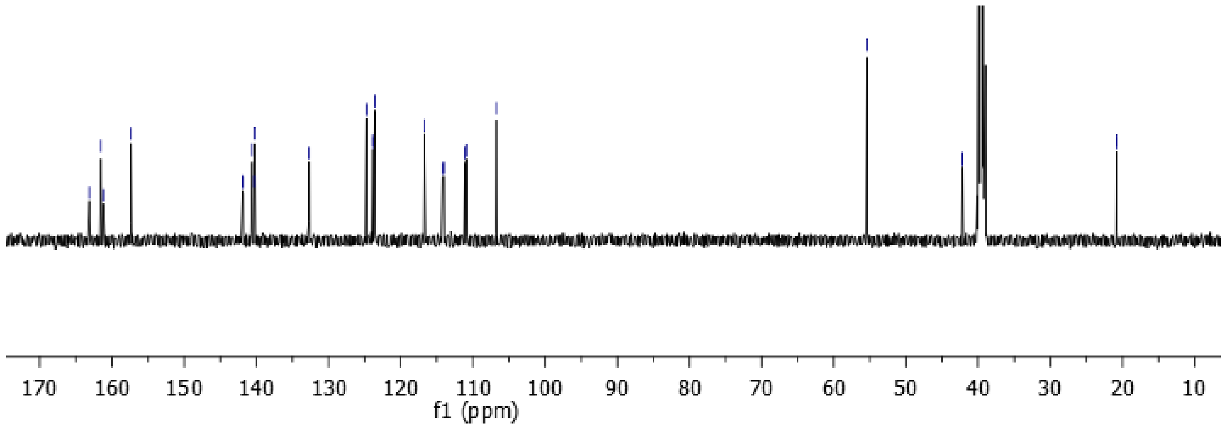
170 160 150 140 130 120 110 100 90 80 70 60 50 40 30 20 10
f1 (ppm)

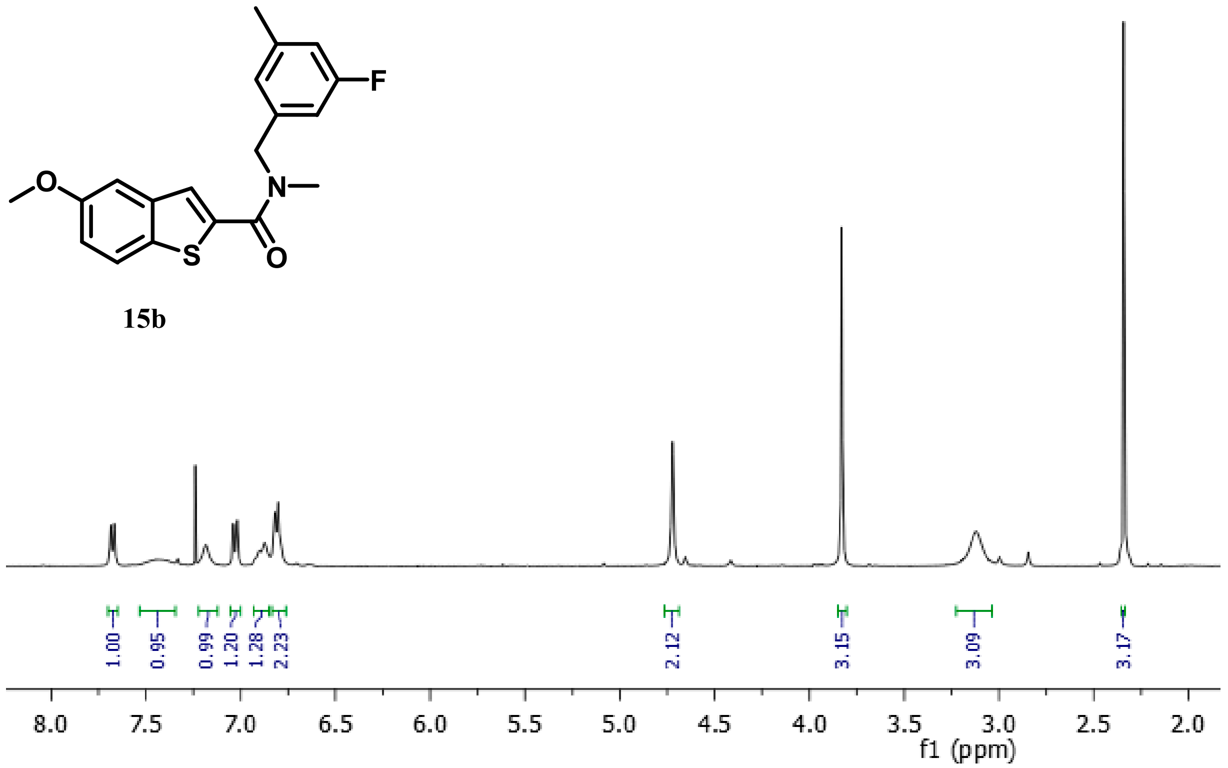

15b
1.00
0.95
0.99
1.20
1.28
2.23
2.12
3.15
3.09
3.17
8.0 7.5 7.0 6.5 6.0 5.5 5.0 4.5 4.0 3.5 3.0 2.5 2.0
f1 (ppm)

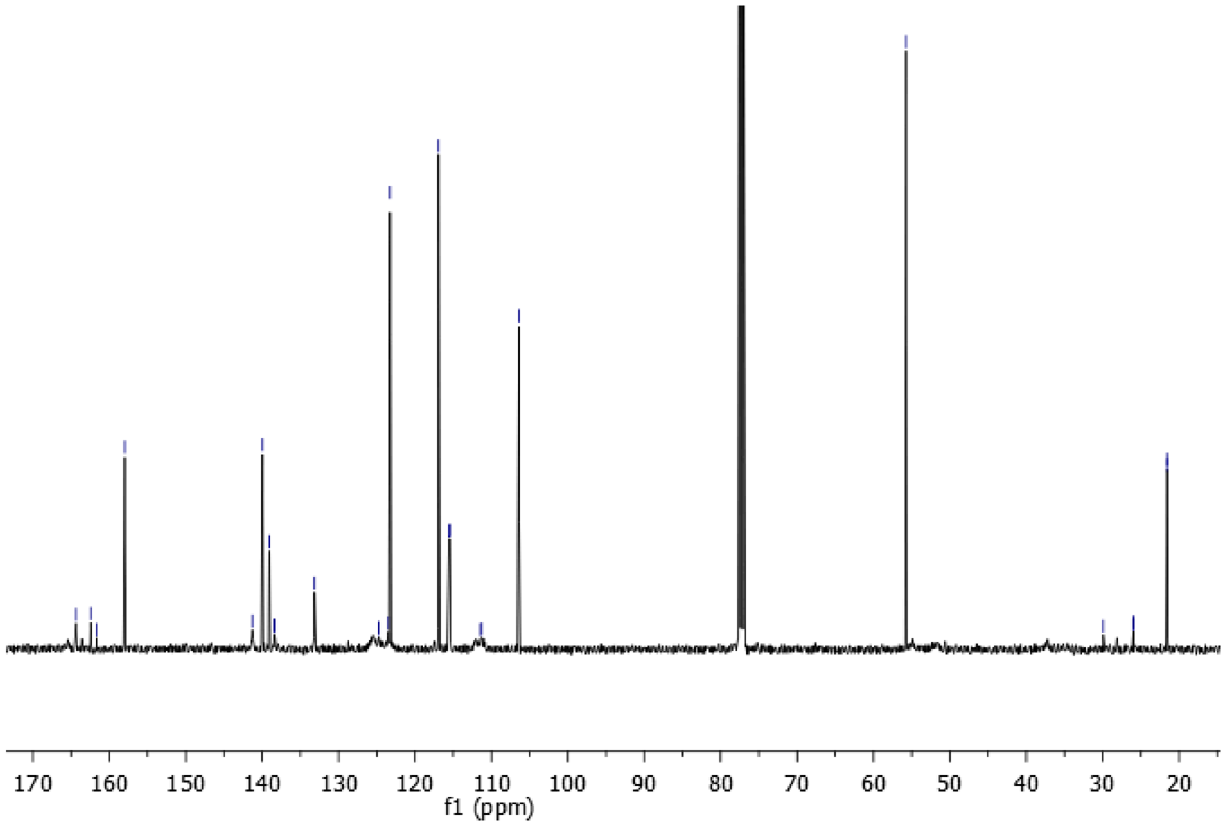
170 160 150 140 130 120 110 100 90 80 70 60 50 40 30 20
f1 (ppm)
